# Antimicrobial activity of linear lipopeptides derived from BP100 towards plant pathogens

**DOI:** 10.1371/journal.pone.0201571

**Published:** 2018-07-27

**Authors:** Àngel Oliveras, Aina Baró, Laura Montesinos, Esther Badosa, Emilio Montesinos, Lidia Feliu, Marta Planas

**Affiliations:** 1 LIPPSO, Department of Chemistry, University of Girona, Campus Montilivi, Girona, Spain; 2 Laboratory of Plant Pathology, Institute of Food and Agricultural Technology-CIDSAV-XaRTA, University of Girona, Campus Montilivi, Girona, Spain; Stony Brook University, UNITED STATES

## Abstract

A collection of 36 lipopeptides were designed from the cecropin A-melittin hybrid peptide **BP100 (**H-Lys-Lys-Leu-Phe-Lys-Lys-Ile-Leu-Lys-Tyr-Leu-NH_2_) previously described with activity against phytopathogenic bacteria. These lipopeptides were synthesized on solid-phase and screened for their antimicrobial activity, toxicity and proteolytic stability. They incorporated a butanoyl, a hexanoyl or a lauroyl group at the N-terminus or at the side chain of a lysine residue placed at each position of the sequence. Their antimicrobial activity and hemolysis depended on the fatty acid length and its position. In particular, lipopeptides containing a butanoyl or a hexanoyl chain exhibited the best biological activity profile. In addition, we observed that the incorporation of the acyl group did not induce the overexpression of defense-related genes in tomato. Best lipopeptides were **BP370**, **BP378**, **BP381**, **BP387** and **BP389**, which were highly active against all the pathogens tested (minimum inhibitory concentration of 0.8 to 12.5 μM), low hemolytic, low phytotoxic and significantly stable to protease degradation. This family of lipopeptides might be promising functional peptides useful for plant protection.

## Introduction

Plant pathogenic bacteria and fungi cause a vast amount of diseases of crops giving rise to economic losses and affecting the quality and safety of food [1–3]. The currently used antimicrobial agents to combat plant diseases have non-target effects on consumers and environment mainly due to their toxicity and low biodegradability [4]. Moreover, the development of resistance by bacteria and fungi also constitutes an important hurdle associated to the use of antimicrobials in plant protection [5,6]. Therefore, there is a need for finding out safer chemicals to control plant pathogens that overcome these limitations.

Antimicrobial and plant defense elicitor peptides have been the object of an intense research and development as new candidates for plant protection against fungal and bacterial diseases [7–12]. Lipopeptides are a subclass of antimicrobial peptides that are considered attractive candidates for the development of new peptide-based pesticides [13–15]. They are composed of a linear or cyclic peptide sequence incorporating an acyl chain, generally attached to the N-terminus [13,14,16–23]. Lipopeptides display a broad spectrum of activity and, similarly to antimicrobial peptides, their mode of action involves the perturbation of the cell membrane. These compounds first interact electrostatically with the negatively charged bacterial membrane which is followed by their insertion into the hydrophobic core of the cell membrane, disturbing the bilayer integrity by forming non-specific channels/pores. It has also been described that lipopeptides may inhibit the synthesis of essential cell wall components [19,22]. These peptides are unlikely to cause the emergence of pathogen resistant strains because they do not target a specific receptor [19,21].

The acyl chain is crucial for the biological activity of lipopeptides, because it acts as a membrane anchor [21,24–27]. In this context, acylation of a peptide sequence is regarded as a means of increasing membrane affinity and, consequently, antimicrobial activity [21,26,28–33]. Remarkably, this strategy was also described to endow non-active peptides with antimicrobial activity [34–37]. Moreover, it has been reasoned that the presence of the acyl chain protects peptides from proteolytic degradation [21,38–40].

Despite the excellent biological properties of lipopeptides, up to now only few reports are centered on their development as agents for plant protection. Most of the research have been focused on cyclic lipopeptides produced by *Bacillus subtilis* strains due to their diversity of biological properties which include the capacity of inducing plant defense responses [13–18]. Recently, we described synthetic cyclic lipodecapeptides derived from the lead antimicrobial peptide **BPC194** that were active against plant pathogenic bacteria and fungi, exhibiting differential hemolysis and phytotoxicity [10]. In addition, synthetic acyl linear tetrapeptides have been reported effective to control plant pathogens both *in vitro* and *in planta* [41].

Within our continuous exploration of efficient agents to control plant diseases caused by plant pathogenic bacteria such as *Erwinia amylovora*, *Xanthonomas axonopodis* pv. vesicatoria, and *Pseudomonas syringae* pv. syringae, and the fungi *Penicillium expansum* and *Fusarium oxysporum*, we identified the linear undecapeptide H-Lys-Lys-Leu-Phe-Lys-Lys-Ile-Leu-Lys-Tyr-Leu-NH_2_ (**BP100**) with high antimicrobial activity, low hemolysis and phytotoxicity, and reasonable susceptibility to protease degradation [42,43]. Based on the above considerations on lipopeptides and with the aim of improving the biological profile of **BP100**, we decided to study the influence in antimicrobial properties of incorporating an acyl chain in its sequence. Moreover, we are also exploring the use of peptides as plant defense elicitors as a new strategy for plant disease management. In particular, we have recently identified linear undecapeptides and cyclic decapeptides able to induce defense responses on tobacco cells and tomato plants, that efficiently control fire blight infections caused by the bacteria *E*. *amylovora* on pear [44].

Herein, we report the design, synthesis and properties of a series of 36 lipopeptides derived from **BP100**. We evaluated the effect of the length and position of the hydrophobic chain on the antimicrobial activity, toxicity and stability. In addition, we studied the capacity of selected lipopeptides to induce the expression of defense-related genes on tomato plants.

## Materials and methods

### General methods

Manual peptide synthesis was performed in polypropylene syringes (2 or 5 mL) fitted with a polyethylene porous disk. Solvents and soluble reagents were removed by suction. Most chemicals were purchased from commercial suppliers Sigma–Aldrich (Madrid, Spain), Iris Biotech GmbH (Marktredwitz, Germany), Scharlab (Sentmenat, Spain) or Panreac (Castellar del Vallès, Spain), and used without further purification.

Peptides were analyzed under standard analytical HPLC conditions with a Dionex liquid chromatography instrument composed of an UV/Vis Dionex UVD170U detector, a P680 Dionex pump, an ASI-100 Dionex automatic injector, and CHROMELEON 6.60 software. Detection was performed at a wavelength of 220 nm. Solvent A was 0.1% aqueous TFA and solvent B was 0.1% TFA in CH_3_CN. Analyses were carried out with a Kromasil 100 C_18_ (4.6 mm × 40 mm, 3 μm) column with a linear gradient of 2–100% B over 7 min at a flow rate of 1 mL/min. Peptides were also analysed with a 1260 Infinity II liquid chromatography instrument (Agilent Technologies) composed of a Diode Array Detector HS, a Quaternary Pump VL, a 1260 Vial sampler and OpenLab CDS ChemStation software. Analyses were carried out with a linear gradient of 2–100% B over 12 min at a flow rate of 1 mL/min.

All purifications were performed on a Combi*Flash* Rf200 automated flash chromatography system using Redi*Sep* Rf Gold reversed-phase C_18_ column packed with high performance C_18_ derivatized silica.

ESI-MS analyses were performed at the Serveis Tècnics de Recerca of the University of Girona with an Esquire 6000 ESI ion Trap LC/MS (Bruker Daltonics) instrument equipped with an electrospray ion source. The instrument was operated in the positive ESI(+) ion mode. Samples (5 μL) were introduced into the mass spectrometer ion source directly through an HPLC autosampler. The mobile phase (80:20 CH_3_CN/H_2_O at a flow rate of 100 μL/min) was delivered by a 1200 Series HPLC pump (Agilent). Nitrogen was employed as both the drying and nebulising gas.

HRMS were recorded on a Bruker MicroTof-QIITM instrument using ESI ionization source at the Serveis Tècnics de Recerca of the University of Girona. Samples were introduced into the mass spectrometer ion source by direct infusion using a syringe pump and were externally calibrated using sodium formate. The instrument was operated in the positive ion mode.

### Synthesis of lipopeptides

These lipopeptides were synthesized manually by the solid-phase method using standard Fmoc chemistry. The Fmoc‐Rink-MBHA resin (0.56 mmol/g) was used as solid support. Fmoc-Leu‐OH, Fmoc-Lys(Boc)-OH, Fmoc-Lys(ivDde)-OH, Fmoc-Phe‐OH, Fmoc-Ile-OH, and Fmoc-Tyr(*t*Bu)-OH were used as amino acid derivatives. Peptide elongation was carried out through sequential Fmoc removal and coupling of the corresponding amino acid. Fmoc group removal was achieved with piperidine/DMF (3:7, 2 + 10 min). Couplings of the Fmoc-amino acids (4 equiv.) were mediated by Oxyma (4 equiv.) and DIC (4 equiv.) in DMF at room temperature for 1 h under stirring. The completion of the reactions was checked with the Kaiser test [45]. After each coupling and deprotection step, the resin was washed with DMF (6 × 1 min) and CH_2_Cl_2_ (2 × 1 min). Once the peptide elongation was completed the peptidyl resin was treated with piperidine/NMP (3:7, 2 + 10 min), washed with NMP (6 × 1 min), and CH_2_Cl_2_ (2 × 1 min), and air dried.

For lipopeptides **BP367**, **BP379** and **BP391** the N-terminal deprotected resin was acylated by treatment with the corresponding fatty acid (10 equiv.), DIC (10 equiv.) and Oxyma (10 equiv.) in NMP for 1 h under stirring. After this time, the resin was washed with NMP (6 × 1 min) and CH_2_Cl_2_ (6 × 1 min), and air dried. Completion of the reactions was checked with the Kaiser test [45].

In the case of the side-chain acylated derivatives, the N-terminal deprotected resin was acetylated with Ac_2_O/pyridine/CH_2_Cl_2_ (1:1:1) for 1 h, washed with NMP (6 × 1 min) and CH_2_Cl_2_ (6 × 1 min), and air dried. Completion of the reactions was checked with the Kaiser test [45]. The resulting resin was treated with NH_2_NH_2_·H_2_O/NMP (2:98, 10 × 20 min) under stirring and washed with NMP (2 × 1 min), CH_2_Cl_2_ (2 × 1 min), MeOH (2 × 1 min), and NMP (2 × 1 min). Then, the resin was acylated by treatment with the corresponding fatty acid (10 equiv.), DIC (10 equiv.) and Oxyma (10 equiv.) in NMP for 1 h under stirring. The resin was then washed with NMP (6 × 1 min) and CH_2_Cl_2_ (6 × 1 min), and air dried. Completion of the reactions was checked with the Kaiser test [45].

Finally, each resulting peptidyl resin was treated with TFA/H_2_O/TIS (95:2.5:2.5) for 2 h. Following TFA evaporation and diethyl ether extraction, the crude lipopeptides were purified by reverse-phase column chromatography, lyophilized, analysed by HPLC, and characterized by mass spectrometry.

### Bacterial and fungal strains and growth conditions

The following plant pathogenic bacterial strains were used: *Erwinia amylovora* PMV6076 (Institut National de la Recherche Agronomique, Angers, France), *Pseudomonas syringae* pv. syringae EPS94 (Institut de Tecnologia Agroalimentària, Universitat de Girona, Spain), *Xanthomonas axonopodis* pv. vesicatoria 2133–2, *Pseudomonas syringae* pv. actinidiae Psa3700.1.1, *Xanthomonas fragariae* Xf349-9A (Instituto Valenciano de Investigaciones Agrarias, Valencia, Spain), and *Xanthomonas arboricola* pv. pruni CFBP5563 (Collection Française de Bactéries associées aux Plantes, Angers, France). All bacteria except for *X*. *fragariae* were stored in Luria Bertani (LB) broth supplemented with glycerol (20%) and maintained at -80°C. For *X*. *fragariae*, Medium B [46] was used instead of LB. *E*. *amylovora*, *X*. *arboricola* pv. pruni, *P*. *syringae* pv. syringae and *P*. *syringae* pv. actinidiae were scrapped from the agar media after growing for 24 h at 25°C, and *X*. *axonopodis* pv. vesicatoria and *X*. *fragariae* after growing for 48 h at 25°C. The cell material was suspended in sterile water to obtain a suspension of 10^8^ CFU mL^-1^.

The following plant pathogenic fungal strains were used: *Penicillium expansum* EPS26 (Institut de Tecnologia Agroalimentària, Universitat de Girona, Spain) and *Fusarium oxysporum* f. sp. *lycopersici* FOL 3 race 2 (ATCC 201829, American Type Culture Collection,Virginia, EEUU). Strains were cultured on potato dextrose agar (PDA) plates (Difco). Conidia from *P*. *expansum* and microconidia from *F*. *oxysporum* were obtained from 5–7 day-old agar Potato Dextrose (PDA) cultures after growth at 25°C. Inoculum was prepared by scraping spore material from culture surfaces with a cotton swab and resuspending it in distilled water containing 0.5‰ of tween 80. The suspensions were filtered through Miracloth (Merk, Millipore) and the concentration of conidia was determined using a hemacytometer and adjusted to 10^4^ conidia mL^-1^ for *F*. *oxysporum* and to 10^3^ conidia mL^-1^ for *P*. *expansum*.

### Antimicrobial activity

Lyophilized peptides were solubilized in sterile Milli-Q water to a final concentration of 1 mM and filter sterilized through a 0.22-μm pore filter. For minimum inhibitory concentration (MIC) assessment, dilutions of the compounds were made to obtain a stock concentration of 250, 125, 62, 31, 16, 8 and 4 μM. For antibacterial activity 20 μL of each dilution were mixed in a microtiter plate well with 20 μL of the corresponding suspension of the bacterial indicator, 160 μL of Trypticase Soy Broth (TSB) (BioMèrieux, France) to a total volume of 200 μL. For antifungal activity 20 μL of each stock solution were mixed in a microtiter plate well with 80 μL of the corresponding suspension of the fungal pathogen and 100 μL of double concentrated Potato Dextrose Broth (PDB) to a total volume of 200 μL containing 0.003% w/v of chloramphenicol to prevent bacterial contamination.

Three replicates for each combination of strain, compound and concentration were used. Microbial growth was determined by optical density measurement at 600 nm (Bioscreen C, Labsystem, Helsinki, Finland). For antibacterial activity microplates were incubated at 25°C with 20 s shaking before hourly absorbance measurement for 48 h. For antifungal activity microplates were incubated at 22°C with 1 min shaking before absorbance measurement that were done every two hours during seven days. The experiment was repeated twice. The MIC was taken as the lowest compound concentration with no growth at the end of the experiment.

### Hemolytic activity

The hemolytic activity of the compounds was evaluated by determining hemoglobin release from erythrocyte suspensions of horse blood (5% vol/vol)(Oxoid) as previously described [42]. Blood was centrifuged at 6000g for 5 min, washed three times with TRIS buffer (10 mM TRIS, 150 mM NaCl, pH 7.2) and ten times diluted.

Compounds were solubilized in TRIS buffer at 750, 500, 300 and 100 μM and mixed with horse erythrocytes (1:1 v/v). The mixture was incubated under continuous shaking for 1 h at 37°C. Then, the tubes were centrifuged at 3500g for 10 min, 80 μL aliquots of the supernatant transferred to 100-well microplates (Bioscreen), diluted with 80 μL water, and the absorbance measured at 540 nm (Bioscreen). Complete hemolysis was obtained by the addition of melittin at 100 μM (Sigma-Aldrich Corporation, Madrid, Spain). The percentage of hemolysis (*H*) was calculated using the equation: *H* = 100×[(*Op*−*Ob*)/(*Om*−*Ob*)], where *Op* was the density for a given compound concentration, *Ob* for the buffer, and *Om* for the melittin positive control.

### Effect of peptide infiltration on tobacco leaves

**BP100** and the 36 lipopeptides were evaluated for their effect upon infiltration on tobacco leaves as described previously [47]. Peptide solutions of 50, 150 and 250 μM were infiltrated (100 μL) into the mesophylls of fully expanded tobacco leaves. Infiltrations were carried out in a single leaf, and for each peptide and dose, at least three leaves randomly distributed in different plants were inoculated. Control infiltrations with water (negative control) or melittin (positive control) at the same molar concentration were performed. The appearance of symptoms on the leaves was followed for 48 h after infiltration and measured as a lesion diameter.

### Effect of peptide treatment on induction of defense gene expression of tomato plants

Seeds of tomato cv. Rio Grande plants were sown in hydroponic seed plugs (rockwool), germinated and grown under controlled greenhouse conditions (25 ± 2°C, 16 h light / 15 ± 2°C, 8 h dark, and 60% RH). Two-week old seedlings (two cotyledons) were transplanted into rockwool plugs (7.5×7.5×6.5 cm, Grodan Iberica). The experimental design consisted of three replicates of three plants per treatment. After two weeks, tomato leaves were sprayed with aqueous solutions of **BP100**, 9 selected derivatives and **flg15** at 125 μM, jasmonic acid at 2.5 mM (Sigma-Aldrich, Sant Louis, Missouri, UE), and acybenzolar-*S*-methyl at 300 mg/L (Syngenta, Basel, Switzerland), until the run-off point. Water-sprayed plants were used as not treated controls. Twenty-four hours after product application leaf samples were collected and processed to extract RNA for RT-qPCR assays. Plant material was ground to a fine powder in liquid nitrogen with the Tissuelyzer II system (Qiagen). Total RNA was extracted from leaves using PureLink Plant RNA Reagent (Invitrogen, Life Technologies) according to the manufacturer’s manual. The RNA was solubilized in RNAse free water and was routinely subjected to DNAse treatment (Ambion® Turbo DNA-free™, Life Technologies) to remove any contaminant DNA. In each step, the RNA was quantified using a Nanodrop N-2000 spectrophotometer, and its integrity verified by denaturing agarose gel electrophoresis. First-strand of complementary DNA (cDNA) was generated from leave RNA using reverse transcriptase (High Capacity cDNA Reverse Transcription Kit, Invitrogen) according to the manufacturer’s manual.

To test gene defense induction in the treated tomato plants, a qPCR assay was performed. Quantitative PCR was carried out in a fluorometric thermal cycler (qPCR Quant Studio 5, Applied Biosystems) by using a Mix SYBR®Green PCR Master Mix (Applied Biosystems). The total reaction volume of this PCR reaction was 20 μL and the reaction mixture was 1 μL of each primer set at the adequate concentration, 10 μL of MixSyber Green, 6 μL of distilled water and 2 μL of cDNA. Melting curve analysis was performed after amplification to verify amplification specificity. A constitutive gene (*actin* gene) was used as reference control, and the following genes implicated in plant defense response were analyzed: Pathogenesis-related protein-1 (*PR1*), Harpin (*Harp*), Polyphenol oxidase (*PPO*), Subtilisin-like protease (*Sub1*), Blue copper binding-protein (*BCB*), Osmotin (*Osm2*), Acidic β-1,3 endoglucanase (*GluA*), Lypoxigenase (*LOX*), Protein inhibitor II (*PinII*), Dehydrin (*Tas14*) and Ethylene response transcription factor (*ERT3*). Specific oligonucleotides were used for the quantification of the target genes: *Harp* (f-ATTATGGCCCGTCCATTCCG; r- ATGCAATGACTCCGAGGACG), *GluA* (f-GGTCTCAACCGCGACATATT; r-CACAAGGGCATCGAAAAGAT), *PPO* (f-AGACGTAATTCCCACGTCCG; r-GGCACGGTACACCGAAGTTA), *Sub1* (f-ACCTAAAGGCGTTGTCGTGA; r-ACCCCAGACATTGAGCTGTT), *ERT3* (f-TCCGAAACAGTCACATCGCA; r-AGCATCTTCCGCGCTATCAA´), *BCB* (f-TTGGCACACACTGTCAAGGT; r-ACTGGCCAATAGGGTCGTTG), *Osm2* (f-TCCAATTCAATGCACAGCCA; r-TAGGACCACATGGACCGTGA), and the oligonucleotides used for the genes *PR1*, *LOX*, *PinII* and *Tas14* [44]. For each gene system, the concentration of the primer pair was optimized. The primer concentration was 100 nM for all the genes except for the *GluA*, *Harp*, *PR1* and actin genes which optimized concentration was 300 nM. A calibration curve was prepared by cloning the corresponding DNA in the pSpark cloning vector (Canvax, Córdoba, Spain) according to the manufacturer instructions, which was then used to transform *E*. *coli* DH5α by standard procedures. The number of plasmid copies were quantified after purification from *E*. *coli* (QIAGEN Iberia, S. L., Madrid, Spain), and appropriate dilutions were prepared to obtain the standard curve. The efficiency for each standard curve was calculated to check that the efficiency within amplifications was similar. Relative quantification of gene expression was done using the *ΔΔCt* method [48]. Each biological repetition treatment was analyzed in duplicate. The mean of the *Ct* values obtained were used to estimate the fold change value of the endogenous reference gene (actin) and the target plant defense genes. These results were used to calculate the ratios of the plant defense genes (relative to the actin gene, and for all treatments analyzed, including the control plants). The statistical significance of the results for the selected peptides was determined using the REST2009 Software [49].

### Susceptibility to protease degradation

Digestion of **BP100** and of 16 selected lipopeptides was carried out by treating 50 μg/mL peptide with 1 μg/mL proteinase K (Sigma-Aldrich Corporation, Spain) in 100 mM TRIS buffer, pH 7.6, at 25°C. The peptide cleavage after 60 min was determined by HPLC using a Kromasil (4.6 × 40 mm; 3 μm particle size) C_18_ reverse-phase column. Linear gradients of 0.1% aqueous TFA and 0.1% TFA in CH_3_CN were run from 0.98:0.02 to 0:1 over 12 min with UV detection at 220 nm. Digestion was estimated as the percentage of degraded lipopeptide calculated from the decrease of the HPLC peak area of the native peptide.

## Results

### Design and solid-phase synthesis of the lipopeptides

The linear lipopeptides of this study were designed based on the structure of H-Lys-Lys-Leu-Phe-Lys-Lys-Ile-Leu-Lys-Tyr-Leu-NH_2_ (**BP100**) [42] by acylating the N-terminus or by incorporating an acylated lysine at each position of the sequence (Table 1). In order to evaluate the influence of the hydrophobic chain length on the biological activity, butanoyl, hexanoyl and lauroyl groups were selected. These acyl groups were chosen because they led to the best results in a previous work based on cyclic lipopeptides with antimicrobial activity [10].

**Table 1:** Sequences, retention times and purities on HPLC, and mass spectrometry data of lipopeptides.

Peptide	Structure^a^	*t*_R_ (min)^b^	Purity (%)^c^	HRMS (ESI)		
					Calcd	Found
**BP367**	C_5_H_11_CO-KKLFKKILKYL-NH_2_	7.77	>99	C_78_H_137_N_17_O_13_ [M + 2H]^2+^	760.0285	760.0270
**BP368**	Ac-K(COC_5_H_11_)KLFKKILKYL-NH_2_	7.91	>99	C_80_H_139_N_17_O_14_ [M + 2H]^2+^	781.0338	781.0319
**BP369**	Ac-KK(COC_5_H_11_)LFKKILKYL-NH_2_	7.53	92	C_80_H_139_N_17_O_14_ [M + 2H]^2+^	781.0338	781.0316
**BP370**	Ac-KKK(COC_5_H_11_)FKKILKYL-NH_2_	7.06	>99	C_80_H_140_N_18_O_14_ [M + 2H]^2+^	788.5393	788.5380
**BP371**	Ac-KKLK(COC_5_H_11_)KKILKYL-NH_2_	7.11	>99	C_77_H_142_N_18_O_14_ [M + 2H]^2+^	771.5471	771.5471
**BP372**	Ac-KKLFK(COC_5_H_11_)KILKYL-NH_2_	7.64	>99	C_80_H_139_N_17_O_14_ [M + 2H]^2+^	781.0338	781.0336
**BP373**	Ac-KKLFKK(COC_5_H_11_)ILKYL-NH_2_	7.94	>99	C_80_H_139_N_17_O_14_ [M + 2H]^2+^	781.0338	781.0330
**BP374**	Ac-KKLFKKK(COC_5_H_11_)LKYL-NH_2_	6.91	>99	C_80_H_140_N_18_O_14_ [M + 2H]^2+^	788.5393	788.5393
**BP375**	Ac-KKLFKKIK(COC_5_H_11_)KYL-NH_2_	7.17	>99	C_80_H_140_N_18_O_14_ [M + 2H]^2+^	788.5393	788.5383
**BP376**	Ac-KKLFKKILK(COC_5_H_11_)YL-NH_2_	7.48	>99	C_80_H_139_N_17_O_14_ [M + 2H]^2+^	781.0338	781.0311
**BP377**	Ac-KKLFKKILKK(COC_5_H_11_)L-NH_2_	7.58	>99	C_77_H_142_N_18_O_13_ [M + 2H]^2+^	763.5496	763.5486
**BP378**	Ac-KKLFKKILKYK(COC_5_H_11_)-NH_2_	6.96	>99	C_80_H_140_N_18_O_14_ [M + 2H]^2+^	788.5393	788.5396
**BP379**	C_3_H_7_CO-KKLFKKILKYL-NH_2_	7.52	>99	C_76_H_133_N_17_O_13_ [M + 2H]^2+^	746.0129	746.0102
**BP380**	Ac-K(COC_3_H_7_)KLFKKILKYL-NH_2_	7.79	>99	C_78_H_135_N_17_O_14_ [M + 2H]^2+^	767.0182	767.0158
**BP381**	Ac-KK(*COC*_*3*_*H*_*7*_)LFKKILKYL-NH_2_	6.96	>99	C_78_H_135_N_17_O_14_ [M + 2H]^2+^	767.0182	767.0160
**BP382**	Ac-KKK(COC_3_H_7_)FKKILKYL-NH_2_	6.91	>99	C_78_H_136_N_18_O_14_ [M + 2H]^2+^	774.5236	774.5220
**BP383**	Ac-KKLK(COC_3_H_7_)KKILKYL-NH_2_	6.41	>99	C_75_H_138_N_18_O_14_ [M + 2H]^2+^	757.5314	757.5312
**BP384**	Ac-KKLFK(COC_3_H_7_)KILKYL-NH_2_	6.91	>99	C_78_H_135_N_17_O_14_ [M + 2H]^2+^	767.0182	767.0158
**BP385**	Ac-KKLFKK(COC_3_H_7_)ILKYL-NH_2_	7.02	>99	C_78_H_135_N_17_O_14_ [M + 2H]^2+^	767.0182	767.0189
**BP386**	Ac-KKLFKKK(COC_3_H_7_)LKYL-NH_2_	6.25	>99	C_78_H_136_N_18_O_14_ [M + 2H]^2+^	774.5236	774.5234
**BP387**	Ac-KKLFKKIK(COC_3_H_7_)KYL-NH_2_	6.49	>99	C_78_H_137_N_18_O_14_ [M + 3H]^3+^	516.6848	516.6839
**BP388**	Ac-KKLFKKILK(COC_3_H_7_)YL-NH_2_	6.80	>99	C_78_H_135_N_17_O_14_ [M + 2H]^2+^	767.0182	767.0160
**BP389**	Ac-KKLFKKILKK(COC_3_H_7_)L-NH_2_	6.73	>99	C_75_H_138_N_18_O_13_ [M + 2H]^2+^	749.5340	749.5328
**BP390**	Ac-KKLFKKILKYK(COC_3_H_7_)-NH_2_	6.27	>99	C_78_H_136_N_18_O_14_ [M + 2H]^2+^	774.5236	774.5216
**BP391**	C_11_H_23_CO-KKLFKKILKYL-NH_2_	8.06	>99	C_84_H_149_N_17_O_13_ [M + 2H]^2+^	802.0755	802.0738
**BP392**	Ac-K(COC_11_H_23_)KLFKKILKYL-NH_2_	8.25	>99	C_86_H_151_N_17_O_14_ [M + 2H]^2+^	823.0808	823.0808
**BP393**	Ac-KK(COC_11_H_23_)LFKKILKYL-NH_2_	7.68	>99	C_86_H_151_N_17_O_14_ [M + 2H]^2+^	823.0808	823.0789
**BP394**	Ac-KKK(COC_11_H_23_)FKKILKYL-NH_2_	7.32	97	C_86_H_152_N_18_O_14_ [M + 2H]^2+^	830.5862	830.5847
**BP395**	Ac-KKLK(COC_11_H_23_)KKILKYL-NH_2_	7.39	>99	C_83_H_154_N_18_O_14_ [M + 2H]^2+^	813.5940	813.5911
**BP396**	Ac-KKLFK(COC_11_H_23_)KILKYL-NH_2_	7.76	>99	C_86_H_151_N_17_O_14_ [M + 2H]^2+^	823.0808	823.0791
**BP397**	Ac-KKLFKK(COC_11_H_23_)ILKYL-NH_2_	8.10	97	C_86_H_151_N_17_O_14_ [M + 2H]^2+^	823.0808	823.0806
**BP398**	Ac-KKLFKKK(COC_11_H_23_)LKYL-NH_2_	7.26	>99	C_86_H_152_N_18_O_14_ [M + 2H]^2+^	830.5862	830.5852
**BP399**	Ac-KKLFKKIK(COC_11_H_23_)KYL-NH_2_	7.38	>99	C_86_H_152_N_18_O_14_ [M + 2H]^2+^	830.5862	830.5847
**BP400**	Ac-KKLFKKILK(COC_11_H_23_)YL-NH_2_	7.60	>99	C_86_H_151_N_17_O_14_ [M + 2H]^2+^	823.0808	823.0796
**BP401**	Ac-KKLFKKILKK(COC_11_H_23_)L-NH_2_	7.77	>99	C_83_H_154_N_18_O_13_ [M + 2H]^2+^	805.5966	805.5955
**BP402**	Ac-KKLFKKILKYK(COC_11_H_23_)-NH_2_	7.29	99	C_86_H_152_N_18_O_14_ [M + 2H]^2+^	830.5862	830.5866

^a^ COC_3_H_7_, butanoyl; COC_5_H_11_, hexanoyl; COC_11_H_23_, lauroyl.

^b^ HPLC retention time.

^c^ Percentage determined by HPLC at 220 nm after purification.

These lipopeptides were synthesized on solid phase as C-terminal amides following a standard 9-fluorenylmethoxycarbonyl (Fmoc)/*tert*-butyl (*t*Bu) strategy using Fmoc-Rink-MBHA as solid support. The Fmoc group was removed with piperidine/DMF and couplings of the amino acids were mediated by *N*,*N*-diisopropylcarbodiimide (DIC) and ethyl 2-cyano-2-(hydroxyimino)acetate (Oxyma). To obtain the lipopeptides incorporating the acyl group at the side-chain of a Lys residue, this amino acid was incorporated as Fmoc-Lys(ivDde)-OH. Once peptide chain elongation was completed, the N-terminal Fmoc group was removed. For the synthesis of **BP367**, **BP379** and **BP391**, the free amino group was derivatized with butanoic, hexanoic or lauric acid, respectively. In the case of the side-chain acylated derivatives, the N-terminal amine was acetylated and, after 1-(4,4-dimethyl-2,6-dioxocyclohex-1-ylidine)-3-methylbutyl (ivDde) group removal, the resulting peptidyl resins were acylated with the corresponding fatty acid. Acidolytic cleavage was performed with trifluoroacetic acid (TFA)/H_2_O/triisopropylsilane (TIS) and the crude mixtures were purified by reverse phase column chromatography. Lipopeptides were obtained in excellent HPLC purities and were characterized by mass spectrometry.

### Antimicrobial activity

**Fig 1:**
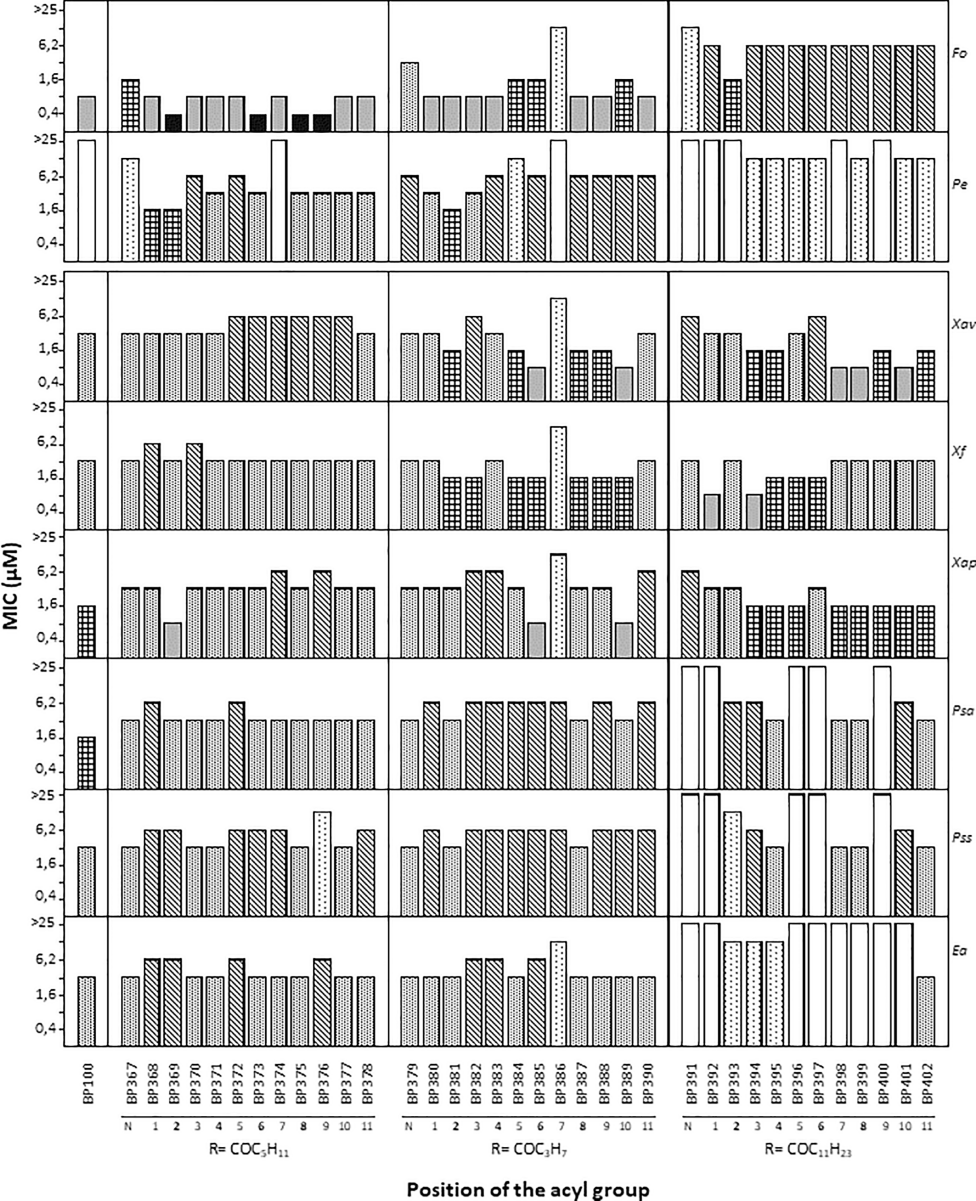
MICs of the lipopeptides derived from BP100 against the bacteria *E*. *amylovora* (*Ea*), *P*. *syringae* pv. syringae (*Pss*), *P*. *syringae* pv. actinidiae (*Psa*), *X*. *arboricola* pv. pruni (*Xap*), *X*. *fragariae* (*Xf*), and *X*. *axonopodis* pv. vesicatoria (*Xav*), and the fungi *P*. *expansum* (*Pe*) and *F*. *oxysporum* (*Fo*). The position of the acyl group at the N-terminus is indicated by a N, and the position of the acylated Lys by numbers 1 to 11. The type of the acyl group is depicted as R = COC_5_H_11_ (hexanoyl), R = COC_3_H_7_ (butanoyl) and R = COC_11_H_23_ (lauroyl).

Lipopeptides were tested for *in vitro* growth inhibition of the plant pathogenic bacteria *E*. *amylovora*, *P*. *syringae* pv. syringae, *P*. *syringae* pv. actinidiae, *X*. *arboricola* pv. pruni, *X*. *fragariae* and *X*. *axonopodis* pv. vesicatoria, and the plant pathogenic fungi *P*. *expansum* and *F*. *oxysporum*, at 0.4, 0.8, 1.6, 3.1, 6.2, 12.5 and 25 μM (Fig 1, Table A in S1 File).

Lipopeptides showed a high activity, 21 out of 36 sequences displayed MIC < 12.5 μM against all the pathogens tested. *X*. *arboricola* pv. pruni, *X*. *fragariae*, and *X*. *axonopodis* pv. vesicatoria were the most sensitive bacteria towards these compounds. Except for **BP386**, all compounds were active with MIC < 12.5 μM against these *Xanthomonas* species. Among them, 14 lipopeptides displayed MIC between 0.7 and 3.1 μM, against two of these three bacteria. These peptides incorporated a butanoyl or a lauroyl chain. Regarding *P*. *syringae* pv. syringae and *P*. *syringae* pv. actinidiae, 29 and 31 sequences exhibited MIC < 12.5 μM, respectively, displaying 12 of them MIC of 3.1 to 6.2 μM against both bacteria. In this case, lipopeptides bearing a hexanoyl chain were, in general, the most active. *E*. *amylovora* was the least sensitive of these bacteria with 24 lipopeptides showing MIC < 12.5 μM from which 17 were active with MIC of 3.1 to 6.2 μM. Against this bacteria, the best peptides bear a hexanoyl or a butanoyl chain.

Concerning the antifungal activity, lipopeptides were considerably more active against *F*. *oxysporum* than against *P*. *expansum*. A MIC < 12.5 μM was observed for 34 and 20 sequences, respectively. Interestingly, against *F*. *oxysporum* 23 lipopeptides showed MIC < 3.1 μM. Among them, 14 peptides displayed MIC of 0.8 to 1.6 μM and 4, MIC of 0.4 to 0.8 μM. In the case of *P*. *expansum*, a MIC < 6.2 μM was obtained for 11 sequences. In general, lipopeptides bearing a hexanoyl chain were the most active against both fungi whereas those incorporating a lauroyl group exhibited the highest MIC values.

### Hemolytic activity

**Fig 2:**
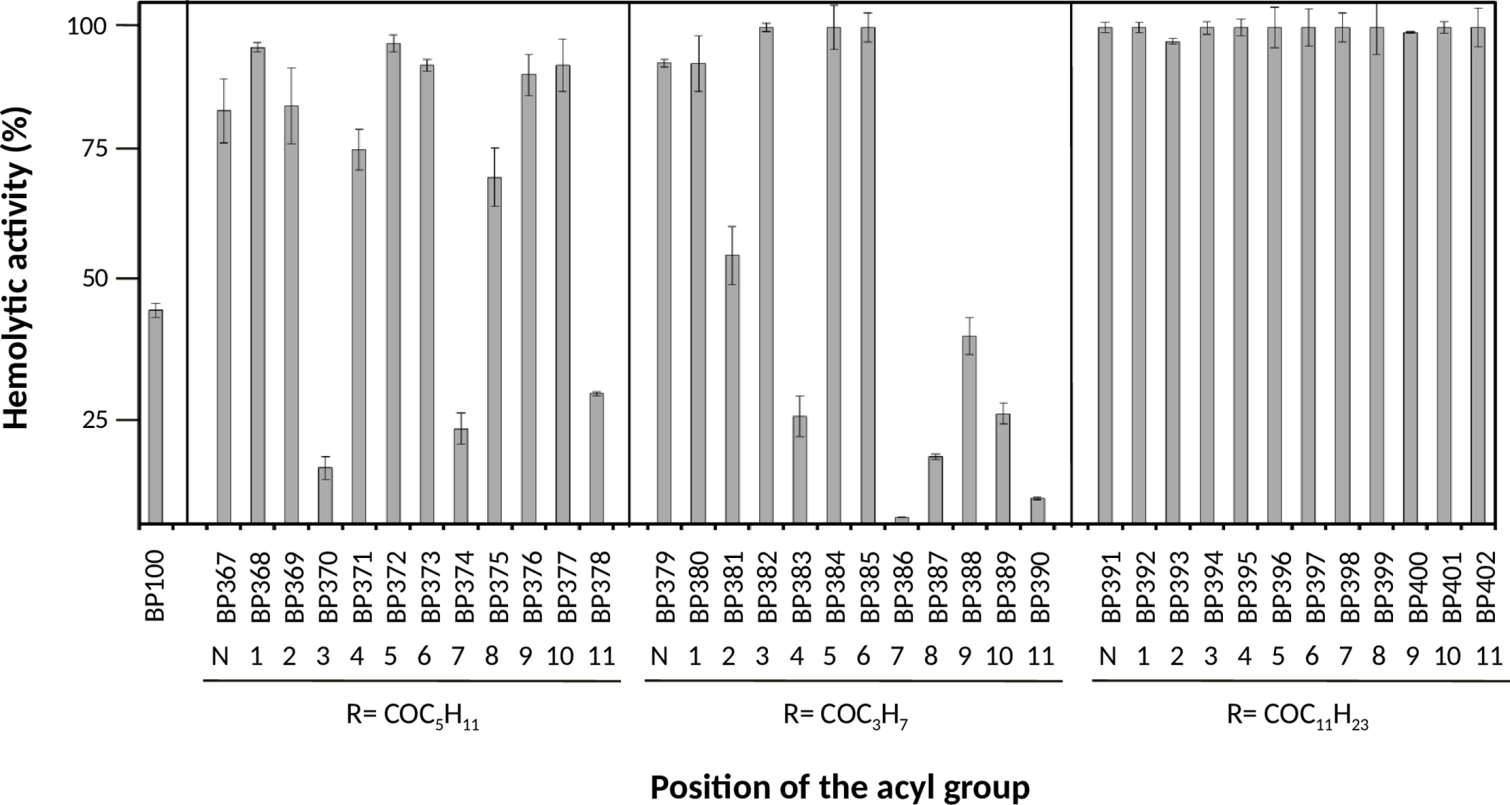
Hemolytic activity of the lipopeptides derived from BP100 at 250 μM. Vertical bars within each column indicate confidence interval at the mean. The position of the acyl group at the N-terminus is indicated by a N, and the position of the acylated Lys by numbers 1 to 11. The type of the acyl group is depicted as R = COC_5_H_11_ (hexanoyl), R = COC_3_H_7_ (butanoyl) and R = COC_11_H_23_ (lauroyl).

The toxicity of antimicrobial peptides targeting the bacterial membrane can be assessed with animal cell model systems, being erythrocytes the most commonly used. Thus, the toxicity of lipopeptides to eukaryotic cells was determined as the ability to lyse erythrocytes in comparison to the reference peptide melittin, a well-known hemolytic peptide. Percent hemolysis at a high concentration of 250 μM is shown in Fig 2 and Table B in S1 File.

Lipopeptides incorporating a lauroyl group were significantly hemolytic, and all derivatives displayed 100% hemolysis at 250 μM. The lipopeptides acylated with a butanoyl group were slightly less hemolytic than those with a hexanoyl group. Among the former derivatives, 6 peptides showed <50% hemolysis at 250 μM.

### Effect on tobacco leaves by infiltration

**Fig 3:**
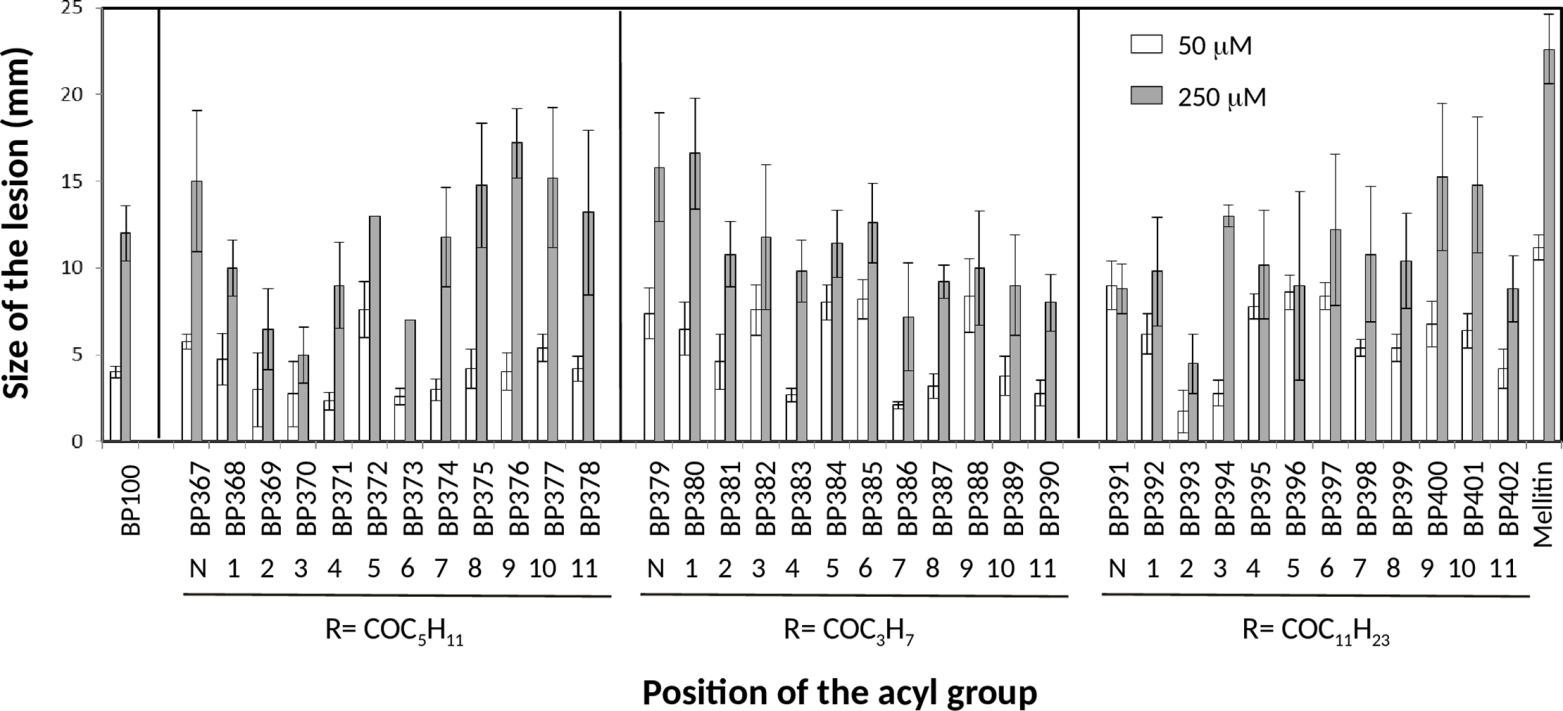
Effect of the lipopeptides derived from BP100 at 50 and 250 μM on the size of the lesions in infiltrated tobacco leaves. This effect was compared to melittin. Vertical bars within each column indicate confidence interval at the mean. The position of the acyl group at the N-terminus is indicated by a N, and the position of the acylated Lys by numbers 1 to 11. The type of the acyl group is depicted as R = COC_5_H_11_ (hexanoyl), R = COC_3_H_7_ (butanoyl) and R = COC_11_H_23_ (lauroyl).

The effect of the 36 lipopeptides in tobacco leaves was assessed by infiltrating 100 μL of a 50, 150 and 250 μM solution of each compound, into the mesophylls of the tobacco plant leaves (Fig 3). **BP100** was also included for comparison purposes, and as a reference control, the nonspecific and nonselective toxic peptide melittin was used. After 48 h of infiltration, a brown necrotic area of 1.1 and 2.3 cm diameter was observed for melittin at 50 and 250 μM, respectively. All lipopeptides had a lower effect than melittin. At 50 μM, all the sequences caused a necrosis ≤ 0.9 cm and for 22 of them the size of the lesions were ≤ 0.6 cm. Sixteen lipopeptides with a necrosis ≤ 1.0 cm at 250 μM were identified. However, for some of the lipopeptides (e.g. **BP378**, **BP400**), in contrast to melittin, the reaction observed was not a typical necrosis in the area of infiltration. Moreover, as expected, an increase of the concentration resulted in an increase of the lesion size.

### Effect of peptide treatment on induction of defense gene expression of tomato plants

A selection of 9 lipopeptides derived from **BP100** were analyzed as potential peptides for inducing the expression of genes related to plant defense responses, such as the salicylic, jasmonic acid and ethylene pathways, and to saline stress and wound damage response (Table 2). The selected lipopeptides display high antimicrobial activity and a different level of hemolysis. Flagellin 15 (**flg15**), jasmonic acid (JA) and acybenzolar-*S*-methyl (ASM) were included in this study as positive controls due to their described capacity to enhance the plant immune system [50].

**Table 2:** Expression of genes related to defense/stress response in tomato after the treatment with the reference products flagellin 15 (flg15), jasmonic acid (JA), and acybenzolar-*S*-methyl (ASM), and with BP100 and 9 selected lipopeptides. Fold induction above 2 is considered overexpression in the relative quantification by the *ΔΔCt* method. Significant values are indicated in bold.

	Reference products^a^				Peptides^b^								
Genes	flg15	JA	ASM	BP100	BP371	BP374	BP378	BP381	BP385	BP387	BP389	BP390	BP400
*Harp*	**3.3**	1.9	**6.3**	0.5	0.9	0.9	1.2	0.7	2.3	1.2	0.9	0.5	1.4
*PR1*	**30.4**	2.9	**4.8**	2	0.7	0.5	2.0	0.2	3.2	0.4	0.3	0.2	0.1
*GluA*	**12.7**	0.2	**5.9**	1.5	0.5	0.5	0.6	0.6	0.7	0.7	0.3	0.6	0.1
*PPO*	**4.3**	**>50**	**2.9**	**3.2**	0.6	0.7	1.7	1.3	1.7	1.3	0.5	0.9	1.4
*LOX*	1.6	**9.7**	**3.3**	1.2	1.1	1.9	1.5	0.8	1.1	0.7	1.2	1.2	1.1
*PinII*	**5.2**	**>50**	0.5	**4.9**	1.7	1.7	**5.8**	0.4	7.1	09	**2.8**	0.2	1.1
*Sub1*	**12.3**	**3.9**	**11.6**	1.8	0.8	0.8	0.9	0.5	1.0	0.8	0.5	0.7	0.5
*ERT3*	**3.6**	**41.3**	**2.9**	1.0	0.9	2.0	1.0	0.7	0.2	0.2	0.1	0.7	0.2
*BCB*	**11.3**	**6.6**	**12.1**	0.8	1.0	1.0	1.3	1.2	**4.2**	1.7	**2.1**	0.6	0.6
*Osm2*	**11.3**	**48.4**	**8.7**	1.0	0.9	1.6	**2.9**	**2.1**	1.2	1.0	1.0	**3.0**	0.9
*Tas14*	**2.3**	0.88	**10.7**	1.4	0.7	1.2	**3.5**	3.4	0.7	1.0	0.7	1.0	**8.3**

^a^ The reference products were tested at 125 μM (flg15), 2.5 mM (JA), and 300 mg/L (ASM).

^b^ Peptides were tested at 125 μM

Table 2 shows the relative quantification for the level of expression of the selected genes. Results showed that positive controls clearly overexpressed the majority of genes, except for *Harp*, *PR1*, *GluA* and *Tas14* for JA; *LOX* for **flg15** and *PinII* for acybenzolar-*S*-methyl. Regarding the peptides, genes *Harp*, *PR1*, *GluA*, *LOX*, *Sub1* and *ERT3* were not overexpressed by any of them. Genes *PinII*, *Osm2*, *BCB* and *Tas14*, related with biotic and abiotic stresses, were overexpressed by 3, 3, 2 and 2 peptides, respectively. In particular, **BP100** overexpressed the *PPO* and *PinII* genes. Among the 9 lipopeptides, none of them caused the overexpression of *PPO*. **BP381**, **BP385**, **BP390**, and **BP400** induced one gene (*Osm2*, *Tas14* or *BCB*), **BP389** overexpressed *PinII* and *BCB*, and treatment with **BP378** induced *PinII*, *Osm2* and *Tas14*.

### Stability to protease degradation

**Fig 4:**
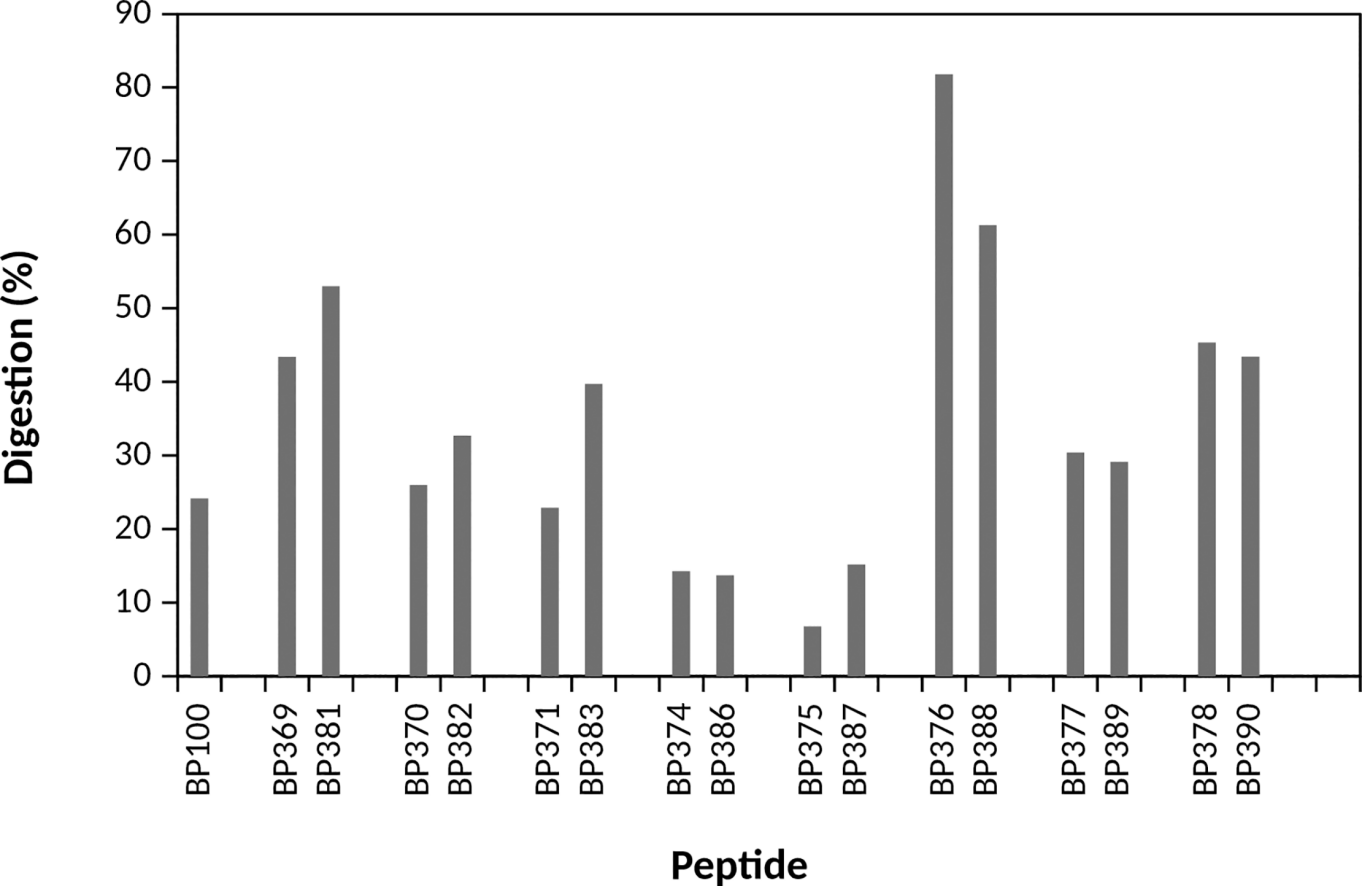
Susceptibility of BP100 and selected lipopeptides to proteinase K hydrolysis. Lipopeptides were treated with proteinase K for 60 min and the percentage of digestion was analyzed by HPLC.

The susceptibility to proteolysis of 16 selected lipopeptides was analyzed after treatment with proteinase K for 60 min and digestion was monitored by reverse-phase HPLC (Fig 4). **BP100** was included in the study for comparison purposes. Among these lipopeptides, 6 sequences displayed a similar stability than **BP100** (23–33% degradation) and 4 were more stable than the parent peptide (7–15% degradation).

## Discussion

Searching for novel antimicrobial agents, safer and more environmetaly friendly than conventional pesticides, for the control of classical and emerging plant diseases, is an important issue for food security and safety. Functional peptides have been proposed as candidates to develop novel fungicides and bactericides for agriculture [8]. Recent studies have shown that the acylation of a peptide is a strategy that can be useful to obtain sequences with improved biological properties [21,26,28–37]. In this regard, we decided to synthesize a group of lipopeptides based on the lead peptide **BP100**.

The length of the hydrophobic chain has been reported to influence the antimicrobial activity of lipopeptides [10,19,21,22,34–37]. It has been shown that, in general, the presence of acyl chains containing 8 to 16 carbons endow peptides with good antimicrobial properties. In contrast to these reports, our studies on cyclic lipopeptides revealed that the incorporation of a long hydrophobic chain prompted a decrease of the activity. In particular, the most active cyclic lipopeptides were those bearing acyl substituents of 4 to 9 carbon atoms. Similarly, in the present work, lipopeptides containing a butanoyl or a hexanoyl chain exhibited the highest activity against all the pathogens tested.

In the present study a different level of sensitivity of bacteria and fungi to lipopeptides was detected which depended on the type of acyl chain. In the case of bacteria, and similarly to previous results, *Xanthomonas* species were the most sensitive to linear undecapeptides whereas *E*. *amylovora* was the least [10,11,42,51]. Moreover, the presence of a lauroyl or a butanoyl chain is preferred for activity against *Xanthomonas* species, hexanoyl-derivatized peptides were the most active against *Pseudomonas* species, and both butanoyl and hexanoyl groups endowed peptides with high activity against *E*. *amylovora*. Regarding fungi, *F*. *oxysporum* was most sensitive than *P*. *expansum*, being the presence of a hexanoyl chain associated with the highest antifungal activity. This distinct susceptibility of bacteria and fungi to a given peptide has been attributed to the different composition of their membranes that could lead to a different peptide binding [10,11,42,51].

The position of the fatty acid chain in the peptide sequence also influenced the antimicrobial activity. Best lipopeptides against *Xanthomonas* species contained the acyl chain at positions 2, 8 or 10, while for *Pseudomonas* species and *E*. *amylovora* the preferred positions were 8 and 11, respectively. In contrast, no clear relationship was observed between the position of the acyl group and the antifungal activity.

The lipopeptide cytotoxicity against red blood cells also depended on the fatty acid length and its position. The incorporation of the 12-carbon atom lauroyl group resulted in the most hemolytic peptides, and butanoyl-derivatized sequences were slightly less hemolytic than the hexanoyl ones. These results are in agreement with previous studies revealing that the incorporation of a long hydrophobic chain, which leads to an increase of the peptide hydrophobicity, is associated with an increase of the hemolysis [10,52,53]. Regarding the position of the acyl chain, in general, lipopeptides containing this acyl group at the N-terminus domain (positions 1 to 6) are more hemolytic than the C-terminal acylated derivatives (positions 7 to 11).

Lipopeptides were less phytotoxic than melittin at concentrations up to 16 to 80 fold higher than the MIC. Other lipopeptides, such as cyclolipopeptides and ultrashort cationic lipopeptides, were also described to be low phytotoxic [10,41]. Moreover, no correlation between the length or the position of the hydrophobic chain and the phytotoxicity was observed. It should be noticed that the lesions observed upon infiltration into the leaf mesophyll might be due to either phytotoxicity or to a programmed cell death like hypersensitivity reaction [47]. The differentiation of both effects would require additional studies.

Compared to the reference compounds flagellin 15, jasmonic acid and acybenzolar-*S*-methyl, the lipopeptides of the present study had a very slight effect of induction of defense-related genes in tomato. Only peptides **BP378** and **BP389** significantly induced the expression of 3 and 2 genes, respectively. Therefore, this result suggests that the incorporation of an acyl group does not affect the overexpression of defense related genes, because these lipopeptides had a similar effect than **BP100**. This fact contrasts with the plant defence elicitation reported for other lipopeptides [54].

Based on the present study, lipopeptides with the best biological activity profile were **BP370**, **BP378**, **BP381**, **BP387** and **BP389** which bear a butanoyl or a hexanoyl chain (Table 3). These peptides were highly active against all the pathogens tested with MIC of 0.8 to 12.5 μM, and were low hemolytic and low phytotoxic. Among them, **BP378** induced the overexpression of three defense-related genes. **BP381** and **BP387** were more active than the parent peptide **BP100** against *X*. *fragariae*, *X*. *axonopodis* pv. *vesicatoria* and *P*. *expansum*, and the latter peptide was more stable than **BP100** (15 vs. 24% degradation). Notably, **BP389** displayed higher activity than **BP100** against the three *Xanthomonas* species and *P*. *expansum* and showed a similar proteolytic stability than **BP100** (29 vs. 24% degradation).

**Table 3:** Lipopeptides with the best biological activity profile.

Biological activity properties	Lipopeptides				
	BP370	BP378	BP381	BP387	BP389
Antibacterial activity (MIC; μM)						
*Ea*^a^	3.1–6.2	3.1–6.2	3.1–6.2	3.1–6.2	3.1–6.2	
*Pss*^a^	3.1–6.2	6.2–12.5	3.1–6.2	3.1–6.2	6.2–12.5	
*Psa*^a^	3.1–6.2	3.1–6.2	3.1–6.2	3.1–6.2	3.1–6.2	
*Xap*^a^	3.1–6.2	3.1–6.2	3.1–6.2	3.1–6.2	0.8–1.6	
*Xf*^a^	6.2–12.5	3.1–6.2	1.6–3.1	1.6–3.1	1.6–3.1	
*Xav*^a^	3.1–6.2	3.1–6.2	1.6–3.1	1.6–3.1	0.8–1.6	
Antifungal activity (MIC; μM)						
*Pe*^a^	6.2–12.5	3.1–6.2	1.6–3.1	6.2–12.5	6.2–12.5	
*Fo*^a^	0.8–1.6	0.8–1.6	0.8–1.6	0.8–1.6	1.6–3.1	
Hemolysis (%)^b^	11 ± 2	26 ± 0.4	54 ± 6	14 ± 0.5	22 ± 2	
Size of the lesions (mm) in infiltrated tobacco leaves^c^	5.0 ± 1.6	13.2 ± 4.7	10.8 ± 1.9	9.2 ± 0.96	9.0 ± 2.9	
Digestion with proteinase K (%)^d^	26	45	53	15	29	
Defense gene expression in tomato plants^e^	nd^f^	*PinII*, *Osm2*, *Tas14*	*Osm2*	none	*PinII*, *BCB*	

^a^ *Ea*, *Erwinia amylovora*; *Pss*, *Pseudomonas syringae* pv. syringae; *Psa*, *Pseudomonas syringae* pv. actinidiae; *Xap*, *Xanthomonas arboricola* pv. pruni; *Xf*, *Xanthomonas fragariae*; *Xav*, *Xanthomonas axonopodis* pv. vesicatoria; *Pe*, *Penicillium expansum*; *Fo*, *Fusarium oxysporum*.

^b^ Percent hemolysis at 250 μM plus confidence interval (α = 0.05).

^c^ Diameter of the lesions (mm) measured after 48 h infiltration of lipopeptides at 250 μM.

^d^ Percentage of degraded lipopeptide after 60 min treatment with proteinase K.

^e^ Expression of genes in tomato plants after the treatment with the lipopeptides at 125 μM.

^f^ nd, not determined.

## Conclusions

The incorporation of an acyl group into the linear undecapeptide **BP100** rendered lipopeptides with an improved biological activity profile. These lipopeptides showed high antimicrobial activity against plant pathogenic bacteria and fungi, displayed different degrees of hemolysis and phytotoxicity, and were significantly stable to protease degradation. Moreover, the acylation did not affect the overexpression of plant defense-related genes in the tomato model plant. Therefore, lipopeptides offer great expectations for developing a wide range of selective functional peptides for plant protection.

## Supporting information

S1 File Table A in S1 File. Antimicrobial activity (MIC) of the linear lipopeptides against six plant pathogenic bacteria and two fungi. Table B in S1 File. Hemolytic activity of the linear lipopeptides. S1 File also includes the synthesis and characterization of the lipopeptides.
(DOCX)
