# Supplementary material for: Antimicrobial activity of linear lipopeptides derived from BP100 towards plant pathogens
Source: PLoS One. 2018 Jul 27;13(7):e0201571. doi: 10.1371/journal.pone.0201571 (PMC6063448; doi:10.1371/journal.pone.0201571)
Supplement: S1 File — Table A in S1 File. Antimicrobial activity (MIC) of the linear lipopeptides against six plant pathogenic bacteria and two fungi. Table B in S1 File. Hemolytic activity of the linear lipopeptides. S1 File also includes the synthesis and characterization of the lipopeptides. (DOCX) [file pone.0201571.s001.docx]

Supporting Information

**Antimicrobial activity of linear lipopeptides derived from BP100 towards plant pathogens**

Àngel Oliveras, Aina Baró, Laura Montesinos, Esther Badosa, Emilio Montesinos, Lidia Feliu*, and Marta Planas*

**Table of contents:**

1. Antimicrobial and hemolytic activity of lipopeptides **BP367-BP402** 2

#### 2. Synthesis of lipopeptides BP367-BP402 5

3. Characterization of lipopeptides **BP367-BP402**: HPLC of crude and purified lipopeptides, ESI-MS and HRMS 16

**1. Antimicrobial and hemolytic activity of lipopeptides BP367-BP402**

**Table A.** Antimicrobial activity (MIC) of the linear lipopeptides against six plant pathogenic bacteria and two fungi

| Peptide | Structure^a^ | *Ea^b^* | *Pss^b^* | *Psa^b^* | *Xap^b^* | *Xf^b^* | *Xav^b^* | *Pe^b^* | *Fo^b^* |
| --- | --- | --- | --- | --- | --- | --- | --- | --- | --- |
| **BP100** | KKLFKKILKYL-NH_2_ | 3.1-6.2 | 3.1-6.2 | 1.6-3.1 | 1.6-3.1 | 3.1-6.2 | 3.1-6.2 | >25 | 0.8-1.6 |
| **BP367** | C_5_H_11_CO-KKLFKKILKYL-NH_2_ | 3.1-6.2 | 3.1-6.2 | 3.1-6.2 | 3.1-6.2 | 3.1-6.2 | 3.1-6.2 | 12.5-25 | 1.6-3.1 |
| **BP368** | Ac-K(COC_5_H_11_)KLFKKILKYL-NH_2_ | 6.2-12.5 | 6.2-12.5 | 6.2-12.5 | 3.1-6.2 | 6.2-12.5 | 3.1-6.2 | 1.6-3.1 | 0.8-1.6 |
| **BP369** | Ac-KK(COC_5_H_11_)LFKKILKYL-NH_2_ | 6.2-12.5 | 6.2-12.5 | 3.1-6.2 | 0.8-1.6 | 3.1-6.2 | 3.1-6.2 | 1.6-3.1 | 0.4-0.8 |
| **BP370** | Ac-KKK(COC_5_H_11_)FKKILKYL-NH_2_ | 3.1-6.2 | 3.1-6.2 | 3.1-6.2 | 3.1-6.2 | 6.2-12.5 | 3.1-6.2 | 6.2-12.5 | 0.8-1.6 |
| **BP371** | Ac-KKLK(COC_5_H_11_)KKILKYL-NH_2_ | 3.1-6.2 | 3.1-6.2 | 3.1-6.2 | 3.1-6.2 | 3.1-6.2 | 3.1-6.2 | 3.1-6.2 | 0.8-1.6 |
| **BP372** | Ac-KKLFK(COC_5_H_11_)KILKYL-NH_2_ | 6.2-12.5 | 6.2-12.5 | 6.2-12.5 | 3.1-6.2 | 3.1-6.2 | 6.2-12.5 | 6.2-12.5 | 0.8-1.6 |
| **BP373** | Ac-KKLFKK(COC_5_H_11_)ILKYL-NH_2_ | 3.1-6.2 | 6.2-12.5 | 3.1-6.2 | 3.1-6.2 | 3.1-6.2 | 6.2-12.5 | 3.1-6.2 | 0.4-0.8 |
| **BP374** | Ac-KKLFKKK(COC_5_H_11_)LKYL-NH_2_ | 3.1-6.2 | 6.2-12.5 | 3.1-6.2 | 6.2-12.5 | 3.1-6.2 | 6.2-12.5 | >25 | 0.8-1.6 |
| **BP375** | Ac-KKLFKKIK(COC_5_H_11_)KYL-NH_2_ | 3.1-6.2 | 3.1-6.2 | 3.1-6.2 | 3.1-6.2 | 3.1-6.2 | 6.2-12.5 | 3.1-6.2 | 0.4-0.8 |
| **BP376** | Ac-KKLFKKILK(COC_5_H_11_)YL-NH_2_ | 6.2-12.5 | 12.5-25 | 3.1-6.2 | 6.2-12.5 | 3.1-6.2 | 6.2-12.5 | 3.1-6.2 | 0.4-0.8 |
| **BP377** | Ac-KKLFKKILKK(COC_5_H_11_)L-NH_2_ | 3.1-6.2 | 3.1-6.2 | 3.1-6.2 | 3.1-6.2 | 3.1-6.2 | 6.2-12.5 | 3.1-6.2 | 0.8-1.6 |
| **BP378** | Ac-KKLFKKILKYK(COC_5_H_11_)-NH_2_ | 3.1-6.2 | 6.2-12.5 | 3.1-6.2 | 3.1-6.2 | 3.1-6.2 | 3.1-6.2 | 3.1-6.2 | 0.8-1.6 |
| **BP379** | C_3_H_7_CO-KKLFKKILKYL-NH_2_ | 3.1-6.2 | 3.1-6.2 | 3.1-6.2 | 3.1-6.2 | 3.1-6.2 | 3.1-6.2 | 6.2-12.5 | 3.1-6.2 |
| **BP380** | Ac-K(COC_3_H_7_)KLFKKILKYL-NH_2_ | 3.1-6.2 | 6.2-12.5 | 6.2-12.5 | 3.1-6.2 | 3.1-6.2 | 3.1-6.2 | 3.1-6.2 | 0.8-1.6 |
| **BP381** | Ac-KK(COC_3_H_7_)LFKKILKYL-NH_2_ | 3.1-6.2 | 3.1-6.2 | 3.1-6.2 | 3.1-6.2 | 1.6-3.1 | 1.6-3.1 | 1.6-3.1 | 0.8-1.6 |
| **BP382** | Ac-KKK(COC_3_H_7_)FKKILKYL-NH_2_ | 6.2-12.5 | 6.2-12.5 | 6.2-12.5 | 6.2-12.5 | 1.6-3.1 | 6.2-12.5 | 3.1-6.2 | 0.8-1.6 |
| **BP383** | Ac-KKLK(COC_3_H_7_)KKILKYL-NH_2_ | 6.2-12.5 | 6.2-12.5 | 6.2-12.5 | 6.2-12.5 | 3.1-6.2 | 3.1-6.2 | 6.2-12.5 | 0.8-1.6 |
| **BP384** | Ac-KKLFK(COC_3_H_7_)KILKYL-NH_2_ | 3.1-6.2 | 6.2-12.5 | 6.2-12.5 | 3.1-6.2 | 1.6-3.1 | 1.6-3.1 | 12.5-25 | 1.6-3.1 |
| **BP385** | Ac-KKLFKK(COC_3_H_7_)ILKYL-NH_2_ | 6.2-12.5 | 6.2-12.5 | 6.2-12.5 | 0.8-1.6 | 1.6-3.1 | 0.8-1.6 | 6.2-12.5 | 1.6-3.1 |
| **BP386** | Ac-KKLFKKK(COC_3_H_7_)LKYL-NH_2_ | 12.5-25 | 6.5-12.5 | 6.2-12.5 | 12.5-25 | 12.5-25 | 12.5-25 | >25 | 12.5-25 |
| **BP387** | Ac-KKLFKKIK(COC_3_H_7_)KYL-NH_2_ | 3.1-6.2 | 3.1-6.2 | 3.1-6.2 | 3.1 -6.2 | 1.6-3.1 | 1.6-3.1 | 6.2-12.5 | 0.8-1.6 |
| **BP388** | Ac-KKLFKKILK(COC_3_H_7_)YL-NH_2_ | 3.1-6.2 | 6.2-12.5 | 6.2-12.5 | 3.1-6.2 | 1.6-3.1 | 1.6-3.1 | 6.2-12.5 | 0.8-1.6 |
| **BP389** | Ac-KKLFKKILKK(COC_3_H_7_)L-NH_2_ | 3.1-6.2 | 6.2-12.5 | 3.1-6.2 | 0.8-1.6 | 1.6-3.1 | 0.8-1.6 | 6.2-12.5 | 1.6-3.1 |
| **BP390** | Ac-KKLFKKILKYK(COC_3_H_7_)-NH_2_ | 3.1-6.2 | 6.2 -12.5 | 6.2-12.5 | 6.2-12.5 | 3.1-6.2 | 3.1-6.2 | 6.2-12.5 | 0.8-1.6 |

| **BP391** | C_11_H_23_CO-KKLFKKILKYL-NH_2_ | >25 | >25 | >25 | 6.2-12.5 | 3.1-6.2 | 6.2-12.5 | >25 | 12.5-25 |
| --- | --- | --- | --- | --- | --- | --- | --- | --- | --- |
| **BP392** | Ac-K(COC_11_H_23_)KLFKKILKYL-NH_2_ | >25 | >25 | >25 | 3.1-6.2 | 0.8-1.6 | 3.1-6.2 | >25 | 6.2-12.5 |
| **BP393** | Ac-KK(COC_11_H_23_)LFKKILKYL-NH_2_ | 12.5-25 | 12.5-25 | 6.2-12.5 | 3.1-6.2 | 3.1-6.2 | 3.1-6.2 | >25 | 1.6-3.1 |
| **BP394** | Ac-KKK(COC_11_H_23_)FKKILKYL-NH_2_ | 12.5 -25 | 6.2-12.5 | 6.2-12.5 | 1.6-3.1 | 0.8-1.6 | 1.6-3.1 | 12.5-25 | 6.2-12.5 |
| **BP395** | Ac-KKLK(COC_11_H_23_)KKILKYL-NH_2_ | 12.5 -25 | 3.1-6.2 | 3.1-6.2 | 1.6-3.1 | 1.6-3.1 | 1.6-3.1 | 12.5-25 | 6.2-12.5 |
| **BP396** | Ac-KKLFK(COC_11_H_23_)KILKYL-NH_2_ | >25 | >25 | >25 | 1.6-3.1 | 1.6-3.1 | 3.1-6.2 | 12.5-25 | 6.2-12.5 |
| **BP397** | Ac-KKLFKK(COC_11_H_23_)ILKYL-NH_2_ | >25 | >25 | >25 | 3.1-6.2 | 1.6-3.1 | 6.2-12.5 | 12.5-25 | 6.2-12.5 |
| **BP398** | Ac-KKLFKKK(COC_11_H_23_)LKYL-NH_2_ | >25 | 3.1-6.2 | 3.1-6.2 | 1.6-3.1 | 3.1-6.2 | 0.8-1.6 | >25 | 6.2-12.5 |
| **BP399** | Ac-KKLFKKIK(COC_11_H_23_)KYL-NH_2_ | >25 | 3.1-6.2 | 3.1-6.2 | 1.6-3.1 | 3.1-6.2 | 0.8-1.6 | 12.5-25 | 6.2-12.5 |
| **BP400** | Ac-KKLFKKILK(COC_11_H_23_)YL-NH_2_ | >25 | >25 | >25 | 1.6-3.1 | 3.1-6.2 | 1.6-3.1 | >25 | 6.2-12.5 |
| **BP401** | Ac-KKLFKKILKK(COC_11_H_23_)L-NH_2_ | >25 | 6.2-12.5 | 6.2-12.5 | 1.6-3.1 | 3.1-6.2 | 0.8-1.6 | 12.5-25 | 6.2-12.5 |
| **BP402** | Ac-KKLFKKILKYK(COC_11_H_23_)-NH_2_ | 3.1-6.2 | 3.1-6.2 | 3.1-6.2 | 1.6-3.1 | 3.1-6.2 | 1.6-3.1 | 12.5-25 | 6.2-12.5 |

^a^COC_5_H_11_, hexanoyl; COC_3_H_7_, butanoyl; COC_11_H_23_, lauroyl.

^b^*Ea*, *Erwinia amylovora*; *Pss*, *Pseudomonas syringae* pv. syringae; *Psa*, *Pseudomonas syringae* pv. actinidiae; *Xap*, *Xanthomonas arboricola* pv. pruni; *Xf*, *Xanthomonas fragariae*; *Xav*, *Xanthomonas axonopodis* pv. vesicatoria; *Pe*, *Penicillium expansum*; *Fo*, *Fusarium oxysporum*.

**Table B.** Hemolytic activity of the linear lipopeptides

| Peptide | Structure^a^ | 250 μM | 375 μM |
| --- | --- | --- | --- |
| **BP100** | KKLFKKILKYL-NH_2_ | 43 ± 1.4 | 57 ± 4.6 |
| **BP367** | C_5_H_11_CO-KKLFKKILKYL-NH_2_ | 83 ± 6 | 95 ± 13 |
| **BP368** | Ac-K(COC_5_H_11_)KLFKKILKYL-NH_2_ | 96 ± 0.9 | 97 ± 3 |
| **BP369** | Ac-KK(COC_5_H_11_)LFKKILKYL-NH_2_ | 84 ± 8 | 86 ± 8 |
| **BP370** | Ac-KKK(COC_5_H_11_)FKKILKYL-NH_2_ | 11 ± 2 | 14 ± 3 |
| **BP371** | Ac-KKLK(COC_5_H_11_)KKILKYL-NH_2_ | 75 ± 4 | 95 ± 3 |
| **BP372** | Ac-KKLFK(COC_5_H_11_)KILKYL-NH_2_ | 97 ± 2 | 100 ± 0.9 |
| **BP373** | Ac-KKLFKK(COC_5_H_11_)ILKYL-NH_2_ | 92 ± 1 | 95 ± 6 |
| **BP374** | Ac-KKLFKKK(COC_5_H_11_)LKYL-NH_2_ | 19 ± 3 | 28 ± 2 |
| **BP375** | Ac-KKLFKKIK(COC_5_H_11_)KYL-NH_2_ | 70 ± 6 | 86 ± 3 |
| **BP376** | Ac-KKLFKKILK(COC_5_H_11_)YL-NH_2_ | 90 ± 4 | 89 ± 2 |
| **BP377** | Ac-KKLFKKILKK(COC_5_H_11_)L-NH_2_ | 92 ± 5 | 89 ± 2 |
| **BP378** | Ac-KKLFKKILKYK(COC_5_H_11_)-NH_2_ | 26 ± 0.4 | 52 ± 6 |
| **BP379** | C_3_H_7_CO-KKLFKKILKYL-NH_2_ | 93 ± 0.8 | 96 ± 1 |
| **BP380** | Ac-K(COC_3_H_7_)KLFKKILKYL-NH_2_ | 93 ± 6 | 97 ± 5 |
| **BP381** | Ac-KK(COC_3_H_7_)LFKKILKYL-NH_2_ | 54 ± 6 | 76 ± 2 |
| **BP382** | Ac-KKK(COC_3_H_7_)FKKILKYL-NH_2_ | 100 ± 0.9 | 100 ± 2 |
| **BP383** | Ac-KKLK(COC_3_H_7_)KKILKYL-NH_2_ | 22 ± 4 | 40 ± 4 |
| **BP384** | Ac-KKLFK(COC_3_H_7_)KILKYL-NH_2_ | 100 ± 4 | 100 ± 2 |
| **BP385** | Ac-KKLFKK(COC_3_H_7_)ILKYL-NH_2_ | 100 ± 3 | 100 ± 4 |
| **BP386** | Ac-KKLFKKK(COC_3_H_7_)LKYL-NH_2_ | 1 ± 0.1 | 3 ± 0 |
| **BP387** | Ac-KKLFKKIK(COC_3_H_7_)KYL-NH_2_ | 14 ± 0.5 | 18 ± 1 |
| **BP388** | Ac-KKLFKKILK(COC_3_H_7_)YL-NH_2_ | 38 ± 4 | 89 ± 10 |
| **BP389** | Ac-KKLFKKILKK(COC_3_H_7_)L-NH_2_ | 22 ± 2 | 39 ± 3 |
| **BP390** | Ac-KKLFKKILKYK(COC_3_H_7_)-NH_2_ | 5 ± 0.3 | 7 ± 0.9 |
| **BP391** | C_11_H_23_CO-KKLFKKILKYL-NH_2_ | 100 ± 0.9 | 100 ± 0.5 |
| **BP392** | Ac-K(COC_11_H_23_)KLFKKILKYL-NH_2_ | 100 ± 0.9 | 100 ± 2 |
| **BP393** | Ac-KK(COC_11_H_23_)LFKKILKYL-NH_2_ | 97 ± 0.6 | 99 ± 0.5 |
| **BP394** | Ac-KKK(COC_11_H_23_)FKKILKYL-NH_2_ | 100 ± 1 | 100 ± 2 |
| **BP395** | Ac-KKLK(COC_11_H_23_)KKILKYL-NH_2_ | 100 ± 2 | 100 ± 2 |
| **BP396** | Ac-KKLFK(COC_11_H_23_)KILKYL-NH_2_ | 100 ± 4 | 100 ± 7 |
| **BP397** | Ac-KKLFKK(COC_11_H_23_)ILKYL-NH_2_ | 100 ± 4 | 100 ± 5 |
| **BP398** | Ac-KKLFKKK(COC_11_H_23_)LKYL-NH_2_ | 100 ± 3 | 100 ± 5 |
| **BP399** | Ac-KKLFKKIK(COC_11_H_23_)KYL-NH_2_ | 100 ± 5 | 100 ± 6 |
| **BP400** | Ac-KKLFKKILK(COC_11_H_23_)YL-NH_2_ | 99 ± 0.4 | 100 ± 14 |
| **BP401** | Ac-KKLFKKILKK(COC_11_H_23_)L-NH_2_ | 100 ± 1 | 100 ± 3 |
| **BP402** | Ac-KKLFKKILKYK(COC_11_H_23_)-NH_2_ | 100 ± 4 | 100 ± 0.5 |

^a^COC_5_H_11_, hexanoyl; COC_3_H_7_, butanoyl; COC_11_H_23_, lauroyl.

^b^Percent hemolysis at 250 and 375 μM plus confidence interval (α = 0.05)

#### 2. Synthesis of lipopeptides BP367-BP402

#### C_5_H_11_CO-Lys-Lys-Leu-Phe-Lys-Lys-Ile-Leu-Lys-Tyr-Leu-NH_2_ (BP367)

This lipopeptide was prepared following the procedure described in the manuscript using hexanoic acid. Acidolytic cleavage of the resulting resin and purification eluting with H_2_O/CH_3_CN (70:30) afforded C_5_H_11_CO-Lys-Lys-Leu-Phe-Lys-Lys-Ile-Leu-Lys-Tyr-Leu-NH_2_ (**BP367**) in >99% purity. *t*_R_ = 7.77 min. MS (ESI) *m/z*: 760.1 [M + 2H]^2+^, 1520.1 [M + H]^+^; HRMS (ESI) *m/z*: calcd for C_78_H_137_N_17_O_13_ [M + 2H]^2+^ 760.0285, found 760.0270; calcd for C_78_H_138_N_17_O_13_ [M + 3H]^3+^ 507.0215, found 507.0201; calcd for C_78_H_139_N_17_O_13_ [M + 4H]^4+^ 380.5179, found 380.5165.

#### Ac-Lys(COC_5_H_11_)-Lys-Leu-Phe-Lys-Lys-Ile-Leu-Lys-Tyr-Leu-NH_2_ (BP368)

This lipopeptide was prepared following the procedure described in the manuscript using hexanoic acid. Acidolytic cleavage of the resulting resin and purification eluting with H_2_O/CH_3_CN (70:30) afforded Ac-Lys(COC_5_H_11_)-Lys-Leu-Phe-Lys-Lys-Ile-Leu-Lys-Tyr-Leu-NH_2_ (**BP368**) in >99% purity. *t*_R_ = 7.91 min. MS (ESI) *m/z*: 521.3 [M + 3H]^3+^, 781.6 [M + 2H]^2+^, 1562.1 [M + H]^+^; HRMS (ESI) *m/z*: calcd for C_80_H_139_N_17_O_14_ [M + 2H]^2+^ 781.0338, found 781.0319; calcd for C_80_H_140_N_17_O_14_ [M + 3H]^3+^ 521.0250, found 521.0252.

#### Ac-Lys-Lys(COC_5_H_11_)-Leu-Phe-Lys-Lys-Ile-Leu-Lys-Tyr-Leu-NH_2_ (BP369)

This lipopeptide was prepared following the procedure described in the manuscript using hexanoic acid. Acidolytic cleavage of the resulting resin and purification eluting with H_2_O/CH_3_CN (75:25) afforded Ac-Lys-Lys(COC_5_H_11_)-Leu-Phe-Lys-Lys-Ile-Leu-Lys-Tyr-Leu-NH_2_ (**BP369**) in 92% purity. *t*_R_ = 7.53 min. MS (ESI) *m/z*: 521.4 [M + 3H]^3+^, 781.1 [M + 2H]^2+^, 1562.1 [M + H]^+^; HRMS (ESI) *m/z*: calcd for C_80_H_139_N_17_O_14_ [M + 2H]^2+^ 781.0338, found 781.0316; calcd for C_80_H_140_N_17_O_14_ [M + 3H]^3+^ 521.0250, found 521.0263; calcd for C_80_H_141_N_17_O_14_ [M + 4H]^4+^ 391.0205, found 391.0192.

#### Ac-Lys-Lys-Lys(COC_5_H_11_)-Phe-Lys-Lys-Ile-Leu-Lys-Tyr-Leu-NH_2_ (BP370)

This lipopeptide was prepared following the procedure described in the manuscript using hexanoic acid. Acidolytic cleavage of the resulting resin and purification eluting with H_2_O/CH_3_CN (80:20) afforded Ac-Lys-Lys-Lys(COC_5_H_11_)-Phe-Lys-Lys-Ile-Leu-Lys-Tyr-Leu-NH_2_ (**BP370**) in >99% purity. *t*_R_ = 7.06 min. MS (ESI) *m/z*: 526.1 [M + 3H]^3+^, 789.1 [M + 2H]^2+^, 1577.1 [M + H]^+^; HRMS (ESI) *m/z*: calcd for C_80_H_140_N_18_O_14_ [M + 2H]^2+^ 788.5393, found 788.5380; calcd for C_80_H_141_N_18_O_14_ [M + 3H]^3+^ 526.0286, found 526.0275; calcd for C_80_H_142_N_18_O_14_ [M + 4H]^4+^ 394.7733, found 394.7725.

#### Ac-Lys-Lys-Leu-Lys(COC_5_H_11_)-Lys-Lys-Ile-Leu-Lys-Tyr-Leu-NH_2_ (BP371)

This lipopeptide was prepared following the procedure described in the manuscript using hexanoic acid. Acidolytic cleavage of the resulting resin and purification eluting with H_2_O/CH_3_CN (80:20) afforded Ac-Lys-Lys-Leu-Lys(COC_5_H_11_)-Lys-Lys-Ile-Leu-Lys-Tyr-Leu-NH_2_ (**BP371**) in >99% purity. *t*_R_ = 7.11 min. MS (ESI) *m/z*: 771.6 [M + 2H]^2+^, 1542.2 [M + H]^+^; HRMS (ESI) *m/z*: calcd for C_77_H_142_N_18_O_14_ [M + 2H]^2+^ 771.5471, found 771.5471; calcd for C_77_H_143_N_18_O_14_ [M + 3H]^3+^ 514.7005, found 514.7003; calcd for C_77_H_144_N_18_O_14_ [M + 4H]^4+^ 386.2772, found 386.2768.

#### Ac-Lys-Lys-Leu-Phe-Lys(COC_5_H_11_)-Lys-Ile-Leu-Lys-Tyr-Leu-NH_2_ (BP372)

This lipopeptide was prepared following the procedure described in the manuscript using hexanoic acid. Acidolytic cleavage of the resulting resin and purification eluting with H_2_O/CH_3_CN (70:30) afforded Ac-Lys-Lys-Leu-Phe-Lys(COC_5_H_11_)-Lys-Ile-Leu-Lys-Tyr-Leu-NH_2_ (**BP372**) in >99% purity. *t*_R_ = 7.64 min. MS (ESI) *m/z*: 781.1 [M + 2H]^2+^, 1562.1 [M + H]^+^; HRMS (ESI) *m/z*: calcd for C_80_H_139_N_17_O_14_ [M + 2H]^2+^ 781.0338, found 781.0336; calcd for C_80_H_140_N_17_O_14_ [M + 3H]^3+^ 521.0250, found 521.0247; calcd for C_80_H_141_N_17_O_14_ [M + 4H]^4+^ 391.0205, found 391.0199.

#### Ac-Lys-Lys-Leu-Phe-Lys-Lys(COC_5_H_11_)-Ile-Leu-Lys-Tyr-Leu-NH_2_ (BP373)

This lipopeptide was prepared following the procedure described in the manuscript using hexanoic acid. Acidolytic cleavage of the resulting resin and purification eluting with H_2_O/CH_3_CN (80:20) afforded Ac-Lys-Lys-Leu-Phe-Lys-Lys(COC_5_H_11_)-Ile-Leu-Lys-Tyr-Leu-NH_2_ (**BP373**) in >99% purity. *t*_R_ = 7.94 min. MS (ESI) *m/z*: 781.6 [M + 2H]^2+^, 1561.1 [M + H]^+^, 1583.1 [M + Na]^+^; HRMS (ESI) *m/z*: calcd for C_80_H_139_N_17_O_14_ [M + 2H]^2+^ 781.0338, found 781.0330; calcd for C_80_H_140_N_17_O_14_ [M + 3H]^3+^ 521.0250, found 521.0244.

#### Ac-Lys-Lys-Leu-Phe-Lys-Lys-Lys(COC_5_H_11_)-Leu-Lys-Tyr-Leu-NH_2_ (BP374)

This lipopeptide was prepared following the procedure described in the manuscript using hexanoic acid. Acidolytic cleavage of the resulting resin and purification eluting with H_2_O/CH_3_CN (80:20) afforded Ac-Lys-Lys-Leu-Phe-Lys-Lys-Lys(COC_5_H_11_)-Leu-Lys-Tyr-Leu-NH_2_ (**BP374**) in >99% purity. *t*_R_ = 6.91 min. MS (ESI) *m/z*: 789.1 [M + 2H]^2+^, 1577.1 [M + H]^+^, 1598.1 [M + Na]^+^; HRMS (ESI) *m/z*: calcd for C_80_H_140_N_18_O_14_ [M + 2H]^2+^ 788.5393, found 788.5393; calcd for C_80_H_141_N_18_O_14_ [M + 3H]^3+^ 526.0286, found 526.0284; calcd for C_80_H_142_N_18_O_14_ [M + 4H]^4+^ 394.7733, found 394.7733.

#### Ac -Lys-Lys-Leu-Phe-Lys-Lys-Ile-Lys(COC_5_H_11_)-Lys-Tyr-Leu-NH_2_ (BP375)

This lipopeptide was prepared following the procedure described in the manuscript using hexanoic acid. Acidolytic cleavage of the resulting resin and purification eluting with H_2_O/CH_3_CN (75:25) afforded Ac-Lys-Lys-Leu-Phe-Lys-Lys-Ile-Lys(COC_5_H_11_)-Lys-Tyr-Leu-NH_2_ (**BP375**) in >99% purity. *t*_R_ = 7.17 min. MS (ESI) *m/z*: 789.1 [M + 2H]^2+^, 1577.1 [M + H]^+^, 1599.1 [M + Na]^+^; HRMS (ESI) *m/z*: calcd for C_80_H_140_N_18_O_14_ [M + 2H]^2+^ 788.5393, found 788.5383; calcd for C_80_H_141_N_18_O_14_ [M + 3H]^3+^ 526.0286, found 526.0276; calcd for C_80_H_142_N_18_O_14_ [M + 4H]^4+^ 394.7733, found 394.7729.

#### Ac-Lys-Lys-Leu-Phe-Lys-Lys-Ile-Leu-Lys(COC_5_H_11_)-Tyr-Leu-NH_2_ (BP376)

This lipopeptide was prepared following the procedure described in the manuscript using hexanoic acid. Acidolytic cleavage of the resulting resin and purification eluting with H_2_O/CH_3_CN (70:30) afforded Ac-Lys-Lys-Leu-Phe-Lys-Lys-Ile-Leu-Lys(COC_5_H_11_)-Tyr-Leu-NH_2_ (**BP376**) in >99% purity. *t*_R_ = 7.48 min. MS (ESI) *m/z*: 781.6 [M + 2H]^2+^, 1561.1 [M + H]^+^, 1583.1 [M + Na]^+^; HRMS (ESI) *m/z*: calcd for C_80_H_139_N_17_O_14_ [M + 2H]^2+^ 781.0338, found 781.0311; calcd for C_80_H_140_N_17_O_14_ [M + 3H]^3+^ 521.0250, found 521.0233.

#### Ac-Lys-Lys-Leu-Phe-Lys-Lys-Ile-Leu-Lys-Lys(COC_5_H_11_)-Leu-NH_2_ (BP377)

This lipopeptide was prepared following the procedure described in the manuscript using hexanoic acid. Acidolytic cleavage of the resulting resin and purification eluting with H_2_O/CH_3_CN (75:25) afforded Ac-Lys-Lys-Leu-Phe-Lys-Lys-Ile-Leu-Lys-Lys(COC_5_H_11_)-Leu-NH_2_ (**BP377**) in >99% purity. *t*_R_ = 7.58 min. MS (ESI) *m/z*: 763.6 [M + 2H]^2+^, 1526.2 [M + H]^+^; HRMS (ESI) *m/z*: calcd for C_77_H_142_N_18_O_13_ [M + 2H]^2+^ 763.5496, found 763.5486; calcd for C_77_H_143_N_18_O_13_ [M + 3H]^3+^ 509.3689, found 509.3700; calcd for C_77_H_144_N_18_O_13_ [M + 4H]^4+^ 382.2785, found 382.2791.

#### Ac-Lys-Lys-Leu-Phe-Lys-Lys-Ile-Leu-Lys-Tyr-Lys(COC_5_H_11_)-NH_2_ (BP378)

This lipopeptide was prepared following the procedure described in the manuscript using hexanoic acid. Acidolytic cleavage of the resulting resin and purification eluting with H_2_O/CH_3_CN (80:20) afforded Ac-Lys-Lys-Leu-Phe-Lys-Lys-Ile-Leu-Lys-Tyr-Lys(COC_5_H_11_)-NH_2_ (**BP378**) in >99% purity. *t*_R_ = 6.96 min. MS (ESI) *m/z*: 788.6 [M + 2H]^2+^, 1577.1 [M + H]^+^; HRMS (ESI) *m/z*: calcd for C_80_H_140_N_18_O_14_ [M + 2H]^2+^ 788.5393, found 788.5396; calcd for C_80_H_141_N_18_O_14_ [M + 3H]^3+^ 526.0286, found 526.0308; calcd for C_80_H_142_N_18_O_14_ [M + 4H]^4+^ 394.7733, found 394.7742.

#### C_3_H_7_CO-Lys-Lys-Leu-Phe-Lys-Lys-Ile-Leu-Lys-Tyr-Leu-NH_2_ (BP379)

This lipopeptide was prepared following the procedure described in the manuscript using butyric acid. Acidolytic cleavage of the resulting resin and purification eluting with H_2_O/CH_3_CN (75:25) afforded C_3_H_7_CO-Lys-Lys-Leu-Phe-Lys-Lys-Ile-Leu-Lys-Tyr-Leu-NH_2_ (**BP379**) in >99% purity. *t*_R_ = 7.52 min. MS (ESI) *m/z*: 746.0 [M + 2H]^2+^, 1491.1 [M + H]^+^, 1513.1 [M + Na]^+^; HRMS (ESI) *m/z*: calcd for C_76_H_133_N_17_O_13_ [M + 2H]^2+^ 746.0129, found 746.0102; calcd for C_76_H_134_N_17_O_13_ [M + 3H]^3+^ 497.6777, found 497.6770; calcd for C_76_H_135_N_17_O_13_ [M + 4H]^4+^ 373.5101, found 373.5104.

#### Ac-Lys(COC_3_H_7_)-Lys-Leu-Phe-Lys-Lys-Ile-Leu-Lys-Tyr-Leu-NH_2_ (BP380)

This lipopeptide was prepared following the procedure described in the manuscript using butyric acid. Acidolytic cleavage of the resulting resin and purification eluting with H_2_O/CH_3_CN (70:30) afforded Ac-Lys(COC_3_H_7_)-Lys-Leu-Phe-Lys-Lys-Ile-Leu-Lys-Tyr-Leu-NH_2_ (**BP380**) in >99% purity. *t*_R_ = 7.79 min. MS (ESI) *m/z*: 1533.3 [M + H]^+^, 1555.3 [M + Na]^+^; HRMS (ESI) *m/z*: calcd for C_78_H_135_N_17_O_14_ [M + 2H]^2+^ 767.0182, found 767.0158; calcd for C_78_H_136_N_17_O_14_ [M + 3H]^3+^ 511.6812, found 511.6805; calcd for C_78_H_137_N_17_O_14_ [M + 4H]^4+^ 384.0127, found 384.0117.

#### Ac-Lys-Lys(COC_3_H_7_)-Leu-Phe-Lys-Lys-Ile-Leu-Lys-Tyr-Leu-NH_2_ (BP381)

This lipopeptide was prepared following the procedure described in the manuscript using butyric acid. Acidolytic cleavage of the resulting resin and purification eluting with H_2_O/CH_3_CN (80:20) afforded Ac-Lys-Lys(COC_3_H_7_)-Leu-Phe-Lys-Lys-Ile-Leu-Lys-Tyr-Leu-NH_2_ (**BP381**) in >99% purity. *t*_R_ = 6.96 min. MS (ESI) *m/z*: 511.9 [M + 3H]^3+^, 767.5 [M + 2H]^2+^, 1533.1 [M + H]^+^, 1555.3 [M + Na]^+^; HRMS (ESI) *m/z*: calcd for C_78_H_135_N_17_O_14_ [M + 2H]^2+^ 767.0182, found 767.0160; calcd for C_78_H_136_N_17_O_14_ [M + 3H]^3+^ 511.6812, found 511.6803; calcd for C_78_H_137_N_17_O_14_ [M + 4H]^4+^ 384.0127, found 384.0119.

#### Ac-Lys-Lys-Lys(COC_3_H_7_)-Phe-Lys-Lys-Ile-Leu-Lys-Tyr-Leu-NH_2_ (BP382)

This lipopeptide was prepared following the procedure described in the manuscript using butyric acid. Acidolytic cleavage of the resulting resin and purification eluting with H_2_O/CH_3_CN (80:20) afforded Ac-Lys-Lys-Lys(COC_3_H_7_)-Phe-Lys-Lys-Ile-Leu-Lys-Tyr-Leu-NH_2_ (**BP382**) in >99% purity. *t*_R_ = 6.91 min. MS (ESI) *m/z*: 1548.2 [M + H]^+^; HRMS (ESI) *m/z*: calcd for C_80_H_136_N_18_O_14_ [M + 2H]^2+^ 774.5236, found 774.5220; calcd for C_78_H_137_N_18_O_14_ [M + 3H]^3+^ 516.6848, found 516.6845; calcd for C_78_H_138_N_18_O_14_ [M + 4H]^4+^ 387.7654, found 387.7648.

#### Ac-Lys-Lys-Leu-Lys(COC_3_H_7_)-Lys-Lys-Ile-Leu-Lys-Tyr-Leu-NH_2_ (BP383)

This lipopeptide was prepared following the procedure described in the manuscript using butyric acid. Acidolytic cleavage of the resulting resin and purification eluting with H_2_O/CH_3_CN (80:20) afforded Ac-Lys-Lys-Leu-Lys(COC_3_H_7_)-Lys-Lys-Ile-Leu-Lys-Tyr-Leu-NH_2_ (**BP383**) in >99% purity. *t*_R_ = 6.41 min. MS (ESI) *m/z*: 1514.2 [M + H]^+^, 1536.1 [M + Na]^+^; HRMS (ESI) *m/z*: calcd for C_75_H_137_N_18_O_14_ [M + H]^+^ 1514.0556, found 1514.0535; calcd for C_75_H_138_N_17_O_14_ [M + 2H]^2+^ 757.5314, found 757.5312.

#### Ac-Lys-Lys-Leu-Phe-Lys(COC_3_H_7_)-Lys-Ile-Leu-Lys-Tyr-Leu-NH_2_ (BP384)

This lipopeptide was prepared following the procedure described in the manuscript using butyric acid. Acidolytic cleavage of the resulting resin and purification eluting with H_2_O/CH_3_CN (75:25) afforded Ac-Lys-Lys-Leu-Phe-Lys(COC_3_H_7_)-Lys-Ile-Leu-Lys-Tyr-Leu-NH_2_ (**BP384**) in >99% purity. *t*_R_ = 6.91 min. MS (ESI) *m/z*: 1533.2 [M + H]^+^, 1555.2 [M + Na]^+^; HRMS (ESI) *m/z*: calcd for C_78_H_135_N_17_O_14_ [M + 2H]^2+^ 767.0182, found 767.0158; calcd for C_78_H_136_N_17_O_14_ [M + 3H]^3+^ 511.6812, found 511.6821; calcd for C_78_H_137_N_17_O_14_ [M + 4H]^4+^ 384.0127, found 384.0114.

#### Ac-Lys-Lys-Leu-Phe-Lys-Lys(COC_3_H_7_)-Ile-Leu-Lys-Tyr-Leu-NH_2_ (BP385)

This lipopeptide was prepared following the procedure described in the manuscript using butyric acid. Acidolytic cleavage of the resulting resin and purification eluting with H_2_O/CH_3_CN (75:25) afforded Ac-Lys-Lys-Leu-Phe-Lys-Lys(COC_3_H_7_)-Ile-Leu-Lys-Tyr-Leu-NH_2_ (**BP385**) in >99% purity. *t*_R_ = 7.02 min. MS (ESI) *m/z*: 1533.2 [M + H]^+^, 1555.1 [M + Na]^+^; HRMS (ESI) *m/z*: calcd for C_78_H_135_N_17_O_14_ [M + 2H]^2+^ 767.0182, found 767.0189; calcd for C_78_H_136_N_17_O_14_ [M + 3H]^3+^ 511.6812, found 511.6818; calcd for C_78_H_137_N_17_O_14_ [M + 4H]^4+^ 384.0127, found 384.0140.

#### Ac-Lys-Lys-Leu-Phe-Lys-Lys-Lys(COC_3_H_7_)-Leu-Lys-Tyr-Leu-NH_2_ (BP386)

This lipopeptide was prepared following the procedure described in the manuscript using butyric acid. Acidolytic cleavage of the resulting resin and purification eluting with H_2_O/CH_3_CN (85:15) afforded Ac-Lys-Lys-Leu-Phe-Lys-Lys-Lys(COC_3_H_7_)-Leu-Lys-Tyr-Leu-NH_2_ (**BP386**) in >99% purity. *t*_R_ = 6.25 min. MS (ESI) *m/z*: 1548.2 [M + H]^+^, 1570.1 [M + Na]^+^; HRMS (ESI) *m/z*: calcd for C_78_H_136_N_18_O_14_ [M + 2H]^2+^ 774.5236, found 774.5234; calcd for C_78_H_137_N_18_O_14_ [M + 3H]^3+^ 516.6848, found 516.6867; calcd for C_78_H_138_N_18_O_14_ [M + 4H]^4+^ 387.7654, found 387.7666.

#### Ac-Lys-Lys-Leu-Phe-Lys-Lys-Ile-Lys(COC_3_H_7_)-Lys-Tyr-Leu-NH_2_ (BP387)

This lipopeptide was prepared following the procedure described in the manuscript using butyric acid. Acidolytic cleavage of the resulting resin and purification eluting with H_2_O/CH_3_CN (80:20) afforded Ac-Lys-Lys-Leu-Phe-Lys-Lys-Ile-Lys(COC_3_H_7_)-Lys-Tyr-Leu-NH_2_ (**BP387**) in >99% purity. *t*_R_ = 6.49 min. MS (ESI) *m/z*: 774.5 [M + 2H]^2+^, 1549.0 [M + H]^+^; HRMS (ESI) *m/z*: calcd for C_78_H_137_N_18_O_14_ [M + 3H]^3+^ 516.6848, found 516.6839; calcd for C_78_H_138_N_18_O_14_ [M + 4H]^4+^ 387.7654, found 387.7648.

#### Ac-Lys-Lys-Leu-Phe-Lys-Lys-Ile-Leu-Lys(COC_3_H_7_)-Tyr-Leu-NH_2_ (BP388)

This lipopeptide was prepared following the procedure described in the manuscript using butyric acid. Acidolytic cleavage of the resulting resin and purification eluting with H_2_O/CH_3_CN (75:25) afforded Ac-Lys-Lys-Leu-Phe-Lys-Lys-Ile-Leu-Lys(COC_3_H_7_)-Tyr-Leu-NH_2_ (**BP388**) in >99% purity. *t*_R_ = 6.80 min. MS (ESI) *m/z*: 767.1 [M + 2H]^2+^, 1533.1 [M + H]^+^, 1555.1 [M + Na]^+^; HRMS (ESI) *m/z*: calcd for C_78_H_134_N_17_O_14_Na [M + H + Na]^2+^ 778.0091, found 778.0070; calcd for C_78_H_135_N_17_O_14_ [M + 2H]^2+^ 767.0182, found 767.0160; calcd for C_78_H_135_N_17_O_14_Na [M + 2H + Na]^3+^ 519.0085, found 519.0070; calcd for C_78_H_136_N_17_O_14_ [M + 3H]^3+^ 511.6812, found 511.6805.

#### Ac-Lys-Lys-Leu-Phe-Lys-Lys-Ile-Leu-Lys-Lys(COC_3_H_7_)-Leu-NH_2_ (BP389)

This lipopeptide was prepared following the procedure described in the manuscript using butyric acid. Acidolytic cleavage of the resulting resin and purification eluting with H_2_O/CH_3_CN (75:25) afforded Ac-Lys-Lys-Leu-Phe-Lys-Lys-Ile-Leu-Lys-Lys(COC_3_H_7_)-Leu-NH_2_ (**BP389**) in >99% purity. *t*_R_ = 6.73 min. MS (ESI) *m/z*: 1498.2 [M + H]^+^; HRMS (ESI) *m/z*: calcd for C_75_H_138_N_18_O_13_ [M + 2H]^2+^ 749.5340, found 749.5328; calcd for C_75_H_139_N_18_O_13_ [M + 3H]^3+^ 500.0251, found 500.0268; calcd for C_75_H_140_N_18_O_13_ [M + 4H]^4+^ 375. 2706, found 375.2692.

#### Ac-Lys-Lys-Leu-Phe-Lys-Lys-Ile-Leu-Lys-Tyr-Lys(COC_3_H_7_)-NH_2_ (BP390)

This lipopeptide was prepared following the procedure described in the manuscript using butyric acid. Acidolytic cleavage of the resulting resin and purification eluting with H_2_O/CH_3_CN (80:20) afforded Ac-Lys-Lys-Leu-Phe-Lys-Lys-Ile-Leu-Lys-Tyr-Lys(COC_3_H_7_)-NH_2_ (**BP390**) in >99% purity. *t*_R_ = 6.27 min. MS (ESI) *m/z*: 516.7 [M + 3H]^3+^, 774.6 [M + 2H]^2+^, 1548.2 [M + H]^+^, 1570.1 [M + Na]^+^ ; HRMS (ESI) *m/z*: calcd for C_86_H_136_N_18_O_14_ [M + 2H]^2+^ 774.5236, found 774.5216; calcd for C_78_H_137_N_18_O_14_ [M + 3H]^3+^ 516.6848, found 516.6843; calcd for C_78_H_138_N_18_O_14_ [M + 4H]^4+^ 387.7654, found 387.7665.

#### C_11_H_23_CO-Lys-Lys-Leu-Phe-Lys-Lys-Ile-Leu-Lys-Tyr-Leu-NH_2_ (BP391)

This lipopeptide was prepared following the procedure described in the manuscript using lauric acid. Acidolytic cleavage of the resulting resin and purification eluting with H_2_O/CH_3_CN (70:30) afforded C_11_H_23_CO-Lys-Lys-Leu-Phe-Lys-Lys-Ile-Leu-Lys-Tyr-Leu-NH_2_ (**BP391**) in >99% purity. *t*_R_ = 8.06 min. MS (ESI) *m/z*: 535.4 [M + 3H]^3+^, 802.6 [H + 2H]^2+^, 1604.2 [M + H]^+^; HRMS (ESI) *m/z*: calcd for C_84_H_149_N_17_O_13_ [M + 2H]^2+^ 802.0755, found 802.0738; calcd for C_84_H_150_N_17_O_13_ [M + 3H]^3+^ 535.0528, found 535.0527; calcd for C_84_H_151_N_17_O_13_ [M + 4H]^4+^ 401.5414, found 401.5427.

#### Ac-Lys(COC_11_H_23_)-Lys-Leu-Phe-Lys-Lys-Ile-Leu-Lys-Tyr-Leu-NH_2_ (BP392)

This lipopeptide was prepared following the procedure described in the manuscript using lauric acid. Acidolytic cleavage of the resulting resin and purification eluting with H_2_O/CH_3_CN (70:30) afforded Ac-Lys(COC_11_H_23_)-Lys-Leu-Phe-Lys-Lys-Ile-Leu-Lys-Tyr-Leu-NH_2_ (**BP392**) in >99% purity. *t*_R_ = 8.25 min. MS (ESI) *m/z*: 549.1 [M + 3H]^3+^, 823.1 [M + 2H]^2+^, 1646.2 [M + H]^+^; HRMS (ESI) *m/z*: calcd for C_86_H_151_N_17_O_14_ [M + 2H]^2+^ 823.0808, found 823.0808; calcd for C_86_H_152_N_17_O_14_ [M + 3H]^3+^ 549.0563, found 549.0558; calcd for C_86_H_153_N_17_O_14_ [M + 4H]^4+^ 412.0440, found 412.0437.

#### Ac-Lys-Lys(COC_11_H_23_)-Leu-Phe-Lys-Lys-Ile-Leu-Lys-Tyr-Leu-NH_2_ (BP393)

This lipopeptide was prepared following the procedure described in the manuscript using lauric acid. Acidolytic cleavage of the resulting resin and purification eluting with H_2_O/CH_3_CN (80:20) afforded Ac-Lys-Lys(COC_11_H_23_)-Leu-Phe-Lys-Lys-Ile-Leu-Lys-Tyr-Leu-NH_2_ (**BP393**) in >99% purity. *t*_R_ = 7.68 min. MS (ESI) *m/z*: 549.3 [M + 3H]^3+^, 823.5 [M + 2H]^2+^, 1645.3 [M + H]^+^, 1667.1 [M + Na]^+^; HRMS (ESI) *m/z*: calcd for C_86_H_151_N_17_O_14_ [M + 2H]^2+^ 823.0808, found 823.0789; calcd for C_86_H_152_N_17_O_14_ [M + 3H]^3+^ 549.0563, found 549.0548.

#### Ac-Lys-Lys-Lys(COC_11_H_23_)-Phe-Lys-Lys-Ile-Leu-Lys-Tyr-Leu-NH_2_ (BP394)

This lipopeptide was prepared following the procedure described in the manuscript using lauric acid. Acidolytic cleavage of the resulting resin and purification eluting with H_2_O/CH_3_CN (75:25) afforded Ac-Lys-Lys-Lys(COC_11_H_23_)-Phe-Lys-Lys-Ile-Leu-Lys-Tyr-Leu-NH_2_ (**BP394**) in 97% purity. *t*_R_ = 7.32 min. MS (ESI) *m/z*: 554.1 [M + 3H]^3+^, 831.1 [M + 2H]^2+^, 1661.3 [M + H]^+^, 1683.3 [M + Na]^+^; HRMS (ESI) *m/z*: calcd for C_86_H_152_N_18_O_14_ [M + 2H]^2+^ 830.5862, found 830.5847; calcd for C_86_H_153_N_18_O_14_ [M + 3H]^3+^ 554.0599, found 554.0601; calcd for C_86_H_154_N_18_O_14_ [M + 4H]^4+^ 415.7967, found 415.7987.

#### Ac-Lys-Lys-Leu-Lys(COC_11_H_23_)-Lys-Lys-Ile-Leu-Lys-Tyr-Leu-NH_2_ (BP395)

This lipopeptide was prepared following the procedure described in the manuscript using lauric acid. Acidolytic cleavage of the resulting resin and purification eluting with H_2_O/CH_3_CN (75:25) afforded Ac-Lys-Lys-Leu-Lys(COC_11_H_23_)-Lys-Lys-Ile-Leu-Lys-Tyr-Leu-NH_2_ (**BP395**) in >99% purity. *t*_R_ = 7.39 min. MS (ESI) *m/z*: 1627.3 [M + H]^+^; HRMS (ESI) *m/z*: calcd for C_83_H_153_N_18_O_14_ [M + H]^+^ 1626.1808, found 1626.1755; calcd for C_83_H_154_N_18_O_14_ [M + 2H]^2+^ 813.5940, found 813.5911.

#### Ac-Lys-Lys-Leu-Phe-Lys(COC_11_H_23_)-Lys-Ile-Leu-Lys-Tyr-Leu-NH_2_ (BP396)

This lipopeptide was prepared following the procedure described in the manuscript using lauric acid. Acidolytic cleavage of the resulting resin and purification eluting with H_2_O/CH_3_CN (70:30) afforded Ac-Lys-Lys-Leu-Phe-Lys(COC_11_H_23_)-Lys-Ile-Leu-Lys-Tyr-Leu-NH_2_ (**BP396**) in >99% purity. *t*_R_ = 7.76 min. MS (ESI) *m/z*: 549.4 [M + 3H]^3+^, 823.1 [M + 2H]^2+^, 1646.3 [M + H]^+^; HRMS (ESI) *m/z*: calcd for C_86_H_151_N_17_O_14_ [M + 2H]^2+^ 823.0808, found 823.0791; calcd for C_86_H_152_N_17_O_14_ [M + 3H]^3+^ 549.0563, found 549.0570; calcd for C_86_H_153_N_17_O_14_ [M + 4H]^4+^ 412.0440, found 412.0432.

#### Ac-Lys-Lys-Leu-Phe-Lys-Lys(COC_11_H_23_)-Ile-Leu-Lys-Tyr-Leu-NH_2_ (BP397)

This lipopeptide was prepared following the procedure described in the manuscript using lauric acid. Acidolytic cleavage of the resulting resin and purification eluting with H_2_O/CH_3_CN (70:30) afforded Ac-Lys-Lys-Leu-Phe-Lys-Lys(COC_11_H_23_)-Ile-Leu-Lys-Tyr-Leu-NH_2_ (**BP397**) in 97% purity. *t*_R_ = 8.10 min. MS (ESI) *m/z*: 549.4 [M + 3H]^3+^, 823.1 [M + 2H]^2+^, 1646.3 [M + H]^+^; HRMS (ESI) *m/z*: calcd for C_86_H_151_N_17_O_14_ [M + 2H]^2+^ 823.0808, found 823.0806; calcd for C_86_H_152_N_17_O_14_ [M + 3H]^3+^ 549.0563, found 549.0586; calcd for C_86_H_153_N_17_O_14_ [M + 4H]^4+^ 412.0440, found 412.0444.

#### Ac-Lys-Lys-Leu-Phe-Lys-Lys-Lys(COC_11_H_23_)-Leu-Lys-Tyr-Leu-NH_2_ (BP398)

This lipopeptide was prepared following the procedure described in the manuscript using lauric acid. Acidolytic cleavage of the resulting resin and purification eluting with H_2_O/CH_3_CN (80:20) afforded Ac-Lys-Lys-Leu-Phe-Lys-Lys-Lys(COC_11_H_23_)-Leu-Lys-Tyr-Leu-NH_2_ (**BP398**) in >99% purity. *t*_R_ = 7.26 min. MS (ESI) *m/z*: 554.1 [M + 3H]^3+^, 830.6 [M + 2H]^2+^, 1660.3 [M + H]^+^, 1682.3 [M + Na]^+^; HRMS (ESI) *m/z*: calcd for C_86_H_152_N_18_O_14_ [M + 2H]^2+^ 830.5862, found 830.5852; calcd for C_86_H_153_N_18_O_14_ [M + 3H]^3+^ 554.0599, found 554.0602; calcd for C_86_H_154_N_18_O_14_ [M + 4H]^4+^ 415.7967, found 415.7972.

#### Ac-Lys-Lys-Leu-Phe-Lys-Lys-Ile-Lys(COC_11_H_23_)-Lys-Tyr-Leu-NH_2_ (BP399)

This lipopeptide was prepared following the procedure described in the manuscript using lauric acid. Acidolytic cleavage of the resulting resin and purification eluting with H_2_O/CH_3_CN (75:25) afforded Ac-Lys-Lys-Leu-Phe-Lys-Lys-Ile-Lys(COC_11_H_23_)-Lys-Tyr-Leu-NH_2_ (**BP399**) in >99% purity. *t*_R_ = 7.38 min. MS (ESI) *m/z*: 554.1 [M + 3H]^3+^, 831.1 [M + 2H]^2+^, 1660.3 [M + H]^+^, 1682.3 [M + Na]^+^; HRMS (ESI) *m/z*: calcd for C_86_H_152_N_18_O_14_ [M + 2H]^2+^ 830.5862, found 830.5847; calcd for C_86_H_153_N_18_O_14_ [M + 3H]^3+^ 554.0599, found 554.0593; calcd for C_86_H_154_N_18_O_14_ [M + 4H]^4+^ 415.7967, found 415.7968.

#### Ac-Lys-Lys-Leu-Phe-Lys-Lys-Ile-Leu-Lys(COC_11_H_23_)-Tyr-Leu-NH_2_ (BP400)

This lipopeptide was prepared following the procedure described in the manuscript using lauric acid. Acidolytic cleavage of the resulting resin and purification eluting with H_2_O/CH_3_CN (75:25) afforded Ac-Lys-Lys-Leu-Phe-Lys-Lys-Ile-Leu-Lys(COC_11_H_23_)-Tyr-Leu-NH_2_ (**BP400**) in >99% purity. *t*_R_ = 7.60 min. MS (ESI) *m/z*: 823.1 [M + 2H]^2+^, 1645.3 [M + H]^+^, 1667.3 [M + Na]^+^; HRMS (ESI) *m/z*: calcd for C_86_H_151_N_17_O_14_ [M + 2H]^2+^ 823.0808, found 823.0796; calcd for C_86_H_152_N_17_O_14_ [M + 3H]^3+^ 549.0563, found 549.0565; calcd for C_86_H_153_N_17_O_14_ [M + 4H]^4+^ 412.0440, found 412.0434.

#### Ac-Lys-Lys-Leu-Phe-Lys-Lys-Ile-Leu-Lys-Lys(COC_11_H_23_)-Leu-NH_2_ (BP401)

This lipopeptide was prepared following the procedure described in the manuscript using lauric acid. Acidolytic cleavage of the resulting resin and purification eluting with H_2_O/CH_3_CN (75:25) afforded Ac-Lys-Lys-Leu-Phe-Lys-Lys-Ile-Leu-Lys-Lys(COC_11_H_23_)-Leu-NH_2_ (**BP401**) in >99% purity. *t*_R_ = 7.77 min. MS (ESI) *m/z*: 537.5 [M + 3H]^3+^, 806.1 [M + 2H]^2+^, 1610.3 [M + H]^+^, 1632.3 [M + Na]^+^; HRMS (ESI) *m/z*: calcd for C_83_H_154_N_18_O_13_ [M + 2H]^2+^ 805.5966, found 805.5955; calcd for C_83_H_155_N_18_O_13_ [M + 3H]^3+^ 537.4002, found 537.3999; calcd for C_83_H_156_N_18_O_13_ [M + 4H]^4+^ 403.3019, found 403.3019.

#### Ac-Lys-Lys-Leu-Phe-Lys-Lys-Ile-Leu-Lys-Tyr-Lys(COC_11_H_23_)-NH_2_ (BP402)

This lipopeptide was prepared following the procedure described in the manuscript using lauric acid. Acidolytic cleavage of the resulting resin and purification eluting with H_2_O/CH_3_CN (75:25) afforded Ac-Lys-Lys-Leu-Phe-Lys-Lys-Ile-Leu-Lys-Tyr-Lys(COC_11_H_23_)-NH_2_ (**BP402**) in 99% purity. *t*_R_ = 7.29 min. MS (ESI) *m/z*: 554.4 [M + 3H]^3+^, 831.1 [M + 2H]^2+^, 1661.3 [M + H]^+^; HRMS (ESI) *m/z*: calcd for C_86_H_152_N_18_O_14_ [M + 2H]^2+^ 830.5862, found 830.5866; calcd for C_86_H_153_N_18_O_14_ [M + 3H]^3+^ 554.0599, found 554.0601; calcd for C_86_H_154_N_18_O_14_ [M + 4H]^4+^ 415.7967, found 415.7969.

**3. Characterization of lipopeptides BP367-BP402: HPLC of crude and purified lipopeptides, ESI-MS and HRMS**

### C_5_H_11_CO-Lys-Lys-Leu-Phe-Lys-Lys-Ile-Leu-Lys-Tyr-Leu-NH_2_ (BP367)

HPLC of crude peptide (λ=220 nm)


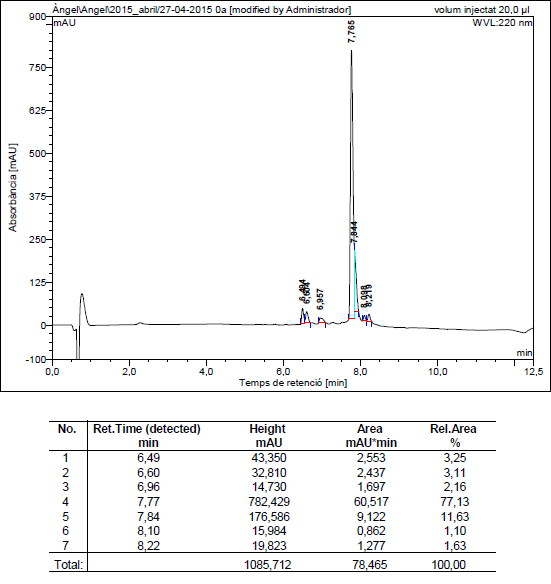


HPLC of purified peptide (λ=220 nm)


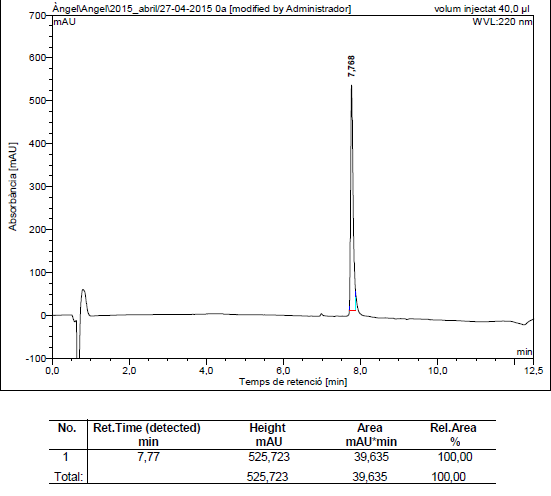


ESI-MS (*m/z*)


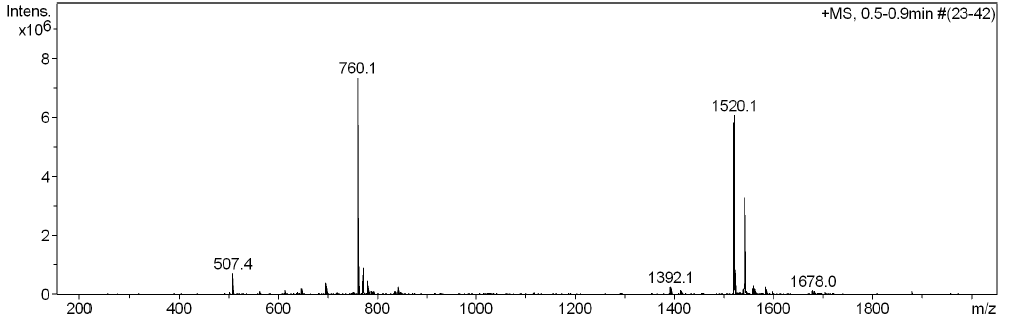


HRMS (*m/z*)


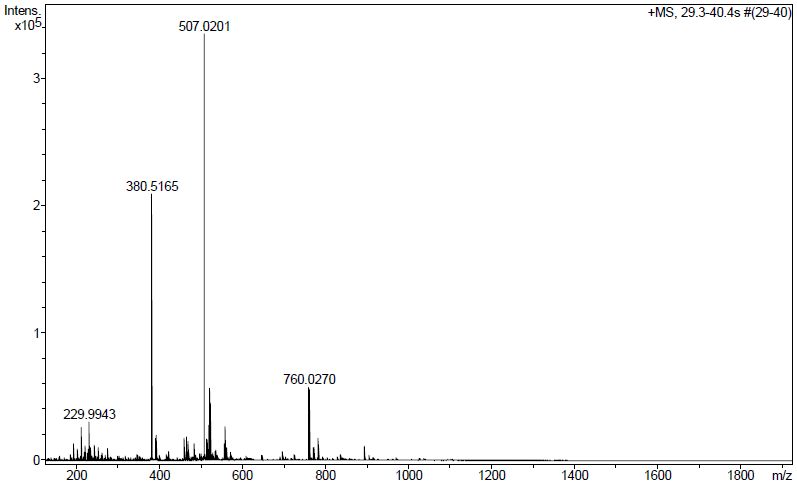


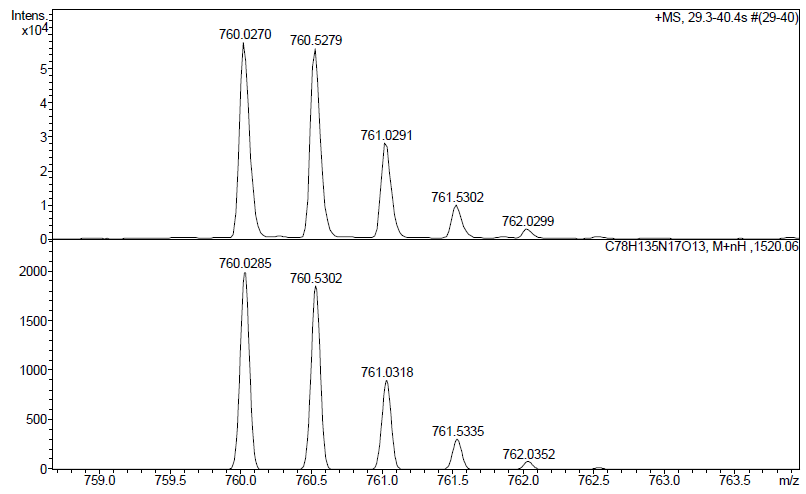


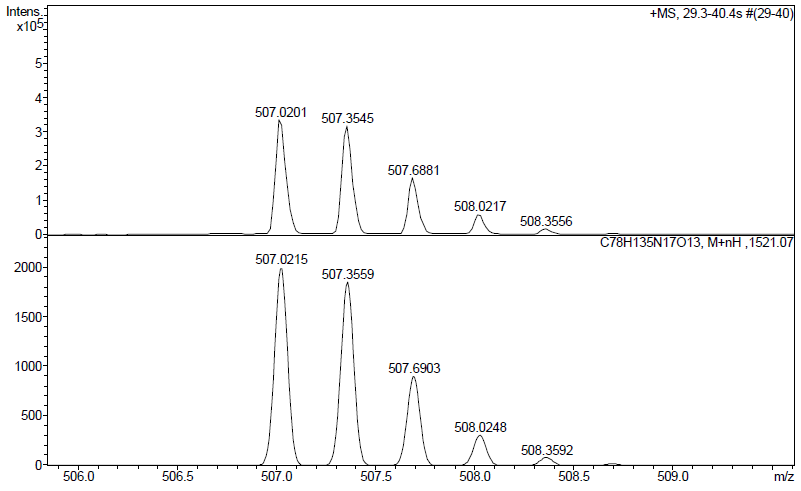


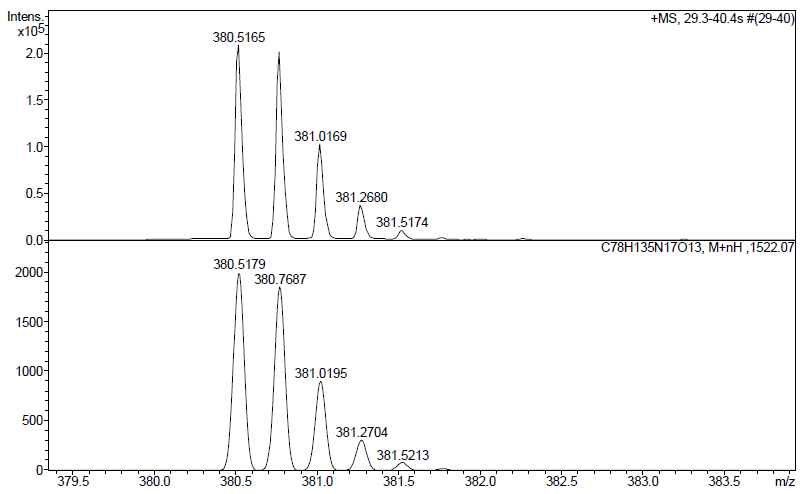


### Ac-Lys(COC_5_H_11_)-Lys-Leu-Phe-Lys-Lys-Ile-Leu-Lys-Tyr-Leu-NH_2_ (BP368)

HPLC of crude peptide (λ=220 nm)


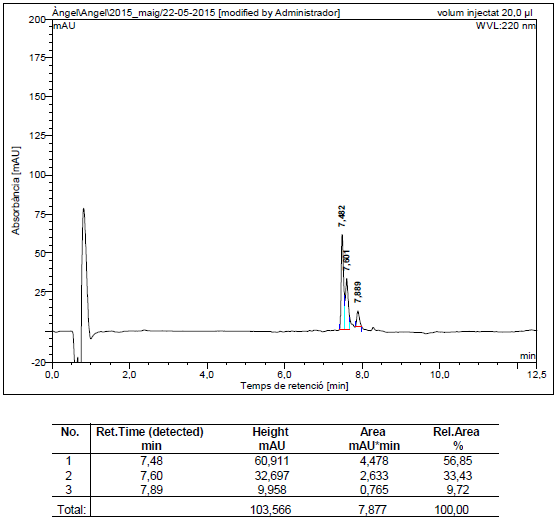


HPLC of purified peptide (λ=220 nm)


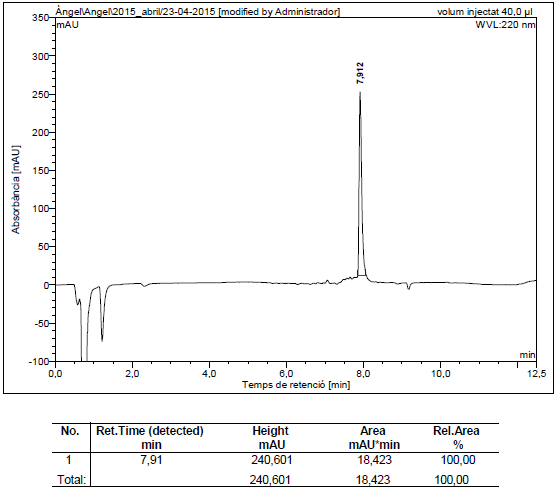


ESI-MS (*m/z*)


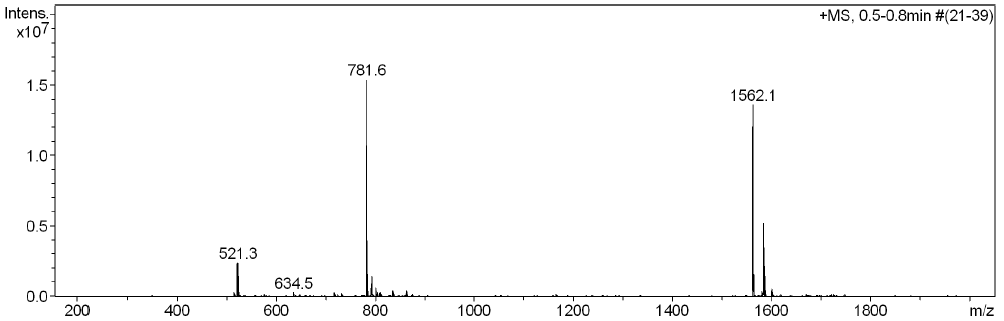


HRMS (*m/z*)


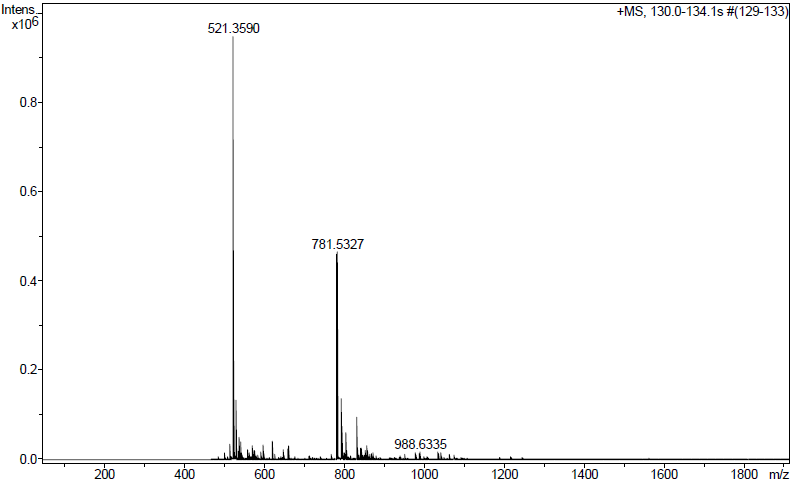


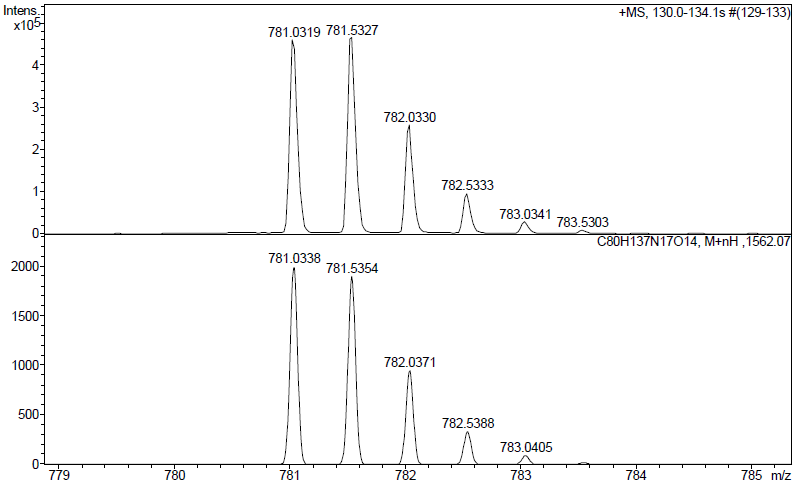


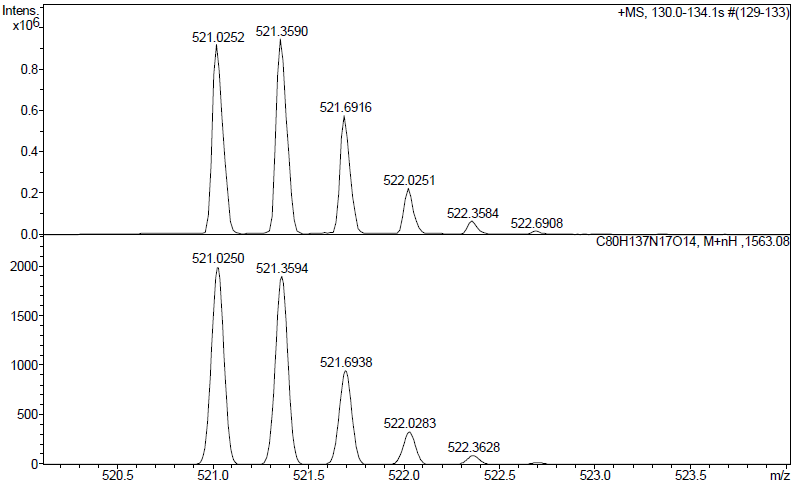


### Ac-Lys-Lys(COC_5_H_11_)-Leu-Phe-Lys-Lys-Ile-Leu-Lys-Tyr-Leu-NH_2_ (BP369)

HPLC of crude peptide (λ=220 nm)


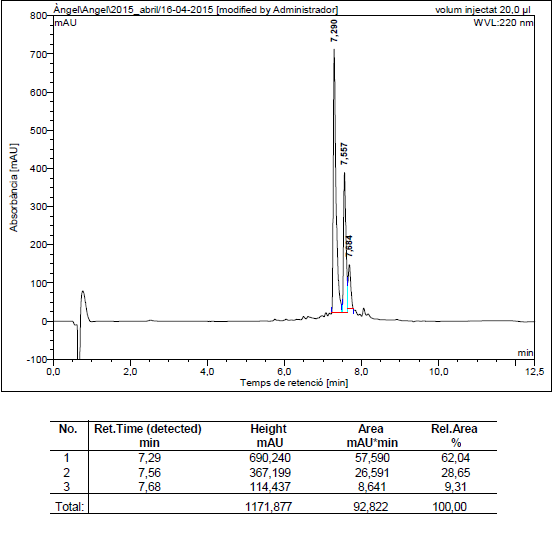


HPLC of purified peptide (λ=220 nm)


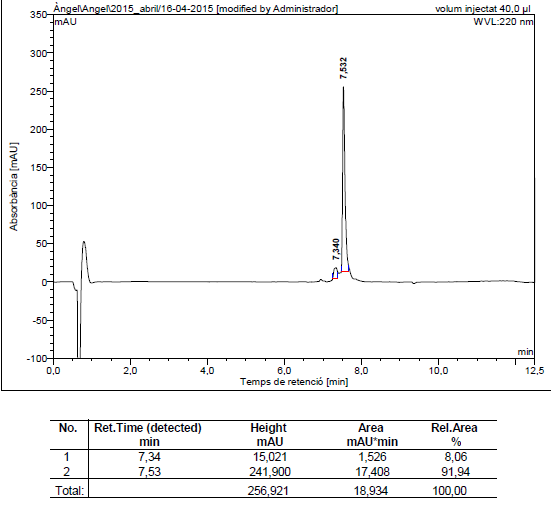


ESI-MS (*m/z*)


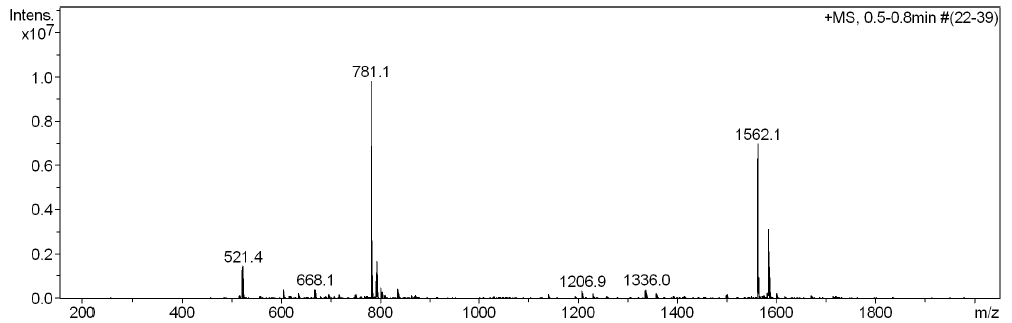


HRMS (*m/z*)


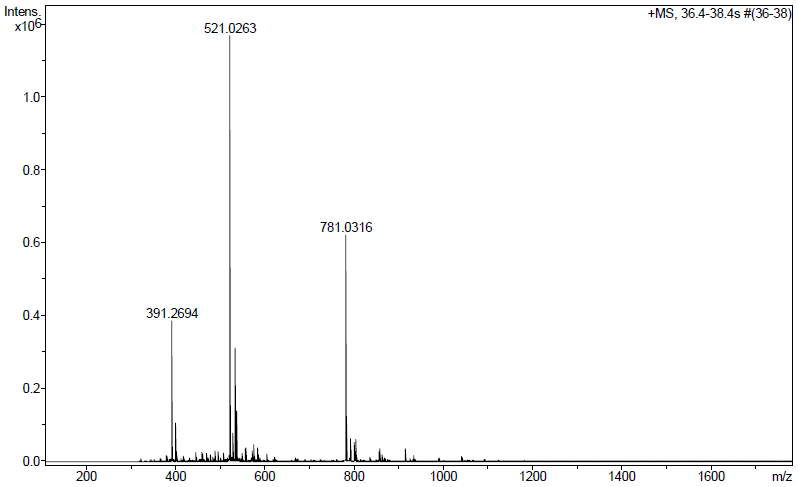


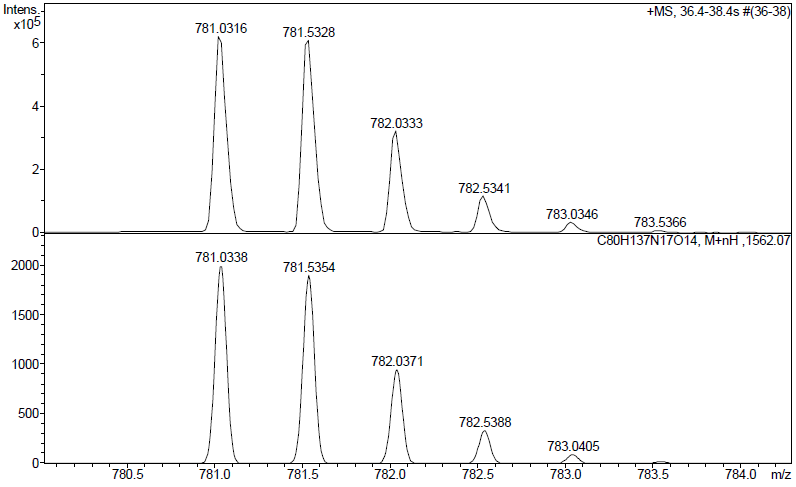


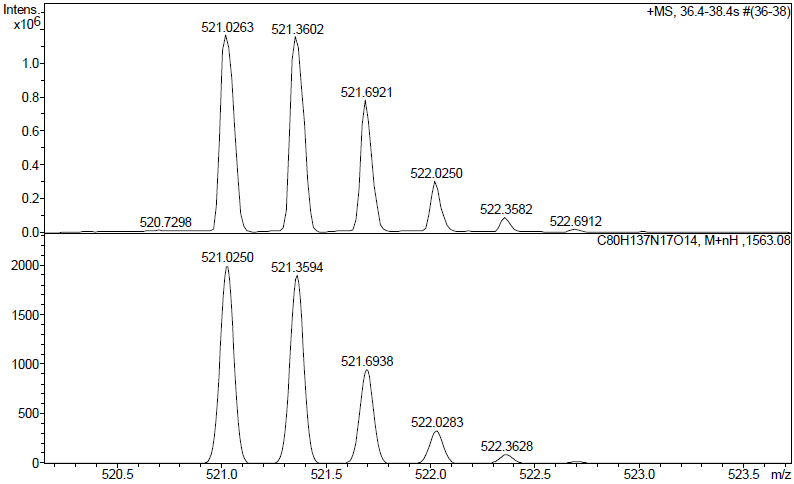


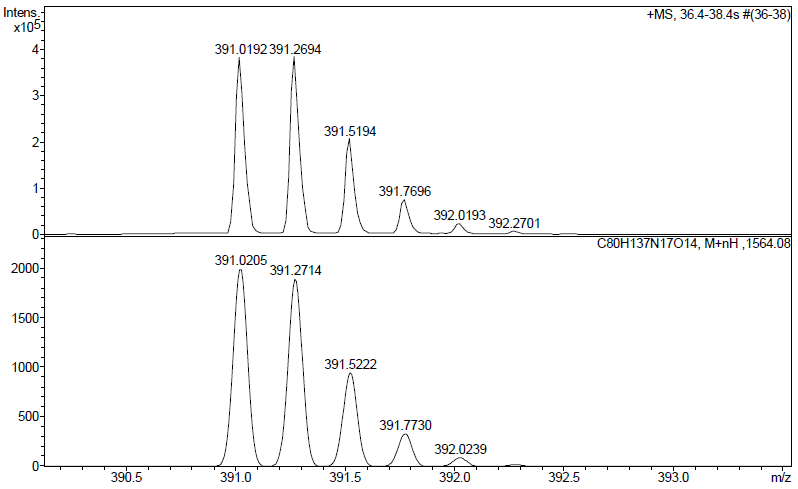


### Ac-Lys-Lys-Lys(COC_5_H_11_)-Phe-Lys-Lys-Ile-Leu-Lys-Tyr-Leu-NH_2_ (BP370)

HPLC of crude peptide (λ=220 nm)


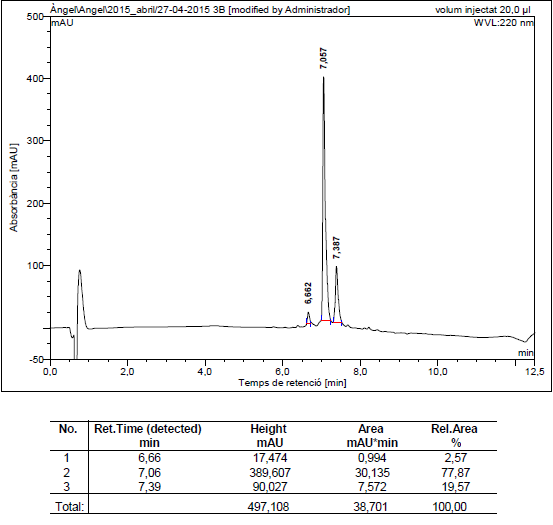


HPLC of purified peptide (λ=220 nm)


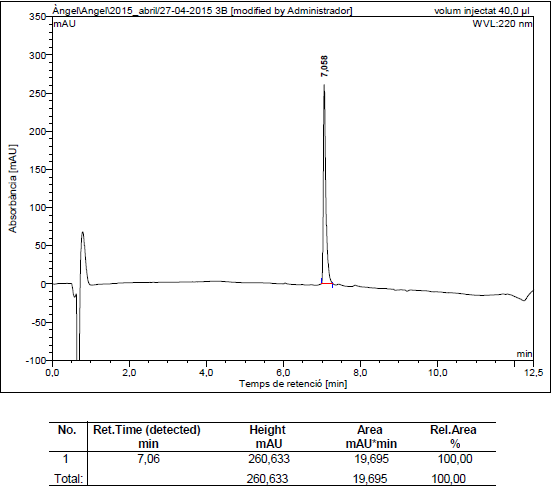


ESI-MS (*m/z*)


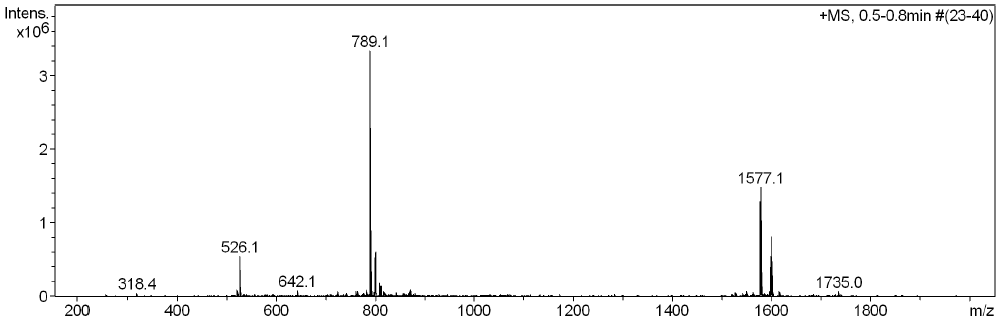


HRMS (*m/z*)


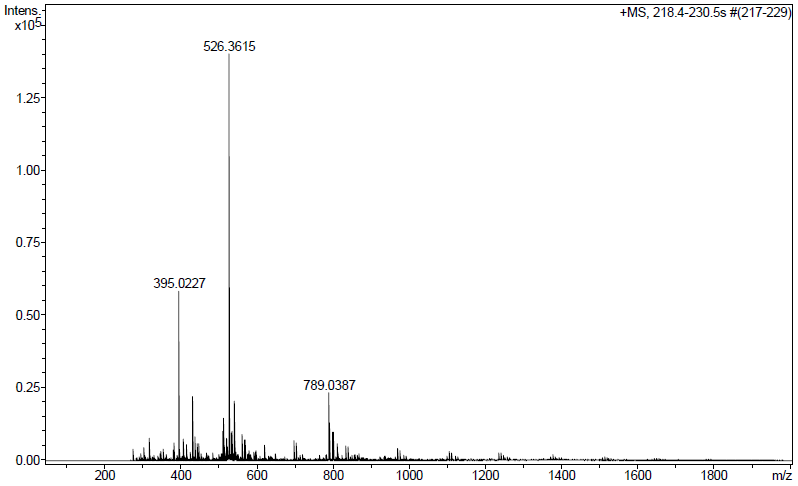


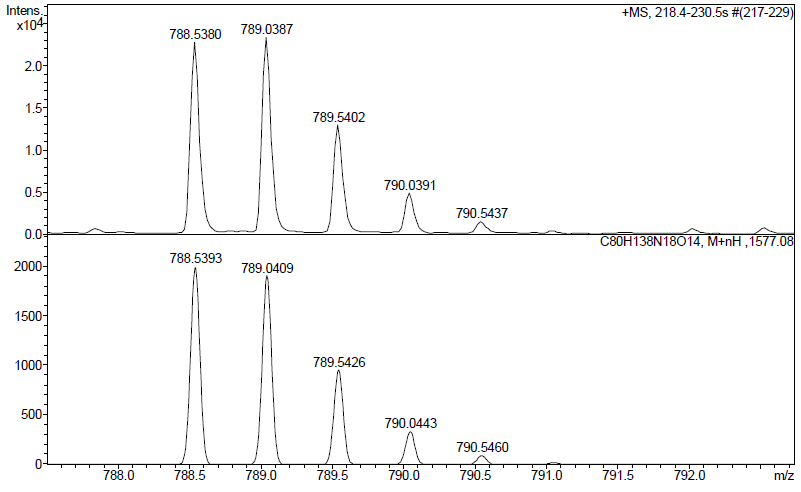


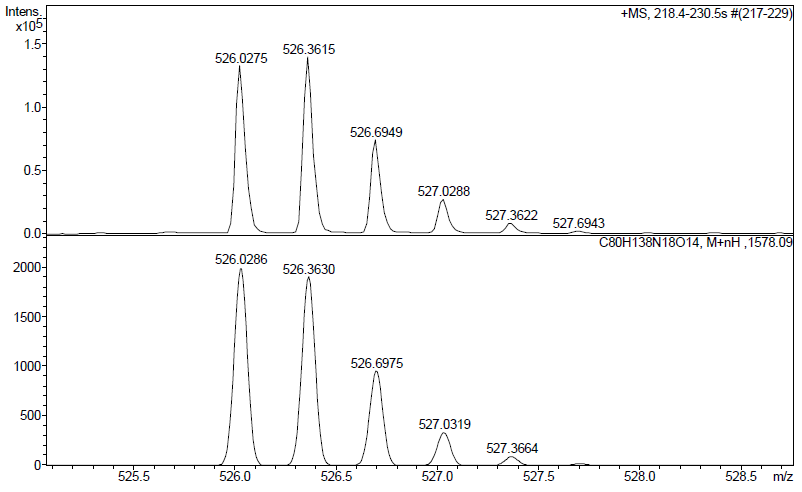


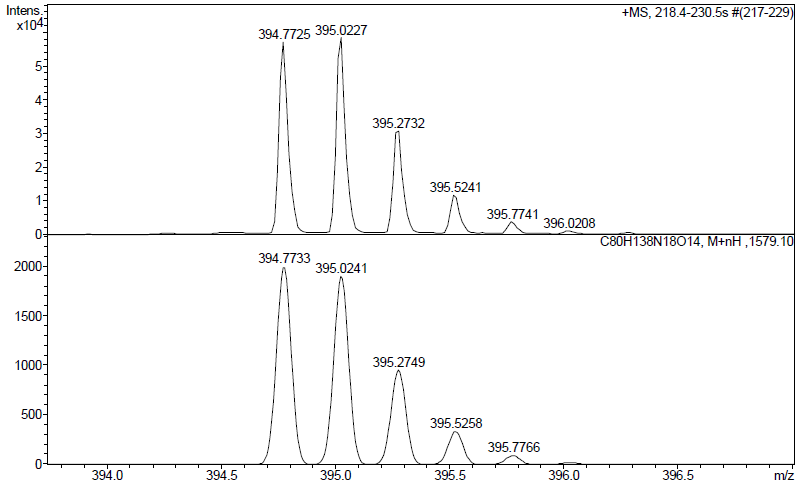


### Ac-Lys-Lys-Leu-Lys(COC_5_H_11_)-Lys-Lys-Ile-Leu-Lys-Tyr-Leu-NH_2_ (BP371)

HPLC of crude peptide (λ=220 nm)


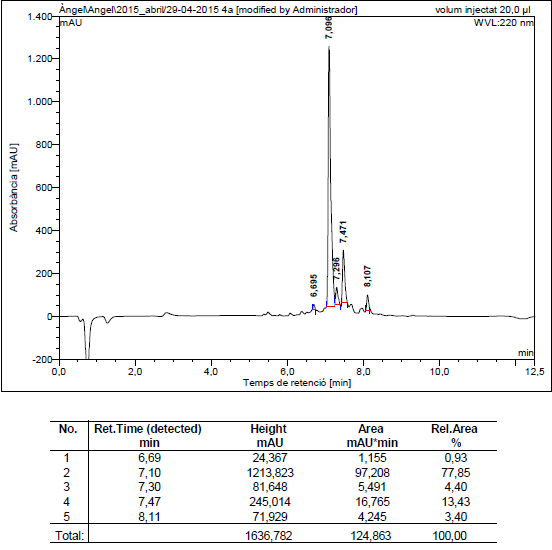


HPLC of purified peptide (λ=220 nm)


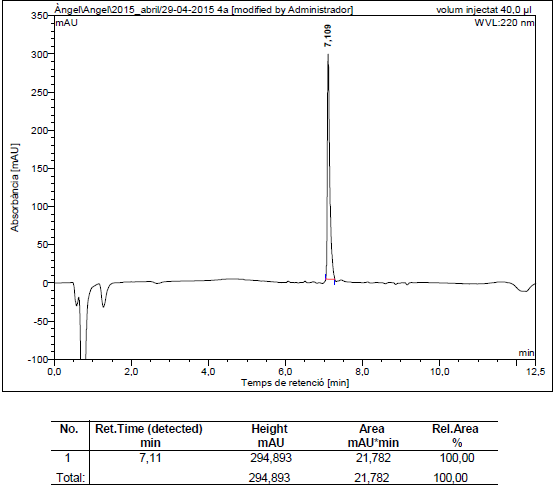


ESI-MS (*m/z*)


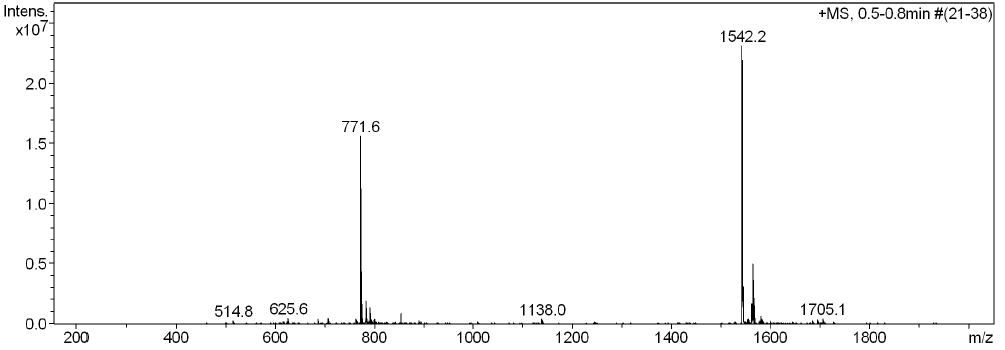


HRMS (*m/z*)


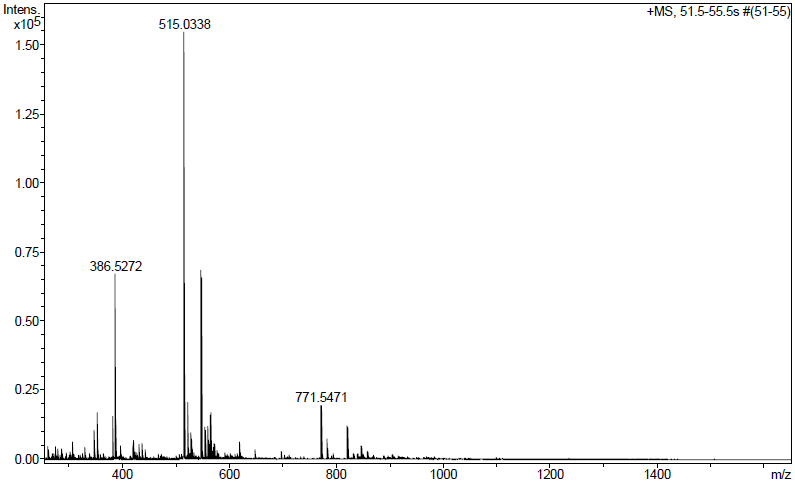


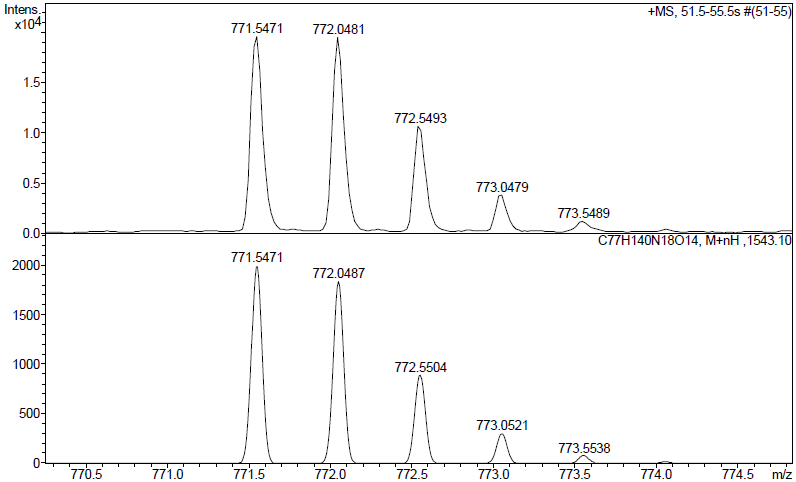


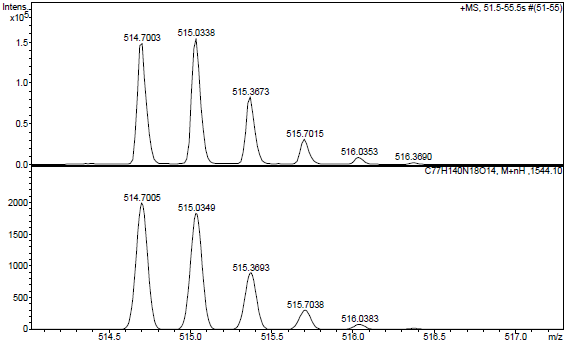


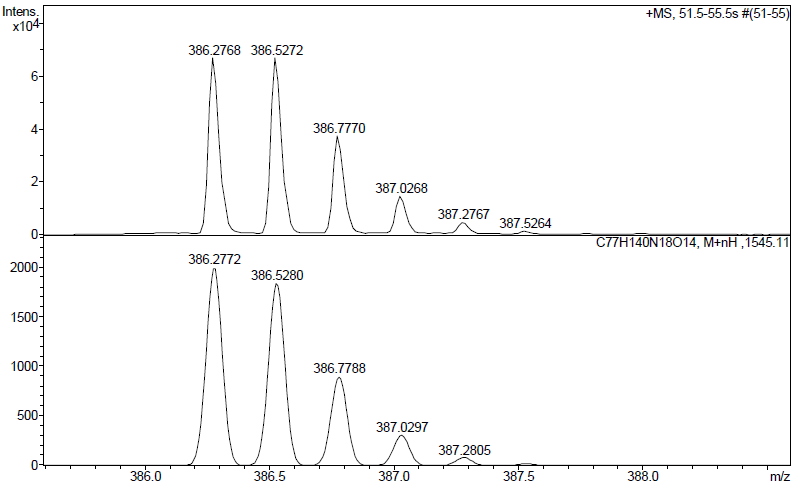


### Ac-Lys-Lys-Leu-Phe-Lys(COC_5_H_7_)-Lys-Ile-Leu-Lys-Tyr-Leu-NH_2_ (BP372)

HPLC of crude peptide (λ=220 nm)


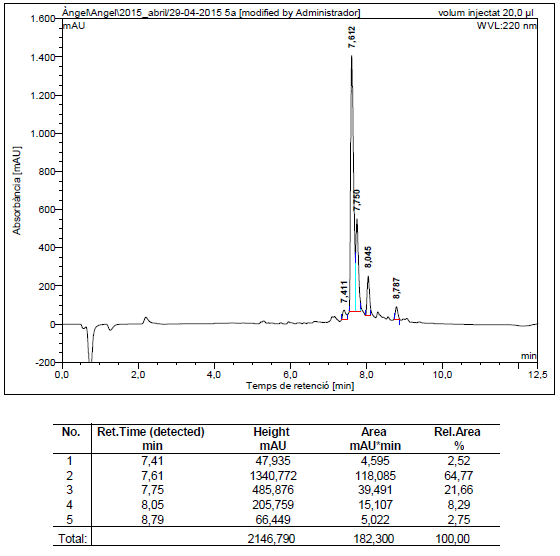


HPLC of purified peptide (λ=220 nm)


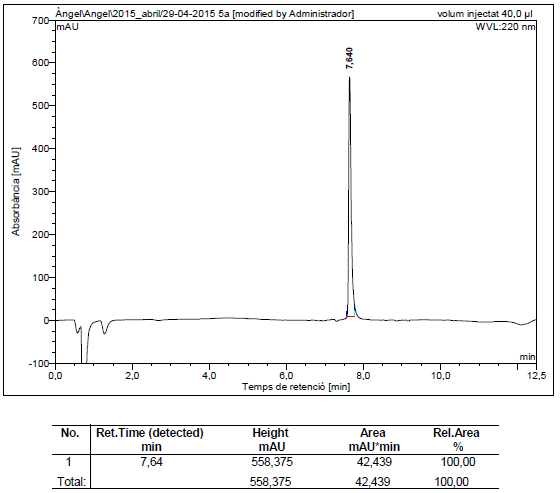


ESI-MS (*m/z*)


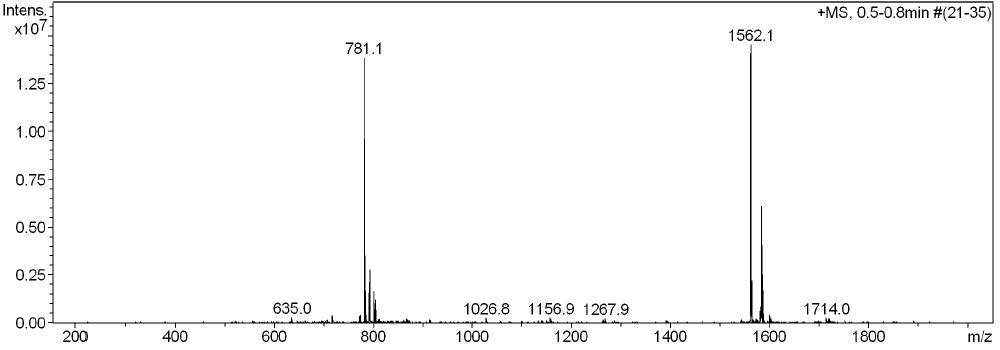


HRMS (*m/z*)


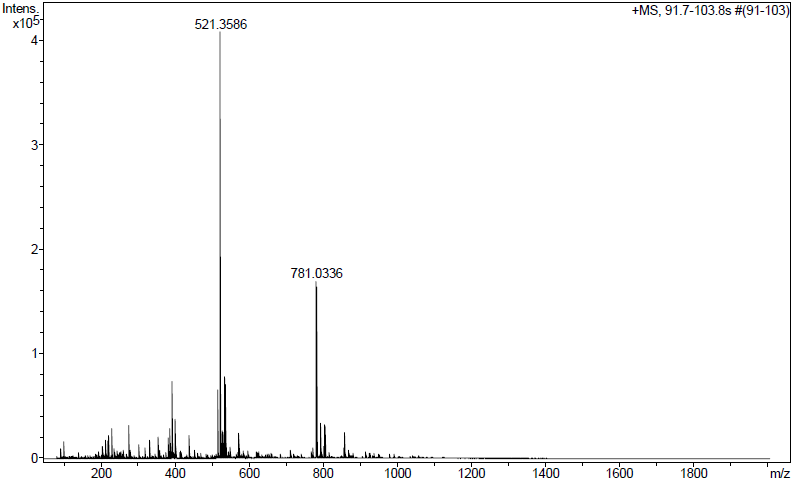


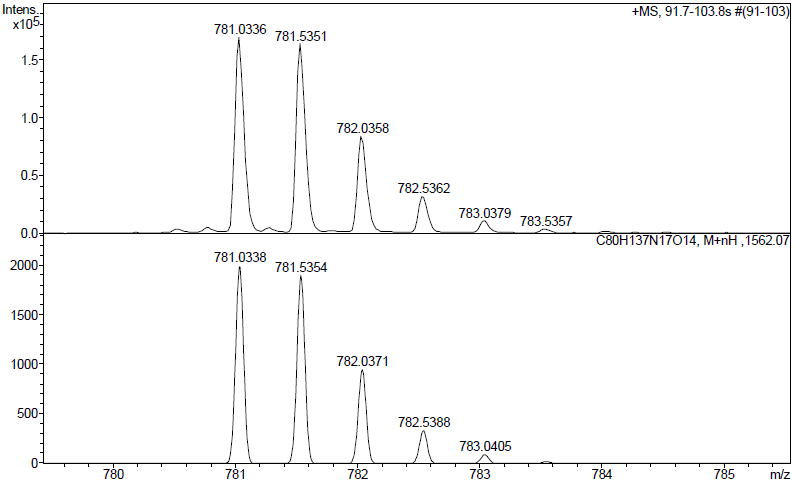


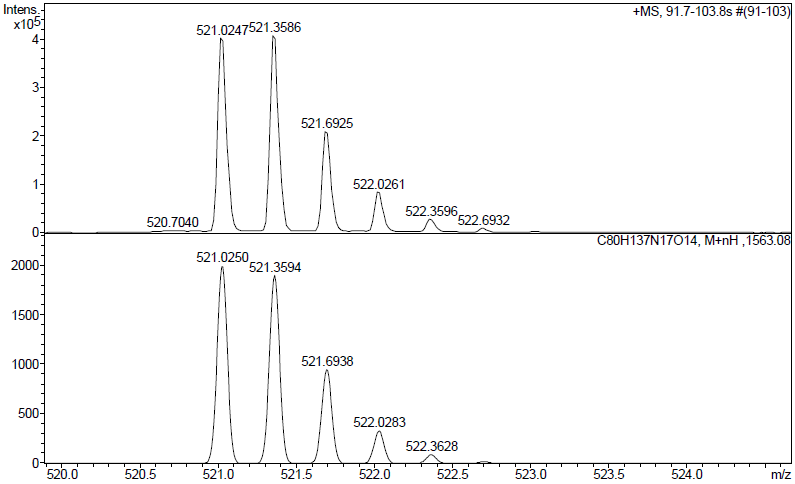


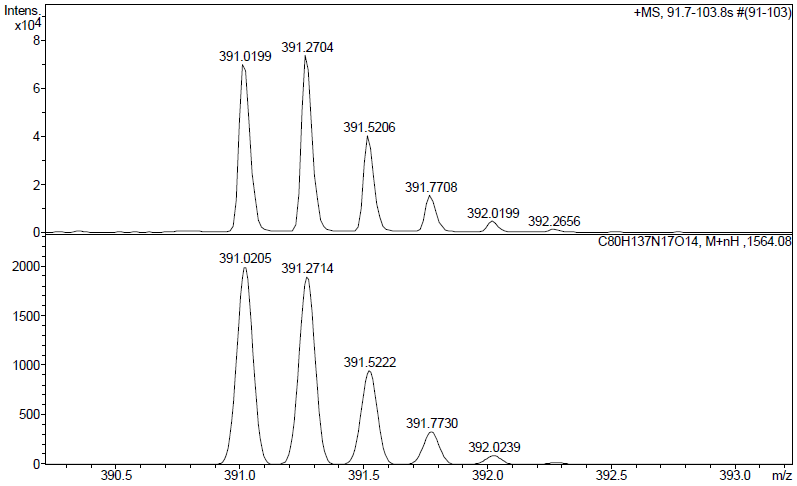


### Ac-Lys-Lys-Leu-Phe-Lys-Lys(COC_5_H_11_)-Ile-Leu-Lys-Tyr-Leu-NH_2_ (BP373)

HPLC of crude peptide (λ=220 nm)


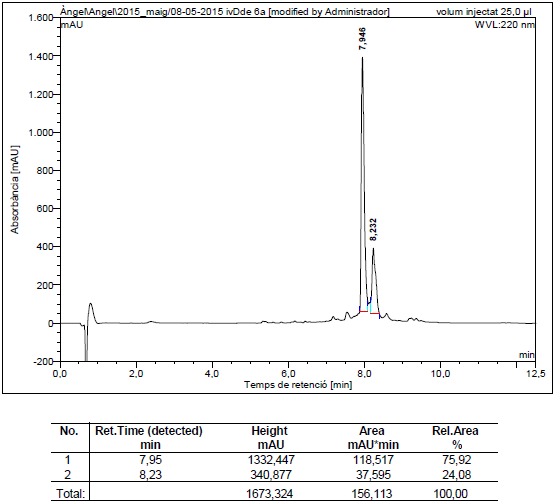


HPLC of purified peptide (λ=220 nm)


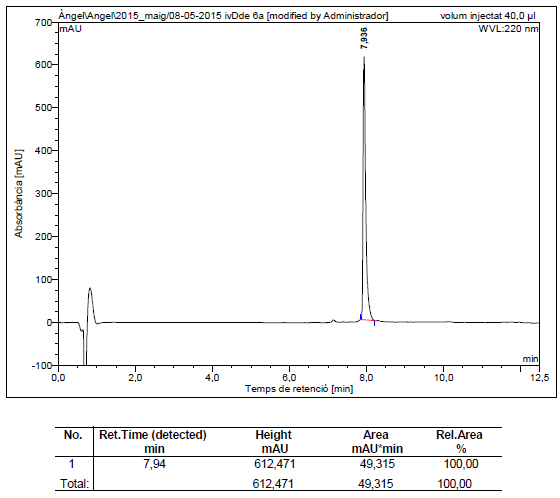


ESI-MS (*m/z*)


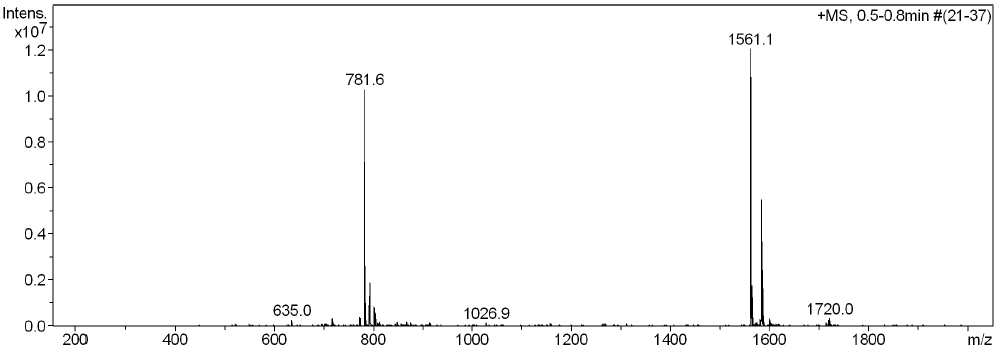


HRMS (*m/z*)


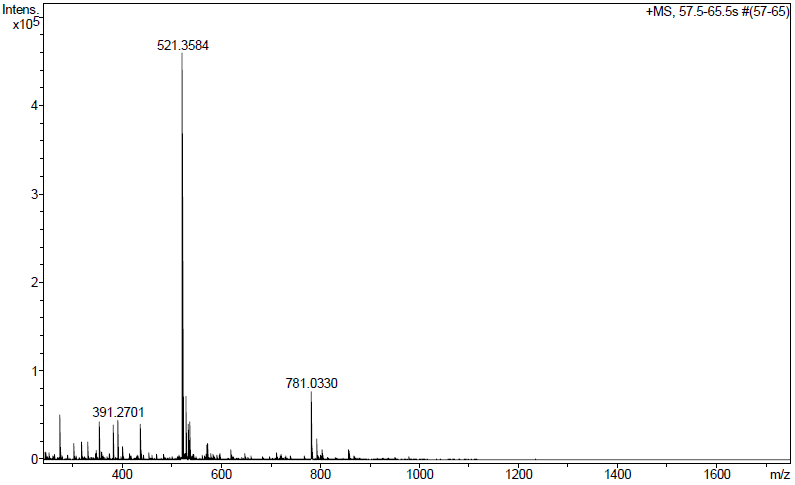


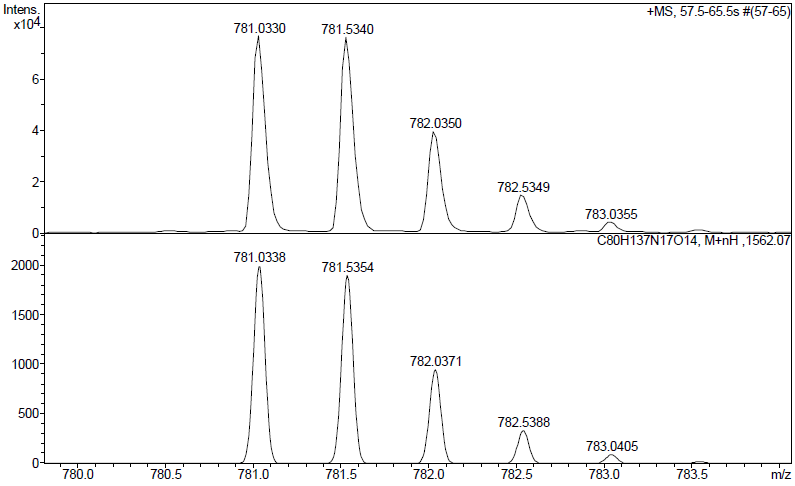

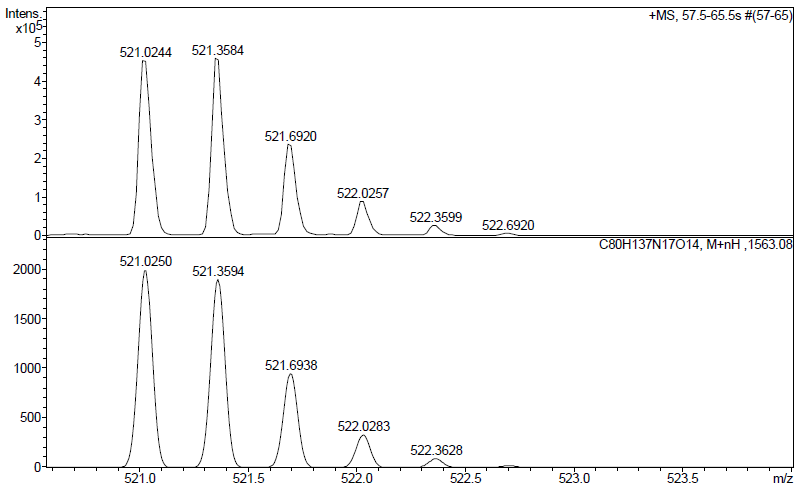


### Ac-Lys-Lys-Leu-Phe-Lys-Lys-Lys(COC_5_H_11_)-Leu-Lys-Tyr-Leu-NH_2_ (BP374)

HPLC of crude peptide (λ=220 nm)


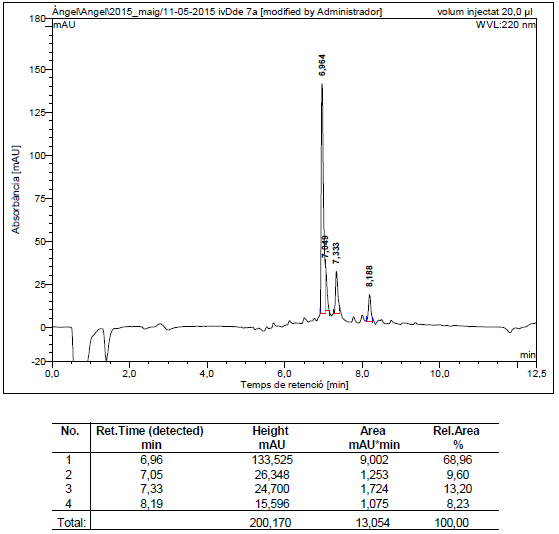


HPLC of purified peptide (λ=220 nm)


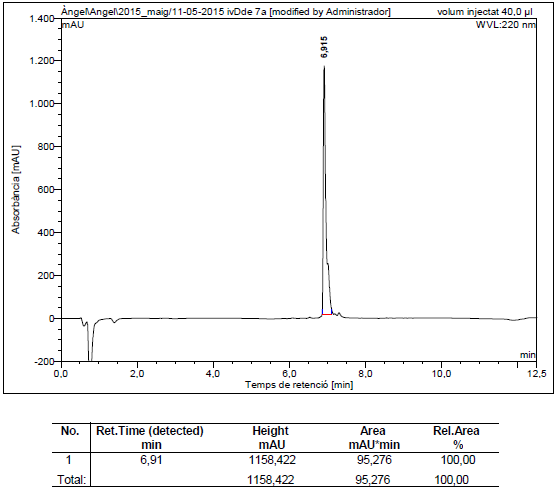


ESI-MS (*m/z*)


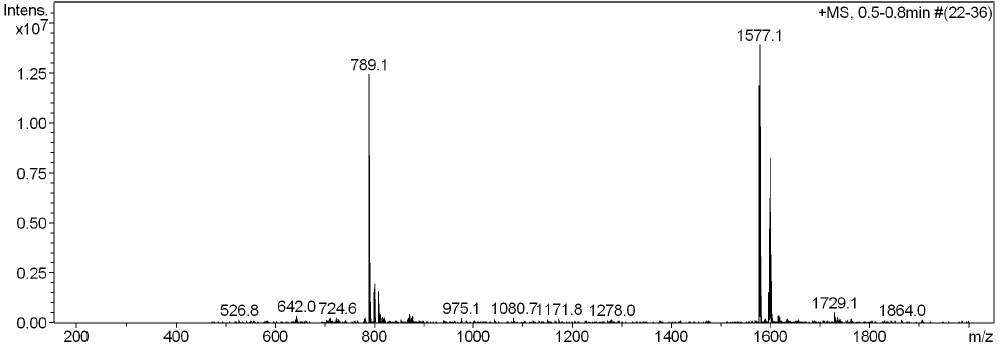


HRMS (*m/z*)


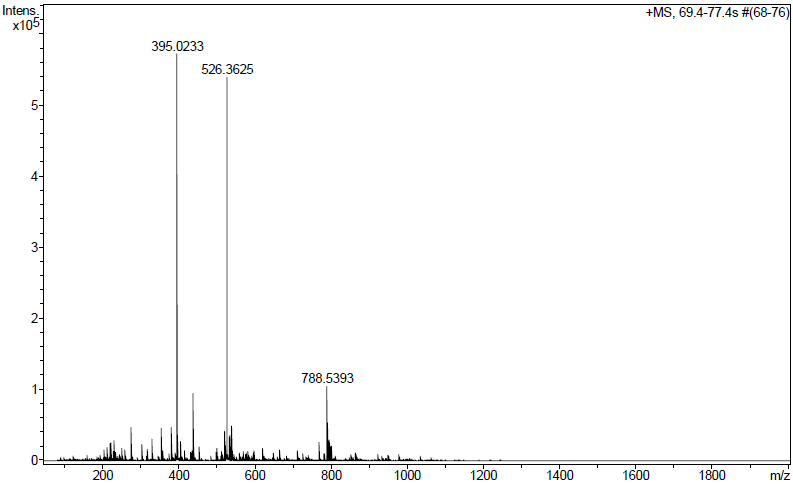


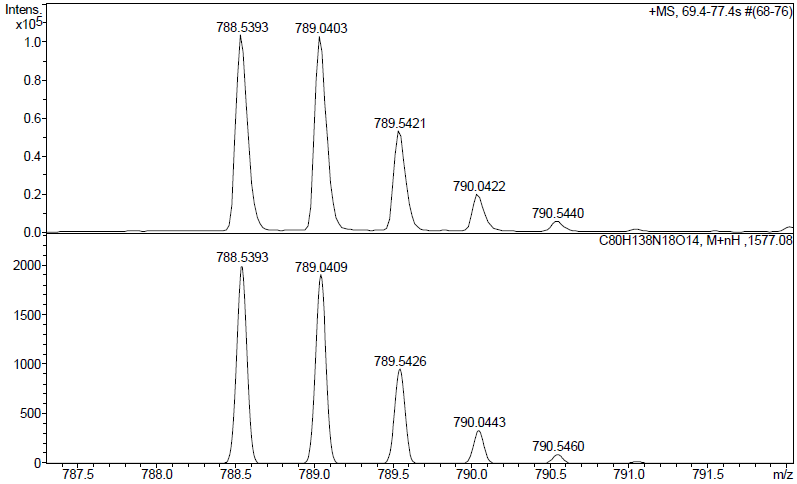


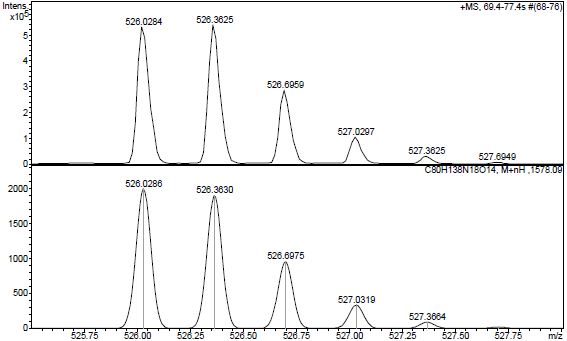


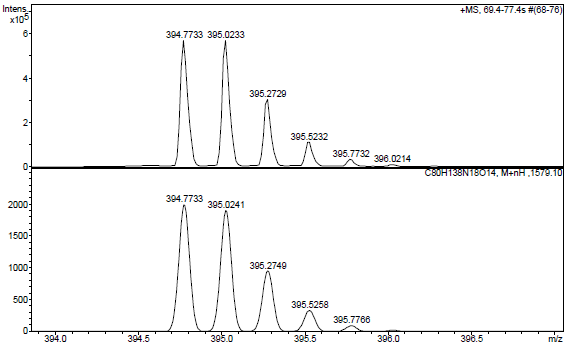


### Ac-Lys-Lys-Leu-Phe-Lys-Lys-Ile-Lys(COC_5_H_11_)-Lys-Tyr-Leu-NH_2_ (BP375)

HPLC of crude peptide (λ=220 nm)


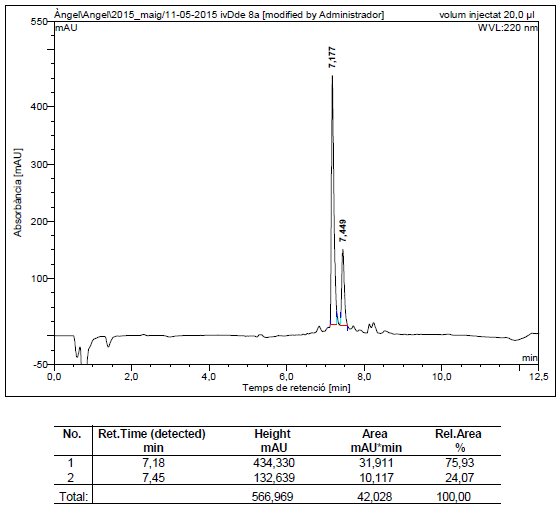


HPLC of purified peptide (λ=220 nm)


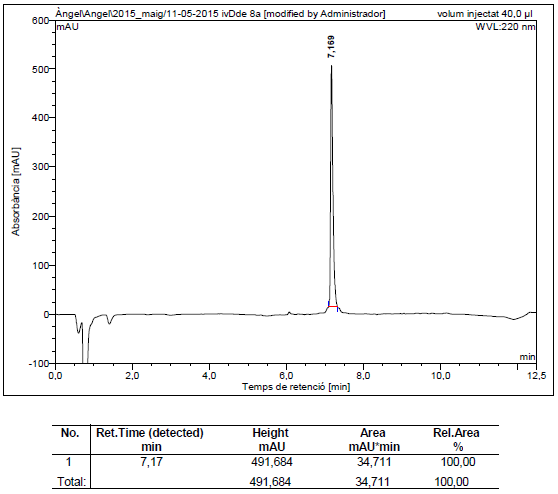


ESI-MS (*m/z*)


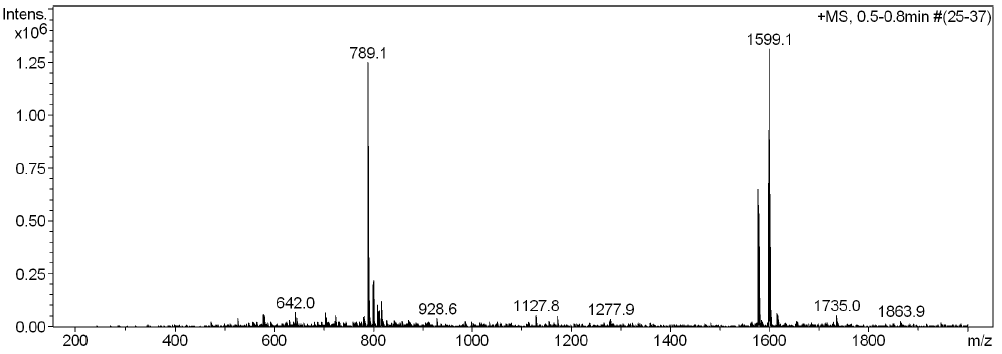


HRMS (*m/z*)


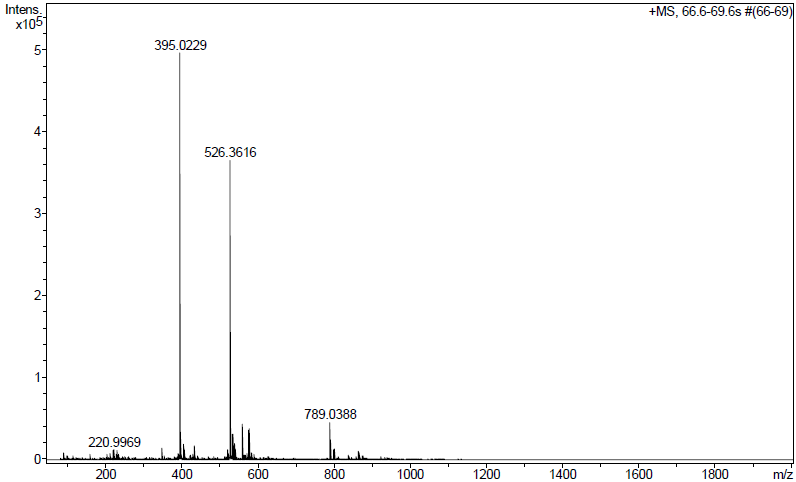


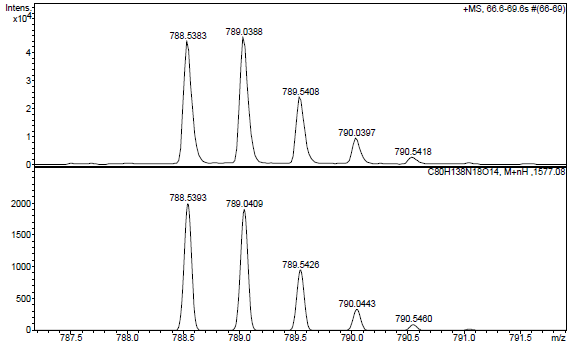


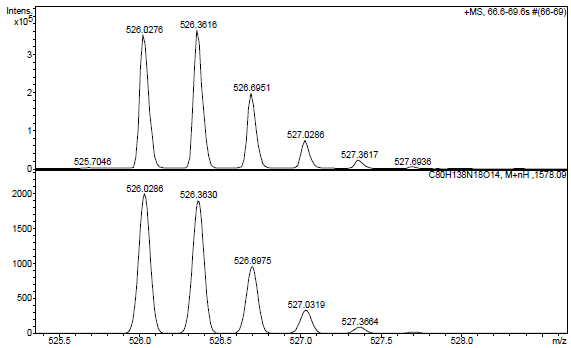


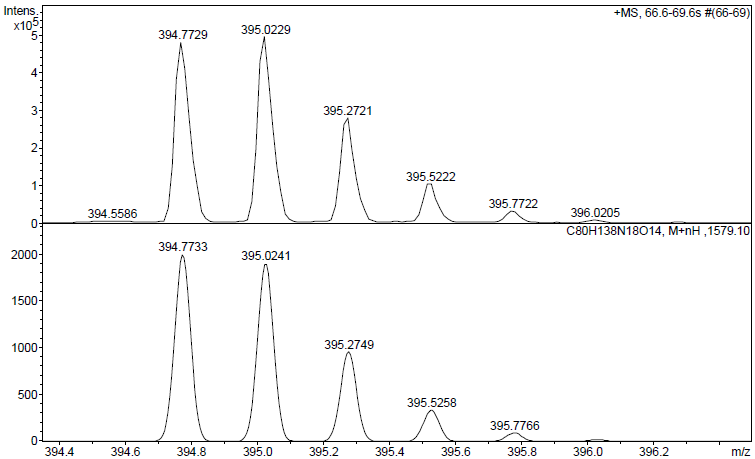


### Ac-Lys-Lys-Leu-Phe-Lys-Lys-Ile-Leu-Lys(COC_5_H_11_)-Tyr-Leu-NH_2_ (BP376)

HPLC of crude peptide (λ=220 nm)


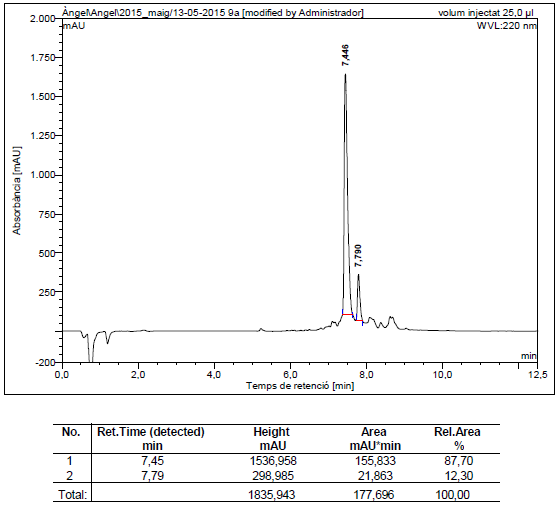


HPLC of purified peptide (λ=220 nm)


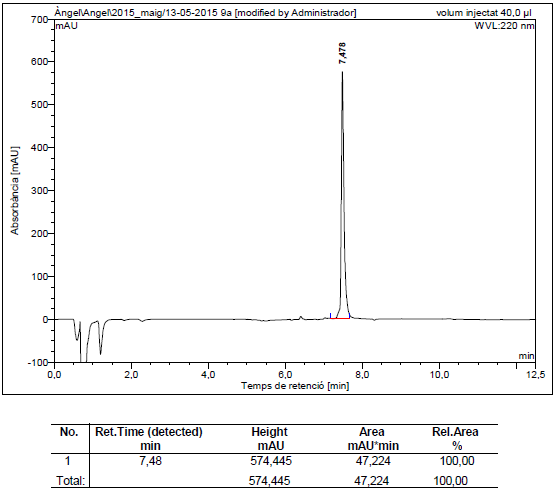


ESI-MS (*m/z*)
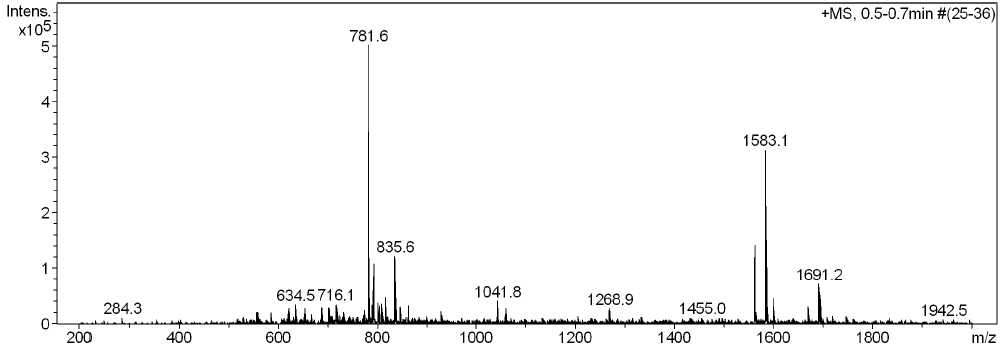


HRMS (*m/z*)


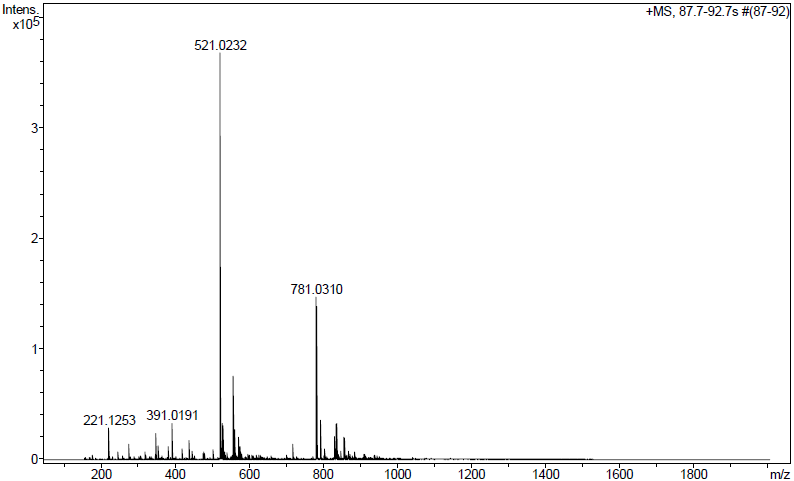


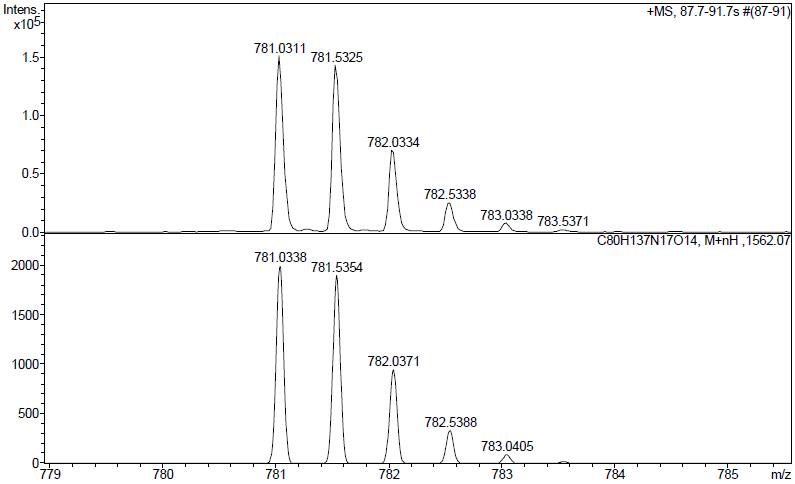


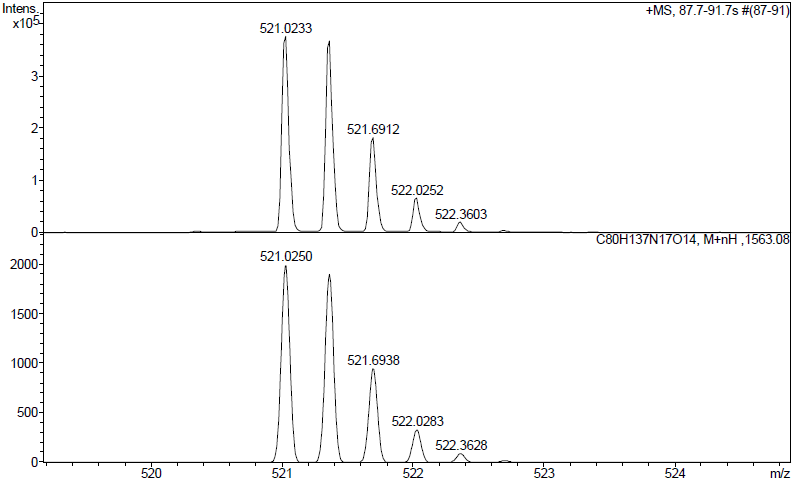


### Ac-Lys-Lys-Leu-Phe-Lys-Lys-Ile-Leu-Lys-Lys(COC_5_H_11_)-Leu-NH_2_ (BP377)

HPLC of crude peptide (λ=220 nm)


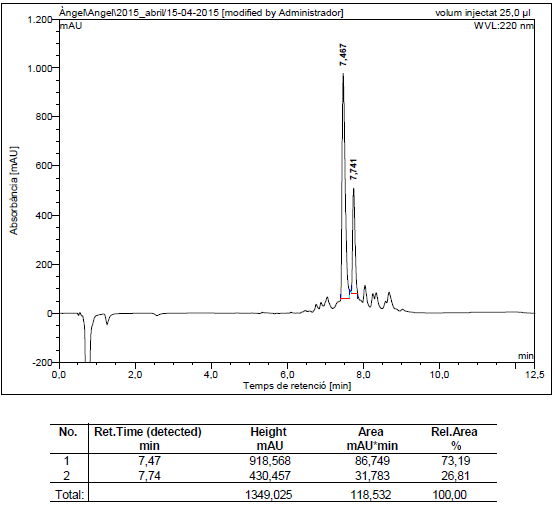


HPLC of purified peptide (λ=220 nm)


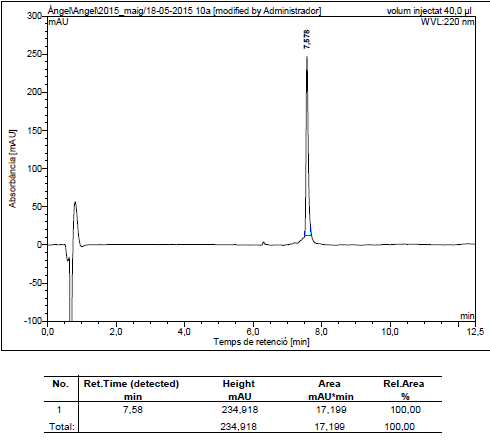


ESI-MS (*m/z*)


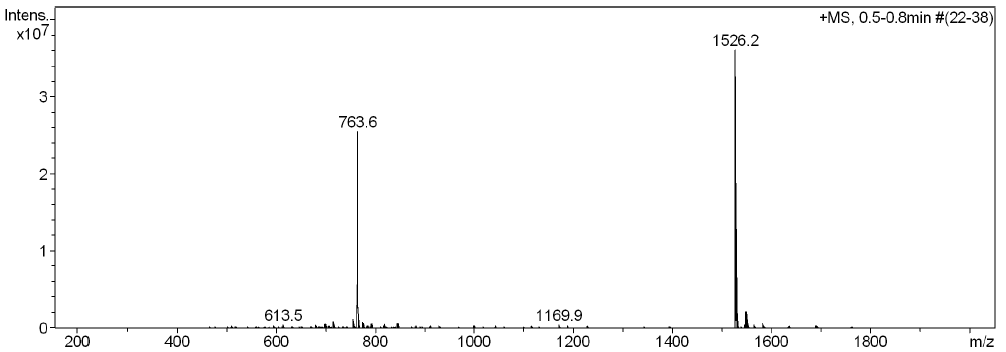


HRMS (*m/z*)


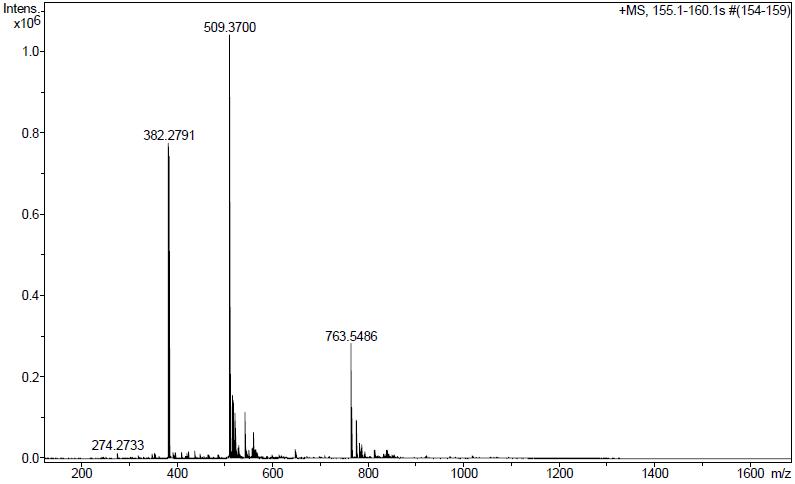


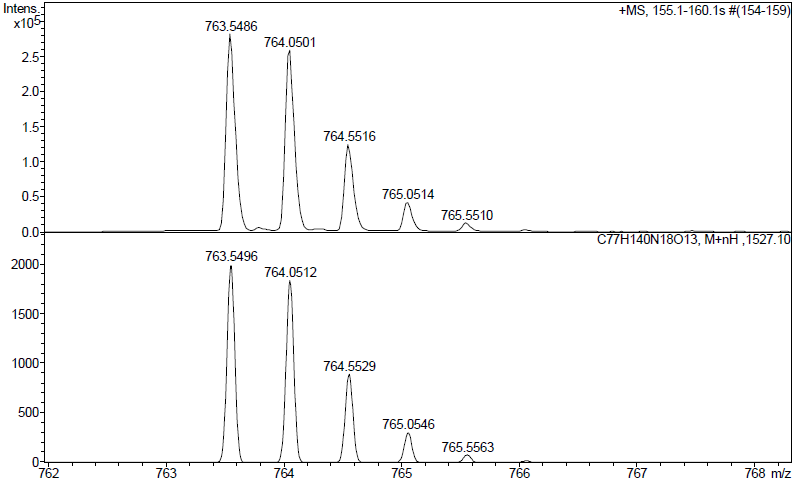


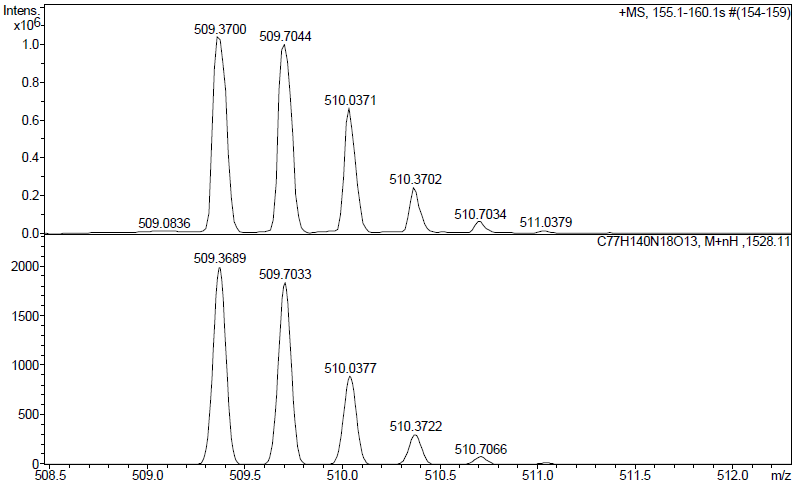


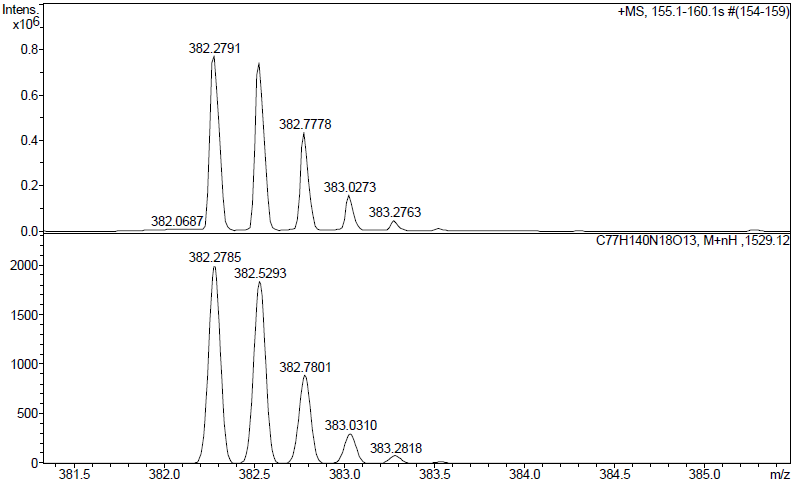


### Ac-Lys-Lys-Leu-Phe-Lys-Lys-Ile-Leu-Lys-Tyr-Lys(COC_5_H_11_)-NH_2_ (BP378)

HPLC of crude peptide (λ=220 nm)


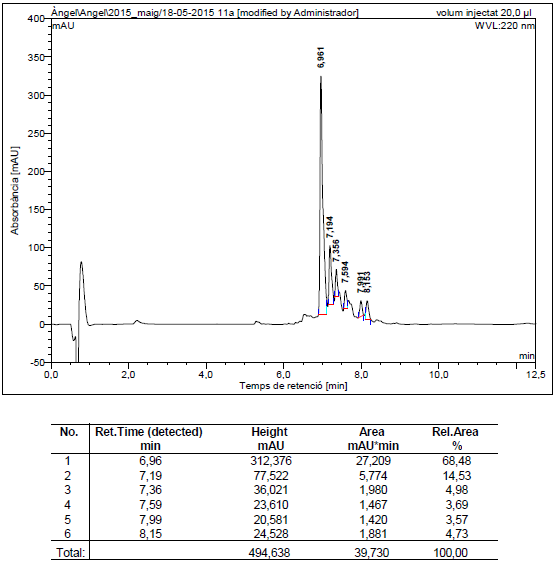


HPLC of purified peptide (λ=220 nm)


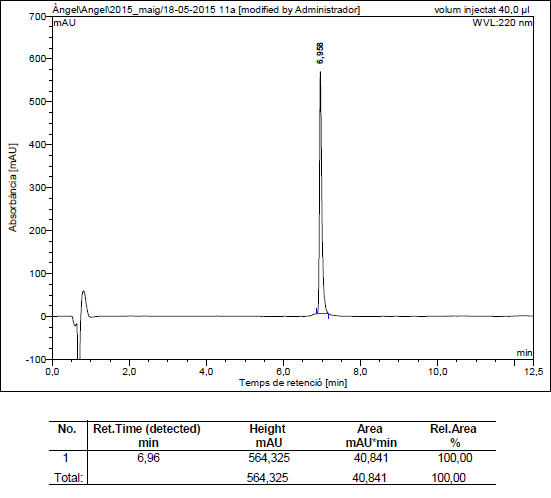


ESI-MS (*m/z*)


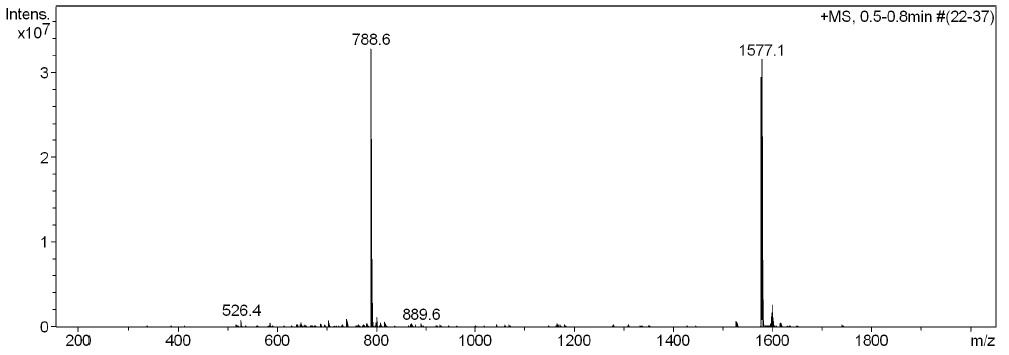


HRMS (*m/z*)


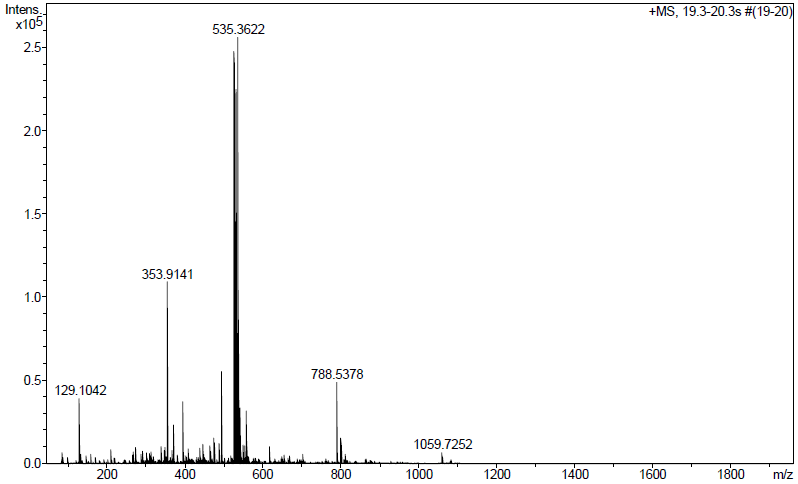


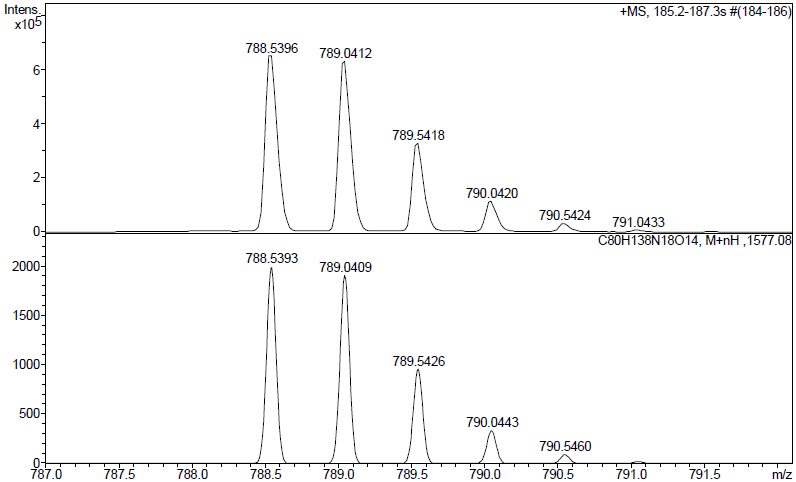


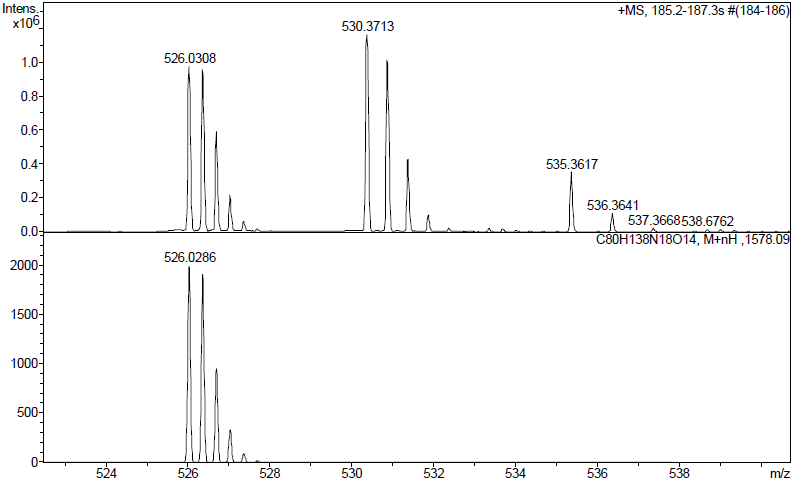


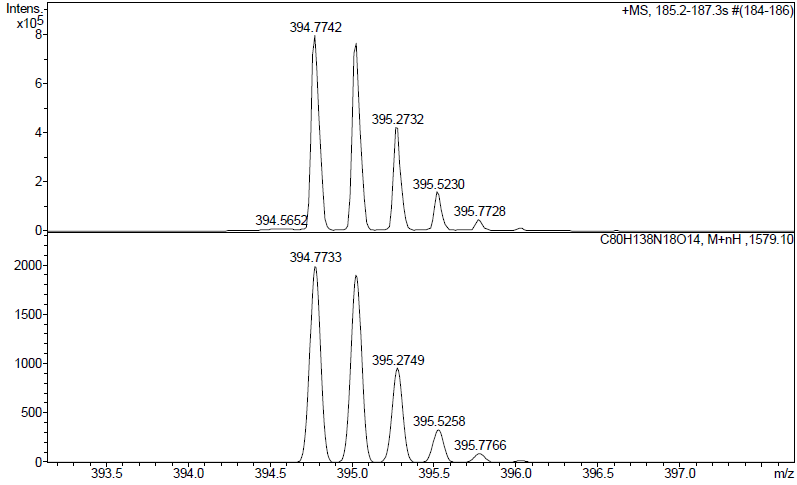


### C_3_H_7_CO-Lys-Lys-Leu-Phe-Lys-Lys-Ile-Leu-Lys-Tyr-Leu-NH_2_ (BP379)

HPLC of crude peptide (λ=220 nm)


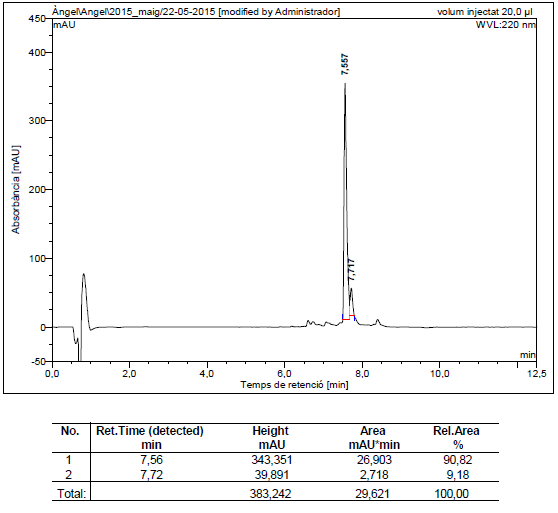


HPLC of purified peptide (λ=220 nm)


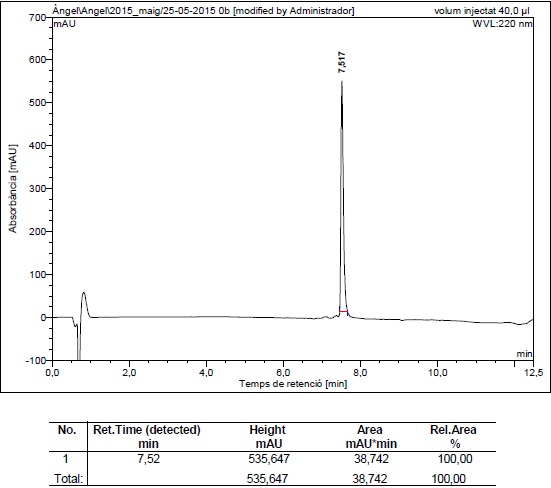


ESI-MS (*m/z*)


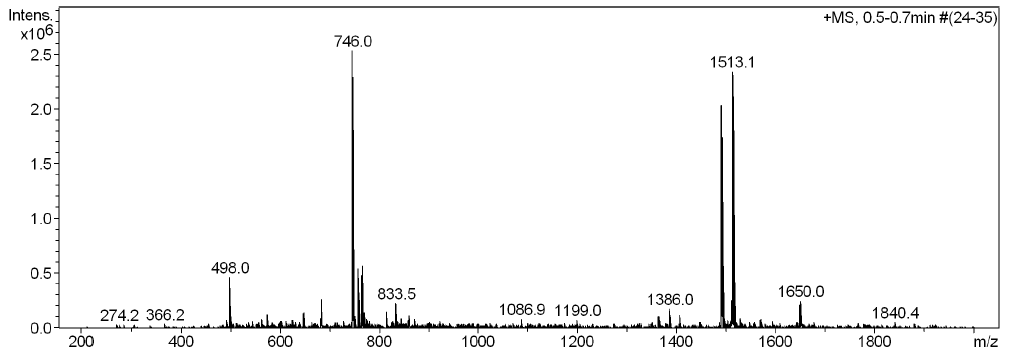


HRMS (*m/z*)


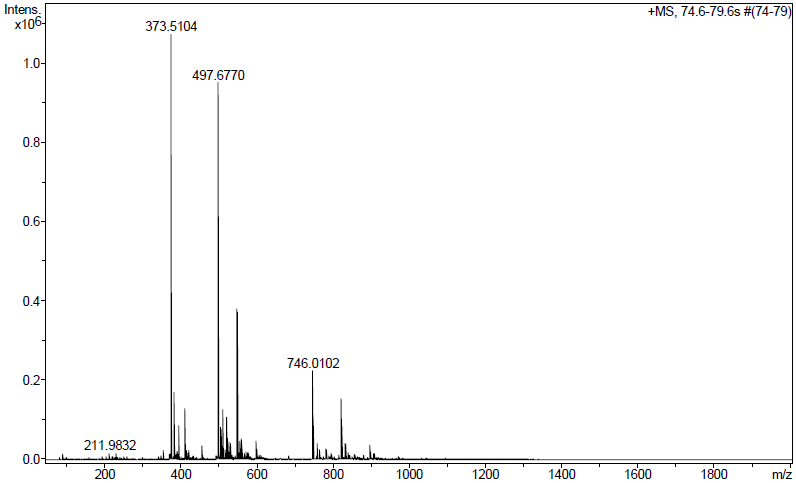


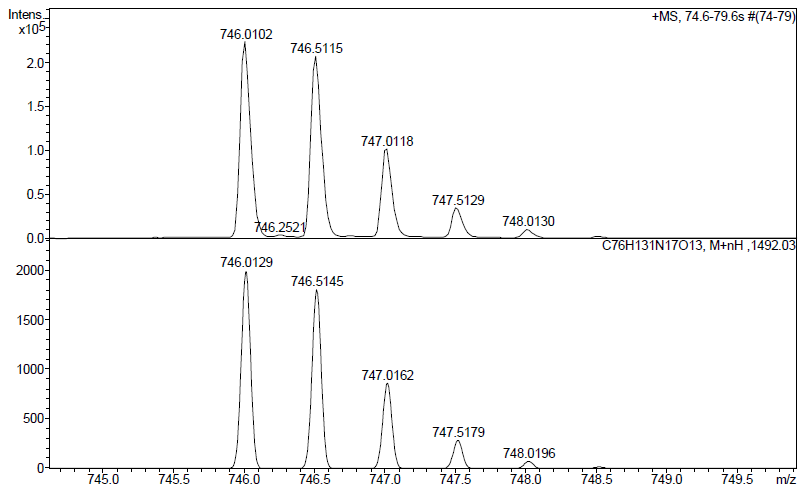


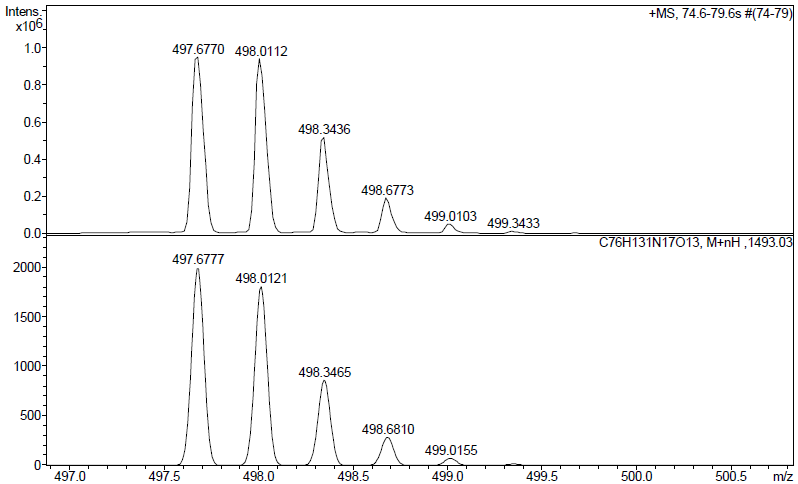


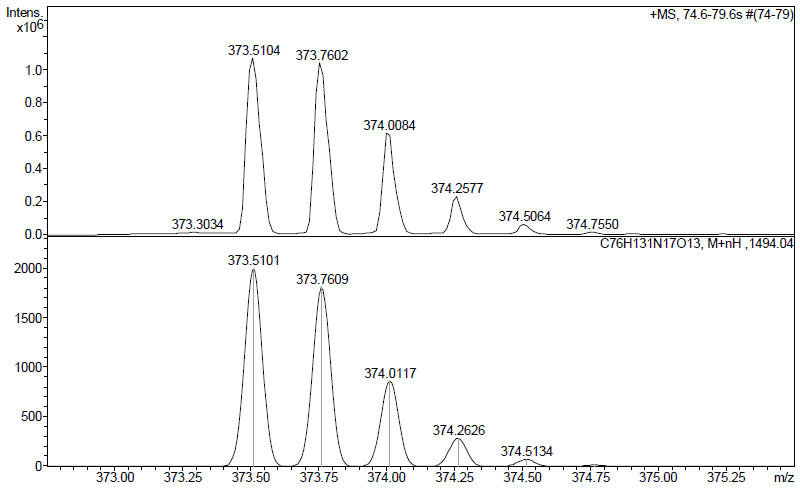


### Ac-Lys(COC_3_H_7_)-Lys-Leu-Phe-Lys-Lys-Ile-Leu-Lys-Tyr-Leu-NH_2_ (BP380)

HPLC of crude peptide (λ=220 nm)


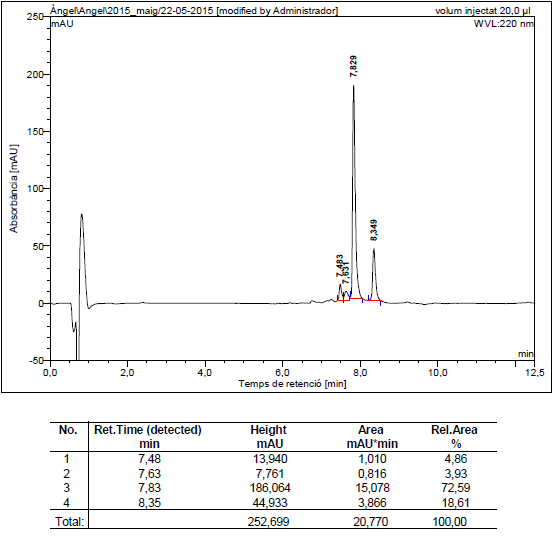


HPLC of purified peptide (λ=220 nm)


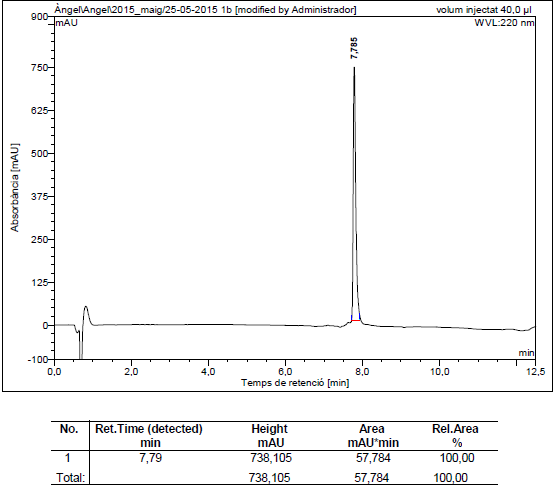


ESI-MS (*m/z*)


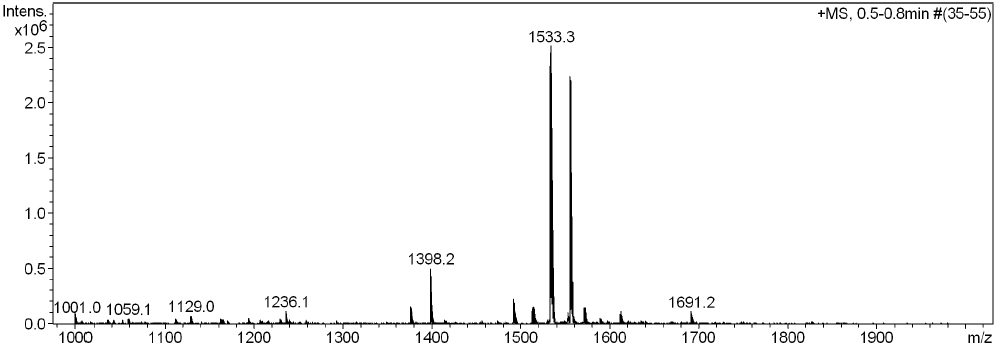


HRMS (*m/z*)


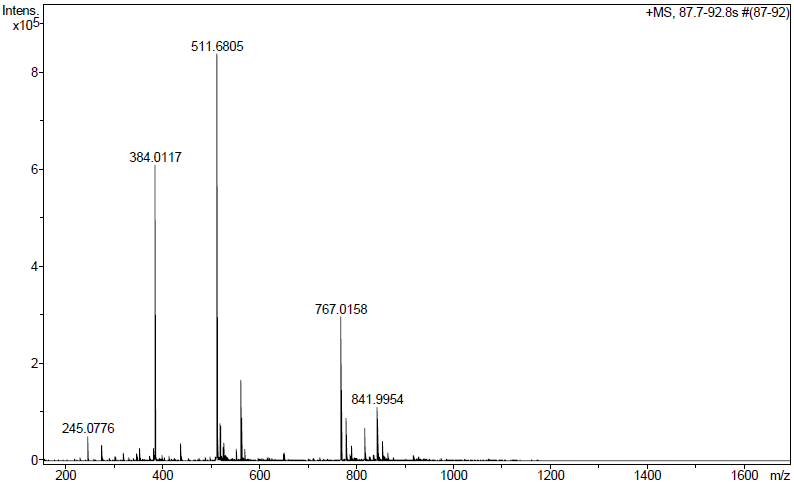


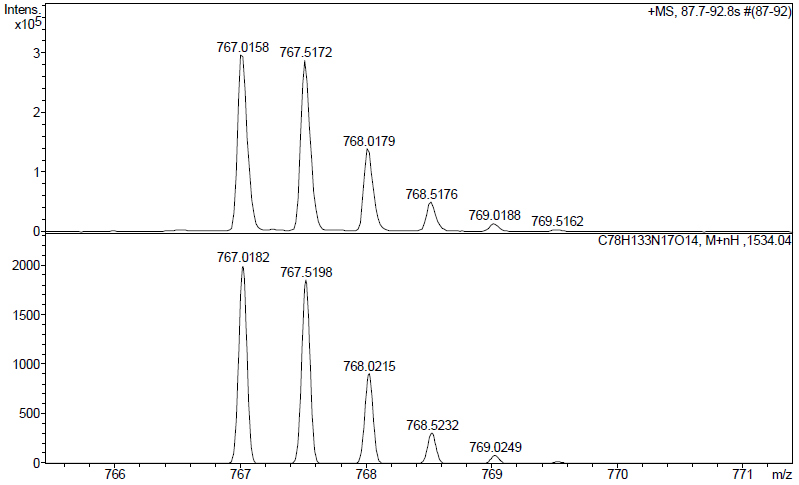


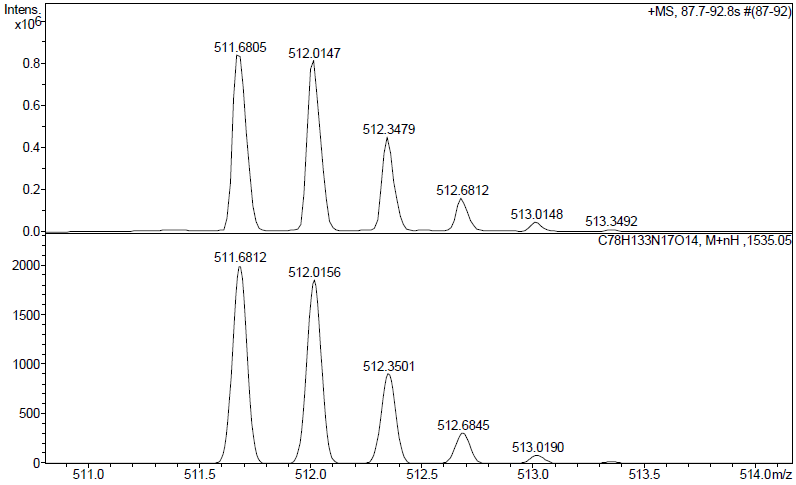


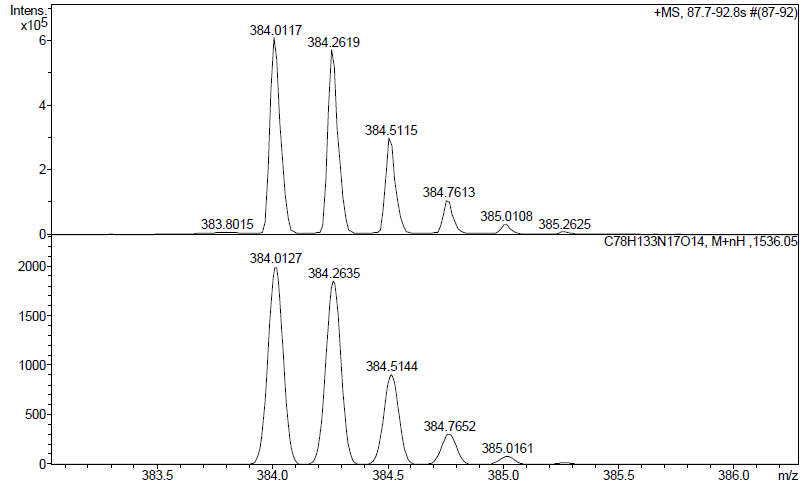


### Ac-Lys-Lys(COC_3_H_7_)-Leu-Phe-Lys-Lys-Ile-Leu-Lys-Tyr-Leu-NH_2_ (BP381)

HPLC of crude peptide (λ=220 nm)

HPLC of purified peptide (λ=220 nm)

ESI-MS (*m/z*)


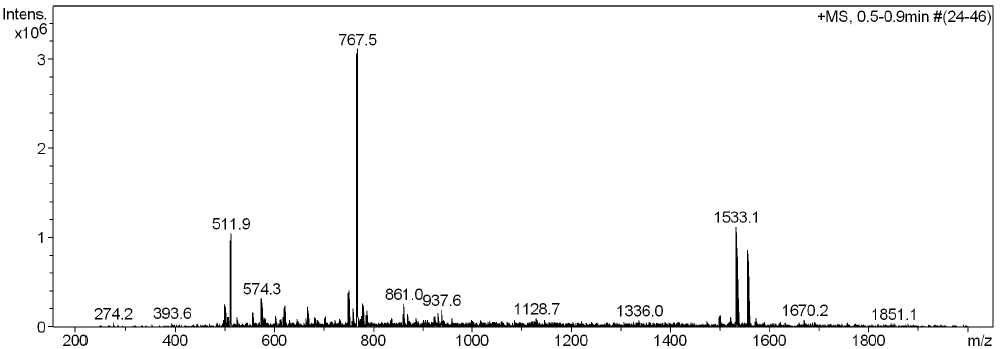


HRMS (*m/z*)


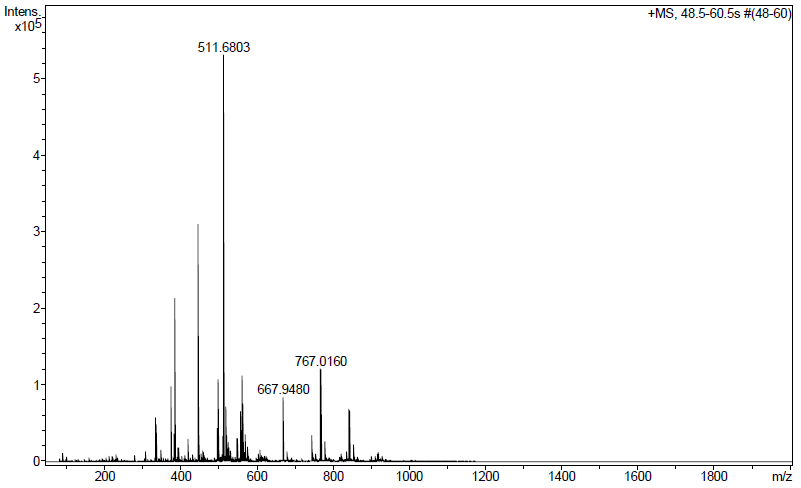


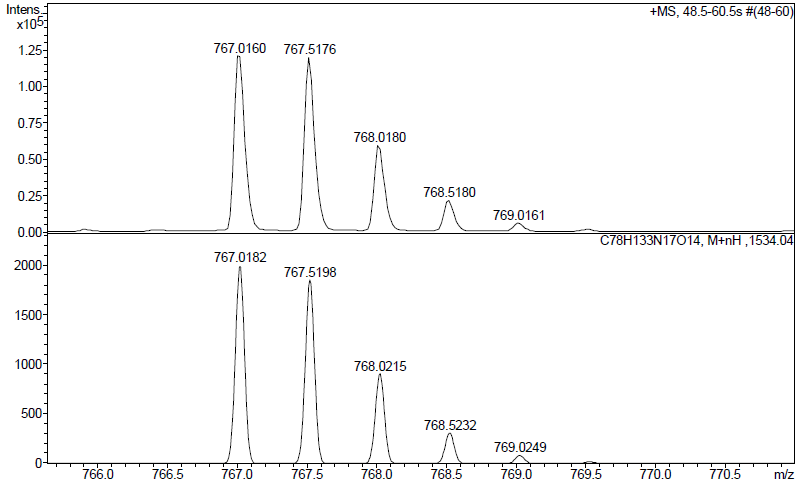


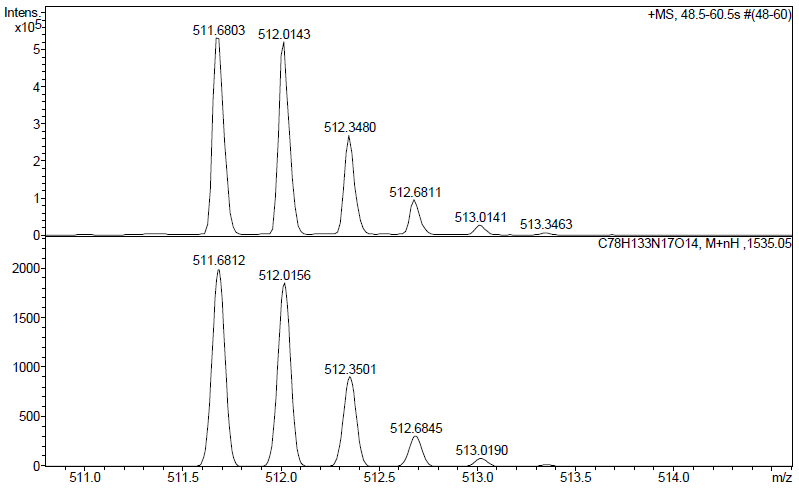


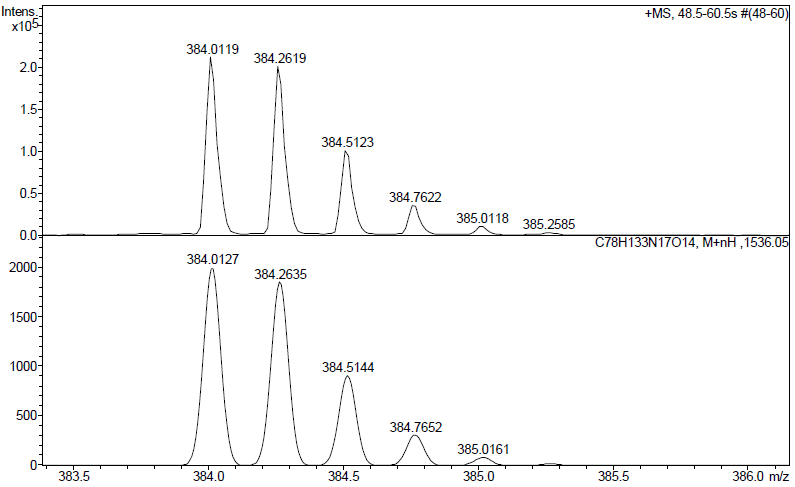


### Ac-Lys-Lys-Lys(COC_3_H_7_)-Phe-Lys-Lys-Ile-Leu-Lys-Tyr-Leu-NH_2_ (BP382)

HPLC of crude peptide (λ=220 nm)

HPLC of purified peptide (λ=220 nm)

ESI-MS (*m/z*)

HRMS (*m/z*)

### Ac-Lys-Lys-Leu-Lys(COC_3_H_7_)-Lys-Lys-Ile-Leu-Lys-Tyr-Leu-NH_2_ (BP383)

HPLC of crude peptide (λ=220 nm)

HPLC of purified peptide (λ=220 nm)

ESI-MS (*m/z*)

HRMS (*m/z*)

### Ac-Lys-Lys-Leu-Phe-Lys(COC_3_H_7_)-Lys-Ile-Leu-Lys-Tyr-Leu-NH_2_ (BP384)

HPLC of crude peptide (λ=220 nm)

HPLC of purified peptide (λ=220 nm)

ESI-MS (*m/z*)

HRMS (*m/z*)

### Ac-Lys-Lys-Leu-Phe-Lys-Lys(COC_3_H_7_)-Ile-Leu-Lys-Tyr-Leu-NH_2_ (BP385)

HPLC of crude peptide (λ=220 nm)

HPLC of purified peptide (λ=220 nm)

ESI-MS (*m/z*)

HRMS (*m/z*)

### Ac-Lys-Lys-Leu-Phe-Lys-Lys-Lys(COC_3_H_7_)-Leu-Lys-Tyr-Leu-NH_2_ (BP386)

HPLC of crude peptide (λ=220 nm)

HPLC of purified peptide (λ=220 nm)

ESI-MS (*m/z*)

HRMS (*m/z*)

### Ac-Lys-Lys-Leu-Phe-Lys-Lys-Ile-Lys(COC_3_H_7_)-Lys-Tyr-Leu-NH_2_ (BP387)

HPLC of crude peptide (λ=220 nm)

HPLC of purified peptide (λ=220 nm)

ESI-MS (*m/z*)

HRMS (*m/z*)

### Ac-Lys-Lys-Leu-Phe-Lys-Lys-Ile-Leu-Lys(COC_3_H_7_)-Tyr-Leu-NH_2_ (BP388)

HPLC of crude peptide (λ=220 nm)

HPLC of purified peptide (λ=220 nm)

ESI-MS (*m/z*)

HRMS (*m/z*)

### Ac-Lys-Lys-Leu-Phe-Lys-Lys-Ile-Leu-Lys-Lys(COC_3_H_7_)-Leu-NH_2_ (BP389)

HPLC of crude peptide (λ=220 nm)

HPLC of purified peptide (λ=220 nm)

ESI-MS (*m/z*)

HRMS (*m/z*)

### Ac-Lys-Lys-Leu-Phe-Lys-Lys-Ile-Leu-Lys-Tyr-Lys(COC_3_H_7_)-NH_2_ (BP390)

HPLC of crude peptide (λ=220 nm)

HPLC of purified peptide (λ=220 nm)

ESI-MS (*m/z*)

HRMS (*m/z*)

### C_11_H_23_CO-Lys-Lys-Leu-Phe-Lys-Lys-Ile-Leu-Lys-Tyr-Leu-NH_2_ (BP391)

HPLC of crude peptide (λ=220 nm)

HPLC of purified peptide (λ=220 nm)

ESI-MS (*m/z*)

HRMS (*m/z*)

### Ac-Lys(COC_11_H_23_)-Lys-Leu-Phe-Lys-Lys-Ile-Leu-Lys-Tyr-Leu-NH_2_ (BP392)

HPLC of crude peptide (λ=220 nm)

HPLC of purified peptide (λ=220 nm)

ESI-MS (*m/z*)

HRMS (*m/z*)

### Ac-Lys-Lys(COC_11_H_23_)-Leu-Phe-Lys-Lys-Ile-Leu-Lys-Tyr-Leu-NH_2_ (BP393)

HPLC of crude peptide (λ=220 nm)

HPLC of purified peptide (λ=220 nm)

ESI-MS (*m/z*)

HRMS (*m/z*)

### Ac-Lys-Lys-Lys(COC_11_H_23_)-Phe-Lys-Lys-Ile-Leu-Lys-Tyr-Leu-NH_2_ (BP394)

HPLC of crude peptide (λ=220 nm)

HPLC of purified peptide (λ=220 nm)

ESI-MS (*m/z*)

HRMS (*m/z*)

### Ac-Lys-Lys-Leu-Lys(COC_11_H_23_)-Lys-Lys-Ile-Leu-Lys-Tyr-Leu-NH_2_ (BP395)

HPLC of crude peptide (λ=220 nm)

HPLC of purified peptide (λ=220 nm)

ESI-MS (*m/z*)

HRMS (*m/z*)

### Ac-Lys-Lys-Leu-Phe-Lys(COC_11_H_23_)-Lys-Ile-Leu-Lys-Tyr-Leu-NH_2_ (BP396)

HPLC of crude peptide (λ=220 nm)

HPLC of purified peptide (λ=220 nm)

ESI-MS (*m/z*)

HRMS (*m/z*)

### Ac-Lys-Lys-Leu-Phe-Lys-Lys(COC_11_H_23_)-Ile-Leu-Lys-Tyr-Leu-NH_2_ (BP397)

HPLC of crude peptide (λ=220 nm)

HPLC of purified peptide (λ=220 nm)

ESI-MS (*m/z*)

HRMS (*m/z*)

### Ac-Lys-Lys-Leu-Phe-Lys-Lys-Lys(COC_11_H_23_)-Leu-Lys-Tyr-Leu-NH_2_ (BP398)

HPLC of crude peptide (λ=220 nm)

HPLC of purified peptide (λ=220 nm)

ESI-MS (*m/z*)

HRMS (*m/z*)

### Ac-Lys-Lys-Leu-Phe-Lys-Lys-Ile-Lys(COC_11_H_23_)-Lys-Tyr-Leu-NH_2_ (BP399)

HPLC of crude peptide (λ=220 nm)

HPLC of purified peptide (λ=220 nm)

ESI-MS (*m/z*)

HRMS (*m/z*)

### Ac-Lys-Lys-Leu-Phe-Lys-Lys-Ile-Leu-Lys(COC_11_H_23_)-Tyr-Leu-NH_2_ (BP400)

HPLC of crude peptide (λ=220 nm)

HPLC of purified peptide (λ=220 nm)

ESI-MS (*m/z*)

HRMS (*m/z*)

### Ac-Lys-Lys-Leu-Phe-Lys-Lys-Ile-Leu-Lys-Lys(COC_11_H_23_)-Leu-NH_2_ (BP401)

HPLC of crude peptide (λ=220 nm)

HPLC of purified peptide (λ=220 nm)

ESI-MS (*m/z*)

HRMS (*m/z*)

### Ac-Lys-Lys-Leu-Phe-Lys-Lys-Ile-Leu-Lys-Tyr-Lys(COC_11_H_23_)-NH_2_ (BP402)

HPLC of crude peptide (λ=220 nm)

HPLC of purified peptide (λ=220 nm)

ESI-MS (*m/z*)

HRMS (*m/z*)
